# Delayed electron–hole recombination in a pseudo-direct semiconductor

**DOI:** 10.1038/s41586-026-11223-x

**Published:** 2026-10-28

**Authors:** Marcello Righetto, Debalina Banerjee, Zhenglin Jia, Joshua R. S. Lilly, Vincent J. -Y. Lim, Snigdha Lal, Siyu Yan, Jae Eun Lee, Jay B. Patel, Michael B. Johnston, Qianqian Lin, Marina R. Filip, Laura M. Herz

**Affiliations:** 1 Department of Physics, University of Oxford, Clarendon Laboratory, Oxford, UK; 2 Department of Chemical Sciences, Università degli Studi di Padova, Padova, Italy; 3 Key Lab of Artificial Micro- and Nano-Structures of Ministry of Education of China, School of Physics and Technology, Wuhan University, Wuhan, China; 4 Department of Physics, King’s College London, London, UK

**Keywords:** Solar cells, Solar cells, Semiconductors

## Abstract

Kirchhoff demonstrated the reciprocity between absorptivity and emissivity for blackbody radiation in 1860 (ref. ^1^), thereby establishing the general principle that a good absorber must also be a good emitter. This principle underpins the generalized Planck’s law^2^ and, when applied to semiconductors—in the form of the van Roosbroeck–Shockley equation^3^—results in substantial limitations to solar energy conversion. Here we demonstrate how this reciprocity can be circumvented in semiconductors, such as bismuth sulfide, showing a pseudo-direct bandgap character—that is, featuring a lower-energy indirect transition paired with a direct transition placed a few *k*_B_*T* above. After determining the pseudo-direct bandgap nature of bismuth sulfide, we reveal how this feature substantially retards bimolecular electron–hole recombination in this material with respect to classic direct semiconductors^4^. The resulting combination of a high absorption coefficient and delayed recombination offers the unprecedented possibility of overcoming limitations associated with Planck’s law, with far-reaching potential for solar energy conversion.

## Abstract (web summary)

Pseudo-direct bandgap character in bismuth sulfide retards bimolecular electron–hole recombination while preserving strong light absorption.

## Main

Charge-carrier recombination in semiconductors is a fundamental process determining device efficiencies for a variety of key optoelectronic applications, ranging from photovoltaics^5,6^ to light-emission^7,8^ and photo(electro)catalysis^9,10^. Several intrinsic (for example, bimolecular) and extrinsic (for example, trap-mediated) recombination mechanisms contribute to charge-carrier recombination in light-harvesting devices such as photovoltaic and photochemical cells, in which recombination competes with charge-carrier extraction^5,6,11^ and thus causes efficiency losses. For any solar absorber material, minimizing extrinsic recombination losses is therefore a powerful first-line strategy to increase power conversion efficiencies^12^, with any remaining intrinsic losses defined by the detailed balance limit. For example, the benign defect chemistry of lead halide perovskites^13^ and the use of molecular passivation strategies^12^ have achieved trap-mediated recombination rates as low as *k*_1_ ≈ 10^5^ s^−1^ (refs. ^7,8^). As a result, power conversion efficiencies of perovskite solar cells now reach values of about 28% (ref. ^14^). Similarly, more established technologies based around silicon or III–V semiconductors are reaching their intrinsic performance limits^14,15^ in solar devices.

These rapid developments mean that further minimization of charge-carrier recombination is now mostly hampered by Planck’s law, which sets inherent limitations to the reduction of intrinsic bimolecular electron–hole recombination rates^4,16^. In lead halide perovskites, similar to GaAs and Si, the intrinsic bimolecular recombination process of photogenerated electrons with holes is an inverse absorption process and has been demonstrated to follow the van Roosbroeck–Shockley (vRS) relation closely^3,4,17,18^. Therefore, the high bimolecular recombination rates reported for lead halide perovskites (LHP) and GaAs^11,19^ (*k*_2,LHP _≈ 10^−9^ cm^3^ s^−1^, *k*_2,GaAs _≈ 10^−10^ cm^3^ s^−1^) are a direct consequence of their direct bandgap character and the ensuing high absorption coefficients, which, taken on their own, are a desirable property for solar absorber materials. However, the associated high bimolecular recombination rates severely limit applications of perovskites and other highly absorbing semiconductors not only in solar cells but also in photoelectrocatalytic cells^9,10^. In these applications, extended charge-carrier lifetimes at semiconductor–electrolyte interfaces are vital for solar energy conversion to chemicals^20^.

Although the generalized Planck’s law sets a strict dependency between absorption and charge-carrier recombination efficiencies, these considerations apply only when both these processes originate from the same electronic state^3^. Decoupling light absorption and charge-carrier recombination processes is, therefore, a promising strategy to improve solar conversion efficiencies. This decoupling has generally been achieved only by sacrificing a considerable fraction of the photovoltage. Natural photosynthetic systems, for instance, trade around 0.7 eV in photovoltage to increase charge-carrier recombination lifetimes by six orders of magnitude^20^. A similar approach has been proposed in photoelectrochemical cells using polaron and electronic defect states in inorganic semiconductors, yet these strategies profoundly disrupt charge-carrier transport and, hence, increase efficiency losses substantially^9,21,22^. By contrast, Rashba splitting (that is, a spin–orbit-induced splitting in the *k*-space) has been proposed as a potential mechanism that could impart an indirect character to perovskites and thus yield long charge-carrier lifetimes^23^. However, several works demonstrated the direct bandgap nature of bulk halide perovskites and revealed the negligible part played by Rashba-related effects in recombination^4,24–26^, therefore leaving the quest for delayed recombination in semiconductors open.

Here, we show that bimolecular electron–hole recombination dynamics in Bi_2_S_3_ thin films deviate from the standard predictions of vRS theory for either direct or indirect semiconductors. By combining experimental and computational approaches, we determine the absorption properties of Bi_2_S_3_ and reveal a mixed direct–indirect bandgap character. We demonstrate that this peculiar electronic band structure, with a lowest-energy indirect transition paired with a direct transition placed only a few tens of meV above, profoundly influences charge-carrier recombination in Bi_2_S_3_. Our temperature-resolved study of electron–hole recombination in Bi_2_S_3_ shows that the thermal equilibrium between direct and indirect transitions governs bimolecular recombination in this material. As a result, Bi_2_S_3_ offers an ideal combination comprising the best properties of both indirect and direct semiconductors, that is, retarded electron–hole recombination and high absorption coefficients, while sacrificing only minute potential photovoltage losses. Therefore, we propose Bi_2_S_3_ as the first candidate for a new and long-sought type of semiconductor—pseudo-direct semiconductors—which hold the potential to deliver efficient solar energy conversion.

## Light absorption in Bi_2_S_3_ thin films

**Fig. 1:**
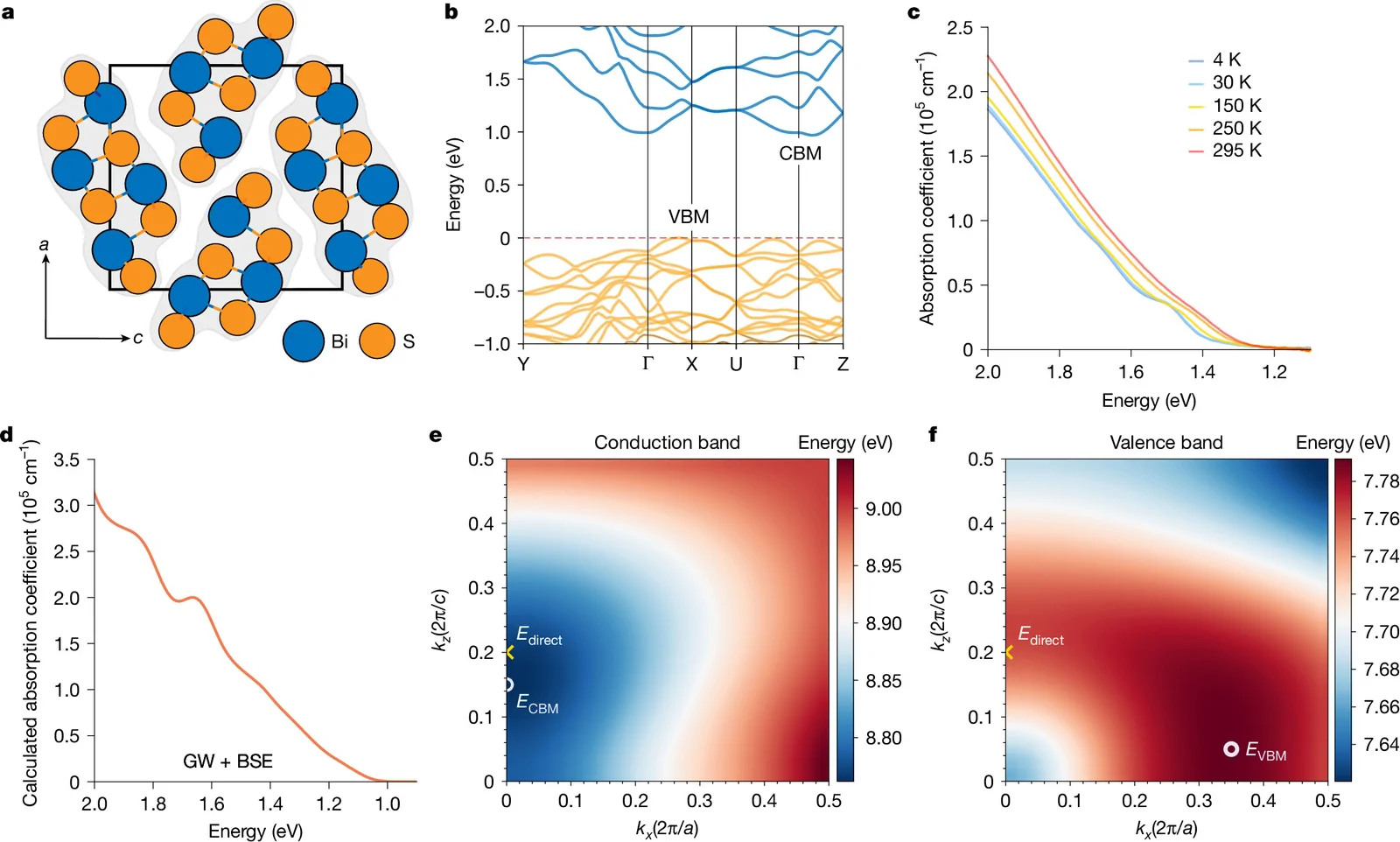
Electronic structure and absorption coefficient of Bi_2_S_3_ thin films. **a**, Schematic of the Bi_2_S_3_ crystal structure (*Pnma* space group) along the [010] direction. The grey-shaded regions indicate the ribbon structures. The black square indicates the unit cell, containing two distinct ribbons. The blue and orange spheres represent bismuth and sulfur atoms, respectively. **b**, Electronic band structure for Bi_2_S_3_, as calculated from DFT/PBE + SOC on the following high-symmetry path: Y(0, 1/2, 0) → Γ(0, 0, 0) → X(1/2, 0, 0) → U(1/2, 0, 1/2) → Γ(0, 0, 0) → Z(0, 0, 1/2), in units of the reciprocal lattice. The zero of the energy axis is set to the top of the valence band and indicated by a red dashed line. Low-energy direct electronic transitions are shown on the electronic band structure in Extended Data Fig. 1a. **c**, Absorption coefficient for Bi_2_S_3_ thin films on quartz, measured in the NIR–Vis range for selected temperatures between 4 K and 295 K. The complete temperature-dependent absorption coefficient dataset is reported in Extended Data Fig. 2a. **d**, The calculated linear optical absorption spectrum including only direct transitions, computed within the GW + Bethe–Salpeter equation (BSE) framework, including SOC, as described in detail in the Supplementary Information. The onset of the calculated spectrum is red-shifted with respect to the measured onset by approximately 0.3 eV, consistent with typical underestimated quasiparticle band gaps computed within the G_0_W_0_ with a DFT/PBE starting point, extensively documented in the literature^60^. **e**,**f**, Calculated DFT/PBE (including SOC) electronic band structure on the *k*_*x*_–*k*_*z*_ Brillouin zone plane. White circles are used to indicate the position of the valence band maximum (VBM) and the conduction band minimum (CBM). A yellow mark is used to indicate the position of the lowest-energy direct transition. The corresponding direct transition energies are shown in Extended Data Fig. 1b.

**Extended Data Fig. 1:**
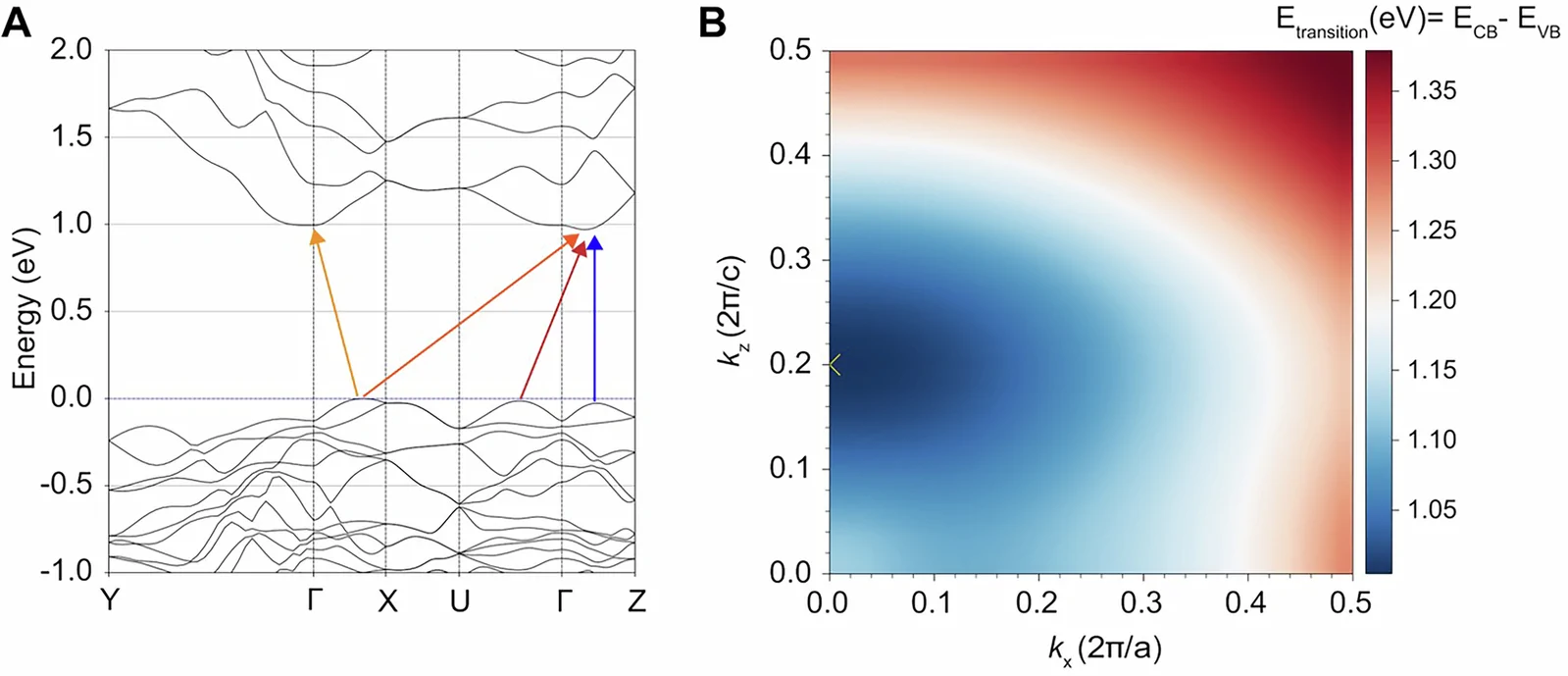
Electronic transitions in Bi_2_S_3_. (**A**) Electronic band structure for Bi_2_S_3_, as calculated within DFT/PBE + SOC, on the following high-symmetry path: Y(0, 1/2, 0) - Γ(0, 0, 0) - X(1/2, 0, 0) - U(1/2, 0, 1/2) - Γ(0, 0, 0) - Z(0, 0, 1/2), in units of the reciprocal lattice. The blue dashed line indicates the highest occupied energy level and is arbitrarily set to zero energy. The orange and red arrows represent the low-lying (energetically) electronic transitions between the multiple valence band maxima and conduction band minima. The blue arrow represents the electronic transition corresponding to the direct gap. (**B**) Calculated direct transition energies in the Γ, X, U and Z regions of the Brillouin zone, plotted in reciprocal lattice vector units 2πa^−1^ and 2πc^−1^ for the *k*_x_ and *k*_z_ axes, respectively, where a and c are the lattice parameters of Bi_2_S_3_. Transition energies are calculated as the difference between the conduction and valence band energies, shown in Fig. 1e,f of the main text. The yellow cross symbol indicates the lowest energy direct transition point, i.e. the direct gap.

We focus on bismuth sulfide (Bi_2_S_3_), an emerging non-toxic and stable semiconductor with promising photocatalytic, photodetector and energy-harvesting performance and an absorption onset at about 1.4 eV, ideal for single-junction photovoltaics^27–29^. We fabricated Bi_2_S_3_ thin films by thermal evaporation of Bi_2_S_3_ powders on ST-cut quartz substrates^30^. As shown in Fig. 1a, Bi_2_S_3_ crystallizes in the orthorhombic stibnite structure (*Pnma*), which comprises parallel quasi-one-dimensional (Bi_4_S_6_)_*n*_ ribbons bound by strong van der Waals interactions^31^. The bandgap character of Bi_2_S_3_ is debated in the literature, with several experimental and theoretical studies of Bi_2_S_3_ stipulating both direct and indirect bandgap nature^29,32–34^. Although recent work suggests the indirect bandgap nature of Bi_2_S_3_ (ref. ^32^), the reported high absorption coefficients (above 10^5^ cm^−1^) cannot originate from indirect transitions^35^, which are substantially weakened by the requirement of phonon contributions to bridge momentum space. To determine the nature of the Bi_2_S_3_ bandgap, we performed first-principles calculations based on density functional theory (DFT)^36,37^ (Fig. 1b, Methods and Supplementary Information). Our calculations indicate that several bands and valleys of Bi_2_S_3_—around the Γ, X and Z points of the Brillouin zone for the valence band and around Γ and Z for the conduction band—are closely spaced in energy by up to 2*k*_B_*T* and 7*k*_B_*T* for the valence and conduction band edges, respectively, where *T* is room temperature. These multiple band extrema give rise to several energetically closely spaced direct and indirect transitions, as shown in Extended Data Fig. 1a, which probably fuelled the debate and contradictory claims in the literature around the nature of the bandgap in Bi_2_S_3_.

**Extended Data Fig. 2:**
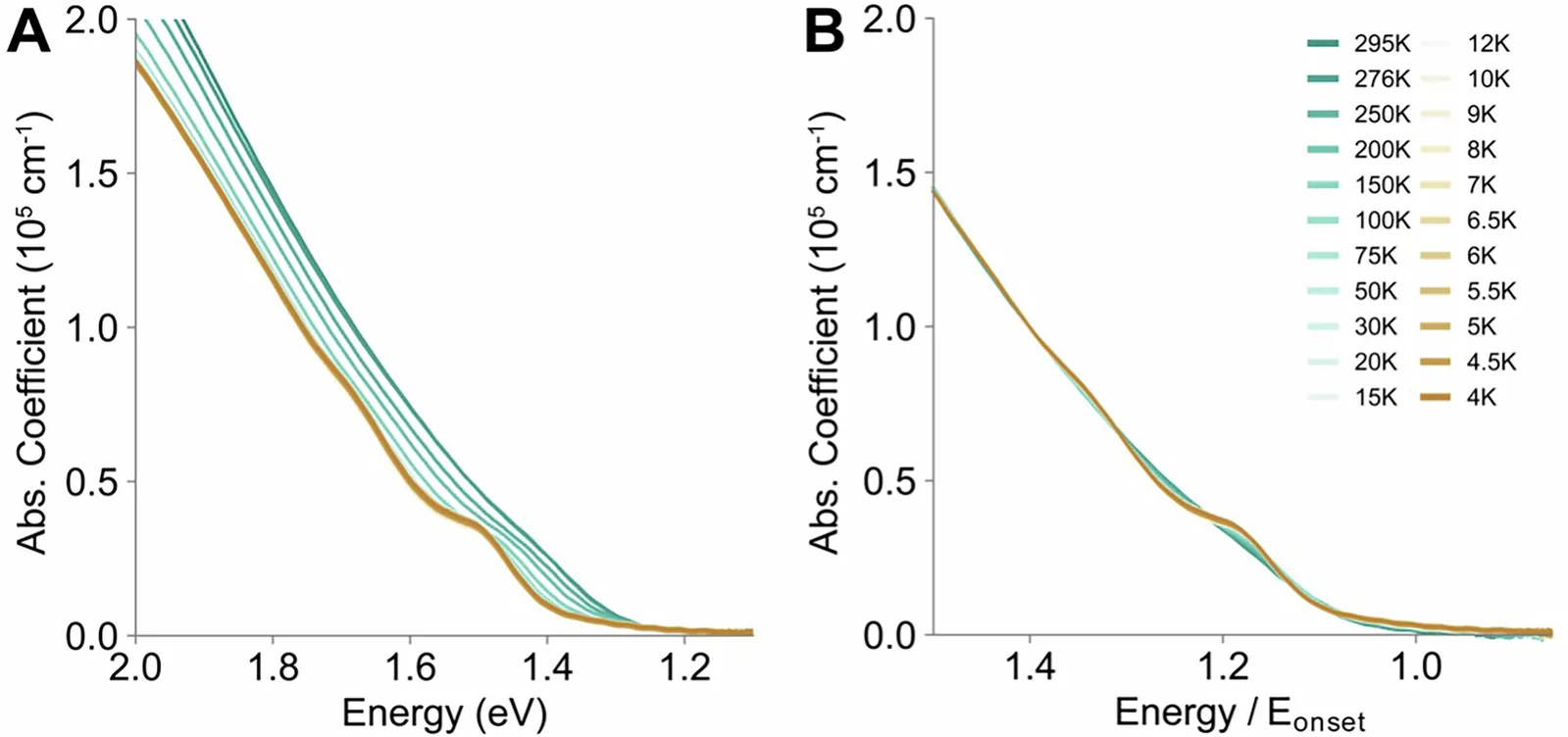
Temperature-dependent absorption. (**A**) Absorption coefficient for Bi_2_S_3_ thin films on quartz, measured in the NIR-Vis range for various temperatures between 4 K and 295 K. Selected curves from this dataset are presented in the main text (Fig. 1c). (**B**) For representation purposes, the energy axis (x-axis) of absorption coefficients measured at different temperatures (sub-figure A) was redefined to allow the onset energies (*E*_onset_) to coincide as an *E*/*E*_onset_ axis, in order to highlight potential temperature-induced changes. This representation clearly highlights that the strength of the absorption (i.e., the magnitude of the absorption coefficient) does not change with temperature.

Despite a high absorption coefficient (*α* > 10^5^ cm^−1^ for visible light), the absorption spectrum for Bi_2_S_3_ thin films at room temperature (Fig. 1c) lacks a sharp onset, similar to what is observed for AgBiS_2_^38^, NaBiS_2_^39^ and other bismuth-based semiconductors^40,41^. Although this gradual absorption onset resembles that of a prototypical indirect semiconductor (scaling as *E*^2^) more than that assumed for a direct semiconductor (scaling as *E*^0.5^, assuming parabolic bands), the values of the absorption coefficient are too high to be compatible with an indirect absorption process^35^. Through our first-principles calculations of Bi_2_S_3_, we show how the computed optical absorption spectrum (see the Supplementary Information for details) shown in Fig. 1d, which includes only contributions from direct transitions, exhibits a gradual onset with a similar line shape to that of the measured absorption coefficient. This comparison suggests that direct transitions form substantive components of the measured absorption. Our temperature-dependent absorption measurements (Fig. 1c) further rule out an indirect nature of these absorption-coefficient components governing the dominant onset: the magnitude of the absorption coefficient above the dominant onset remains nearly constant at cryogenic temperatures (Extended Data Fig. 2) and is, therefore, not compatible with being governed by phonon-assisted indirect transitions.

Despite the direct nature of the optical absorption features associated with the dominant onset, the electronic band structure calculated at the DFT/PBE + SOC level for Bi_2_S_3_ indicates that the lowest-energy transition is indirect. Furthermore, by comparing with DFT calculations using the global hybrid functional PBE0^42^, and the *GW* approximation^43^, we find that the pseudo-direct character of the band gap is maintained, regardless of the level of theory employed in our calculations (see Supplementary Table 3). To finely resolve this feature, we computed the DFT/PBE band edges between the Γ, X, Z and U points of the Brillouin zone (Fig. 1e,f), found at *k*_*x*_, *k*_*y*_, *k*_*z*_ coordinates (0,0,0), (1/2, 0, 0), (0, 0, 1/2) and (1/2, 0, 1/2), in units of the reciprocal lattice. The conduction band minimum (Fig. 1e) is found along the Γ–Z direction at (0,0, 1/6) and is located near the lowest-energy direct transition at (0,0, 1/5) (Extended Data Fig. 1b). However, the valence band maximum (Fig. 1f) is found at (1/3, 0, 0) and at (1/3, 0, 1/12), thus confirming the indirect nature of the lowest-energy transition in Bi_2_S_3_. We find this indirect bandgap to be red-shifted from the lowest-energy direct transition by less than 40 meV (see Supplementary Table 3), which is consistent with previous DFT calculations^32,44^. Although this weak indirect transition does not contribute appreciably to the measured Bi_2_S_3_ absorption spectrum, we note that it could play an important part in charge-carrier recombination processes in Bi_2_S_3_ given that injected charge carriers rapidly relax to form a thermal equilibrium near the band edges.

## Delayed recombination in Bi_2_S_3_ thin films

**Fig. 2:**
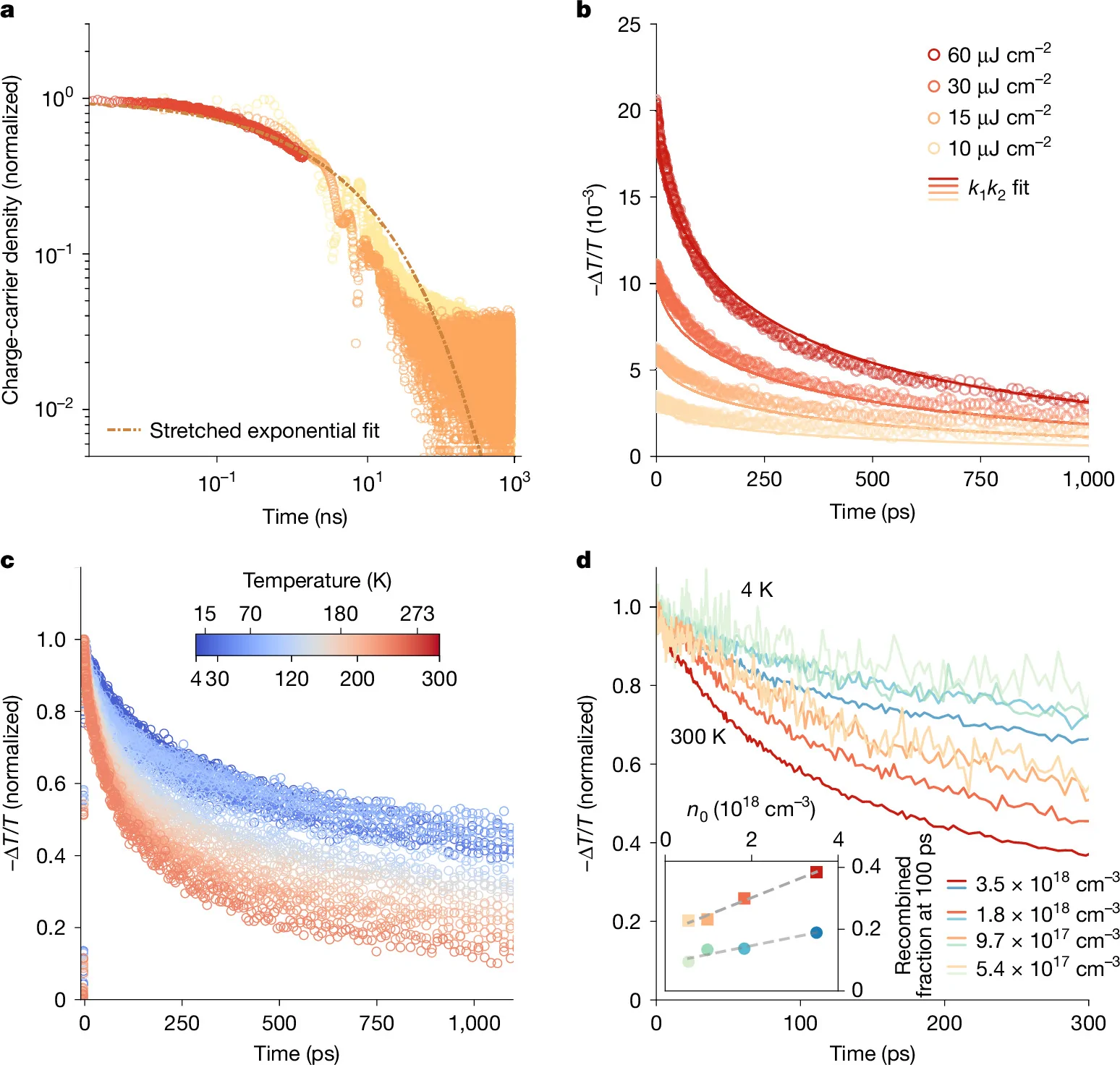
Charge-carrier dynamics in Bi_2_S_3_ thin films following pulsed photoexcitation. **a**, Room-temperature charge-carrier decay dynamics in Bi_2_S_3_ thin films, represented by the decay of THz photoconductivity (open red circles), mmW conductivity transients (yellow open circles), and by the photoluminescence decay (square root of intensity, shown as orange open circles). Photoconductivity and photoluminescence data were recorded following 1.55-eV, 35-fs pulsed excitation with a fluence of 60 µJ cm^−2^. The brown dash-dot line represents the fit to the conductivity data resulting from a stretched exponential model (Supplementary Information). **b**, Fluence-dependent THz photoconductivity transients represented as fractional change in the transmitted THz electric field (−Δ*T*/*T*) for Bi_2_S_3_ thin films, measured after 1.55-eV, 35-fs pulsed excitation with a range of different excitation fluences at room temperature (*T* = 300 K). Open circles are experimental data, and solid lines represent global fits to the *k*_1_*k*_2_ model described in the main text (see Supplementary Information for further details). **c**, Temperature-dependent normalized THz photoconductivity for Bi_2_S_3_ thin films measured between 4 K and 295 K following 1.55-eV, 35-fs pulsed photoexcitation with fluence 75 µJ cm^−2^. Data recorded for different fluences are reported in the Supplementary Information. **d**, Temperature evolution of the fluence-dependent THz photoconductivity. Comparison of fluence-dependent THz photoconductivity transients measured at 300 K (red-shaded coloured lines) and at 4 K (blue-shaded coloured lines). The inset shows the fraction of recombined charge carriers after 100 ps at 300 K and 4 K (squares for 300 K and circles for 4 K) as a function of the initial charge-carrier density. The grey dashed lines are guides to the eye.

**Extended Data Fig. 3:**
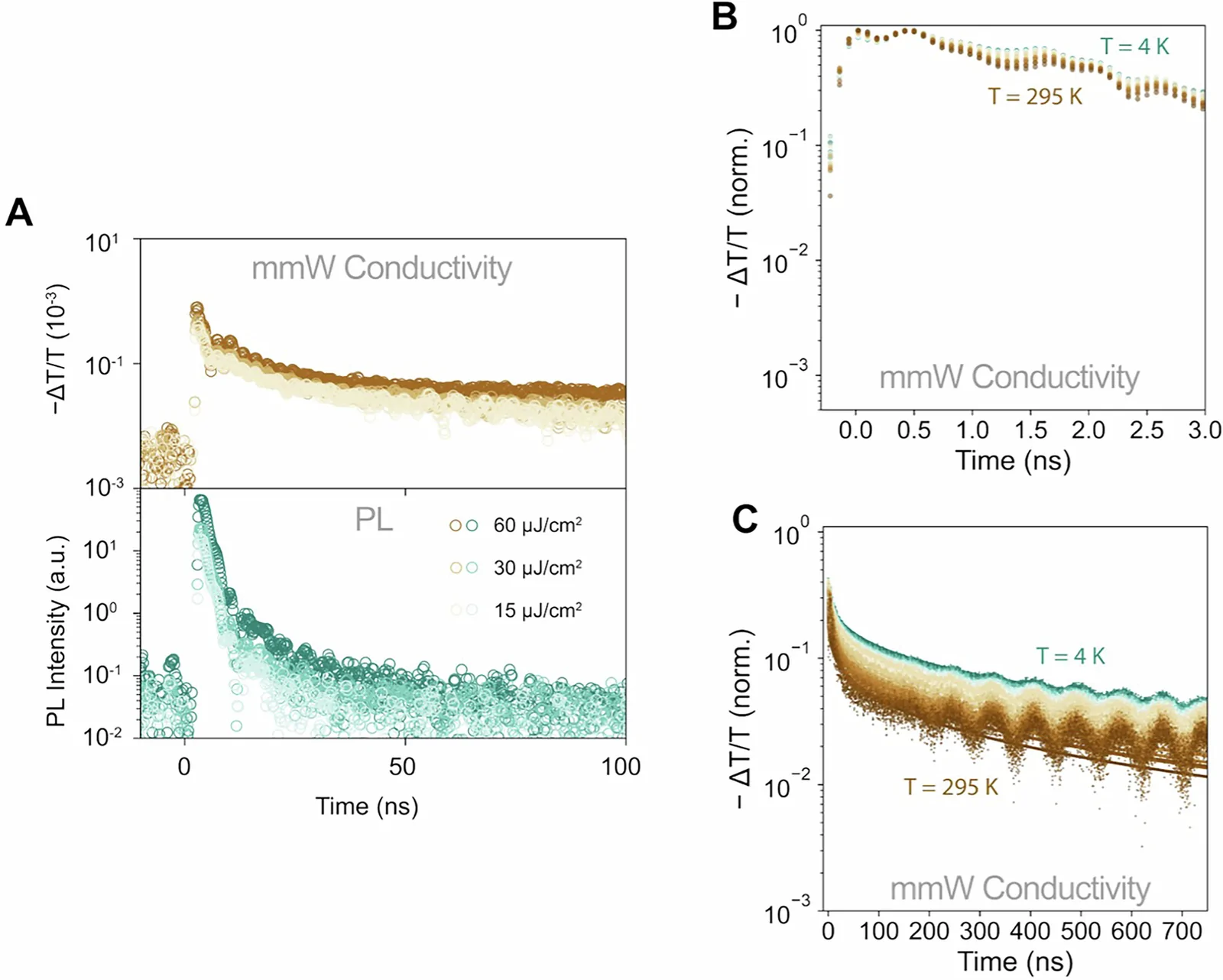
ns-to-µs charge-carrier dynamics and recombination. (**A**) Time-resolved millimetre-wave (mmW) conductivity (upper panel, yellow) and photoluminescence (lower panel, green) transients measured for Bi_2_S_3_ thin films on quartz following 1.55-eV, 35-fs pulsed excitation with different fluences indicated by different shades of yellow and green colour as shown in the legend. (**B**) Time-resolved mmW conductivity measured at early times for Bi_2_S_3_ thin films on quartz at different temperatures in the range 4 to 295 K following 1.55-eV, 35-fs pulsed excitation with a fluence of 60 µJ cm^−2^. The presence of a non-negligible photoconductivity decay in the time window probed by OPTP (~1 ns timescale) is caused by the dispersive (e.g., stretched exponential) nature of the decay. (**C**) Time-resolved mmW conductivity for Bi_2_S_3_ thin films on quartz at different temperatures in the range 4 to 295 K following 1.55-eV 35-fs pulsed excitation with a fluence of 60 µJ cm^−2^. Coloured dots represent experimental photoconductivity data; solid coloured lines represent fits to a stretched exponential decay model with a β ~ 0.5. The agreement with the stretched exponential model indicates the dispersive nature of trap-mediated recombination of charge carriers in Bi_2_S_3_.

To examine the implications of the peculiar band structure of Bi_2_S_3_, we investigated the dynamics of photogenerated charge carriers in Bi_2_S_3_ thin films by combining a range of complementary experiments, including THz conductivity, millimetre-wave (mmW) conductivity^25^, and photoluminescence (PL) probes. Figure 2a shows the derived normalized charge-carrier density transients for Bi_2_S_3_ thin films following 1.55-eV photoexcitation (60 µJ cm^−2^ fluence) over a broad time range (from 200 fs to 1 µs) plotted on a logarithmic time scale (see Extended Data Fig. 3a and Supplementary Information). On the ns-timescale, we observe that charge carriers almost entirely recombine within about 100 ns, and the fluence-independent decay is well described by a stretched exponential model (Extended Data Fig. 3b,c), which is compatible with previously reported defect-mediated recombination in Bi_2_S_3_ (ref. ^34^) and reveals the dispersive nature of trapping processes in Bi_2_S_3_ thin films. At higher excitation fluences and shorter (ps) timescales, charge-carrier dynamics in Bi_2_S_3_ reveal the fluence-dependent behaviour characteristic of intrinsic bimolecular recombination, with faster recombination dynamics observed at higher fluence (Fig. 2b). We modelled the charge-carrier dynamics through a *k*_1_*k*_2_ model, which can separate contributions from monomolecular (for example, defect-mediated, extrinsic) and bimolecular intrinsic (electron–hole band-to-band) recombination processes (see detailed description in Supplementary Note 1). Importantly, at longer time delays (Fig. 2a), the monomolecular recombination has a highly dispersive character (that is, slows down at longer time delays) and is well described by a stretched exponential decay. To account for this, we implemented a Kohlrausch–Williams–Watts (KWW) monomolecular rate (Supplementary Note 1) in our *k*_1_*k*_2_ model. By globally fitting this model to the fluence-dependent THz conductivity, we extract a bimolecular recombination coefficient *k*_2_ ≈ 5 × 10^−11^ cm^3^ s^−1^ at room temperature. Bi_2_S_3_ shows a lower *k*_2_ rate constant than those of direct semiconductors such as GaAs and lead halide perovskites^24,45,46^, despite exhibiting a substantially higher absorption coefficient (*α* ≈ 10^5^ cm^−1^), thus indicating a suppression of *k*_2_ according to the vRS theory. Nevertheless, the obtained *k*_2_ value strongly indicates that bimolecular recombination in Bi_2_S_3_ ultimately proceeds through an optically allowed transition with direct character, as purely indirect transitions are expected to yield orders-of-magnitude lower *k*_2_ values^17,18^. Given that the calculated indirect gap is energetically separated from the higher-lying direct gap by only about 40 meV, at room temperature, this behaviour may ultimately derive from a thermal population of charge carriers being shared between several bands.

**Extended Data Fig. 6:**
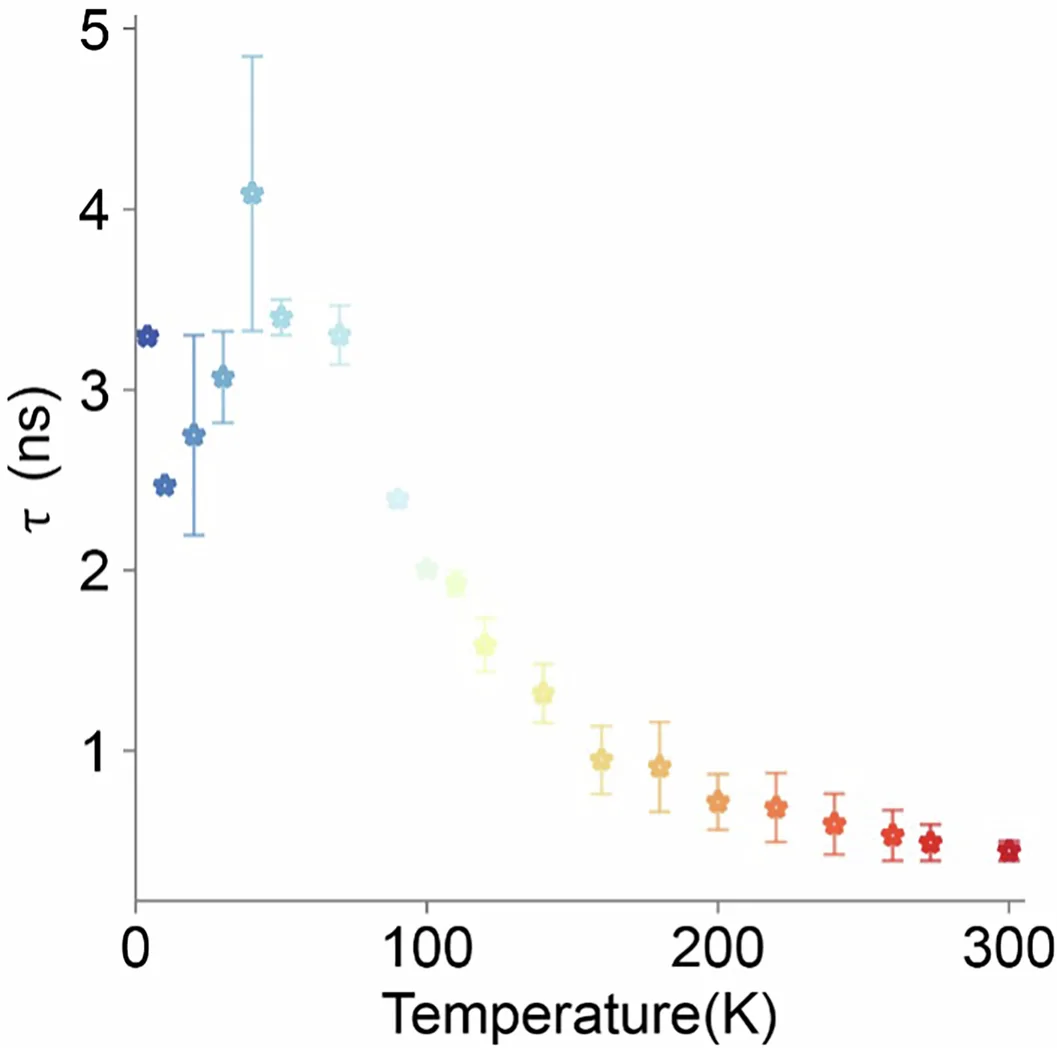
Temperature dependence of the monomolecular characteristic decay time. KWW monomolecular recombination characteristic time as a function of temperature. Together with *k*_2_ values (Fig. 3d), these characteristic times were obtained from *k*_1_*k*_2_ fits of the temperature-dependent OPTP transients measured for Bi_2_S_3_ thin films following 1.55-eV, 35-fs pulsed photoexcitation (Extended Data Figs. 4, 5).

To explore the interplay between the closely spaced indirect and direct transitions in Bi_2_S_3_, we investigated the temperature dependence of charge-carrier dynamics in Bi_2_S_3_ thin films. As shown in Fig. 2c, optical-pump THz-probe (OPTP) photoconductivity transients show markedly slower charge-carrier recombination at cryogenic temperatures than at room temperature. This trend is notable because, for intrinsic bimolecular recombination, the opposite behaviour—that is, faster electron–hole recombination at low temperatures—is generally observed in both direct^24,25,47^ and indirect semiconductors^17,18^. However, we note that temperature influences both monomolecular (trap-mediated) and bimolecular (electron–hole) recombination processes. On the one hand, the Shockley–Read–Hall model of trap-mediated recombination predicts a reduction of monomolecular recombination rates at low temperatures as a result of hindered thermal capture of charge carriers^48^, which is also observed here for Bi_2_S_3_ (Extended Data Fig. 6). On the other hand, the vRS theory predicts faster bimolecular recombination processes at low temperatures^3,4^ related to the sharpening of the Fermi–Dirac distribution function. To qualitatively disentangle these effects, we compare the fluence-dependent THz photoconductivity decays measured at 300 K and 4 K (Fig. 2d). These data may separate the two effects, given that defect-mediated extrinsic (*k*_1_) recombination is generally invariant with increasing injected charge-carrier density, whereas bimolecular intrinsic (*k*_2_) recombination processes accelerate markedly. Therefore, the extent of the fluence dependence (that is, how much transients ‘fan out’ across fluences) is a qualitative measure of the *k*_2_ contribution. Figure 2d shows a reduced fluence dependence at 4 K with respect to 300 K, thereby indicating an unusual reduction in the bimolecular recombination channel at lower temperatures. This observation is further confirmed by the increased dependence on the initial charge-carrier density of the fraction of charge carriers that have recombined within 100 ps (Fig. 2d, inset) at room temperature, compared with 4 K. As we demonstrate further below through quantitative analysis across a wide temperature range, this behaviour is indicative of a retardation in intrinsic bimolecular recombination rates at low temperature, an effect that is not usually seen in direct or indirect semiconductors.

**Fig. 3:**
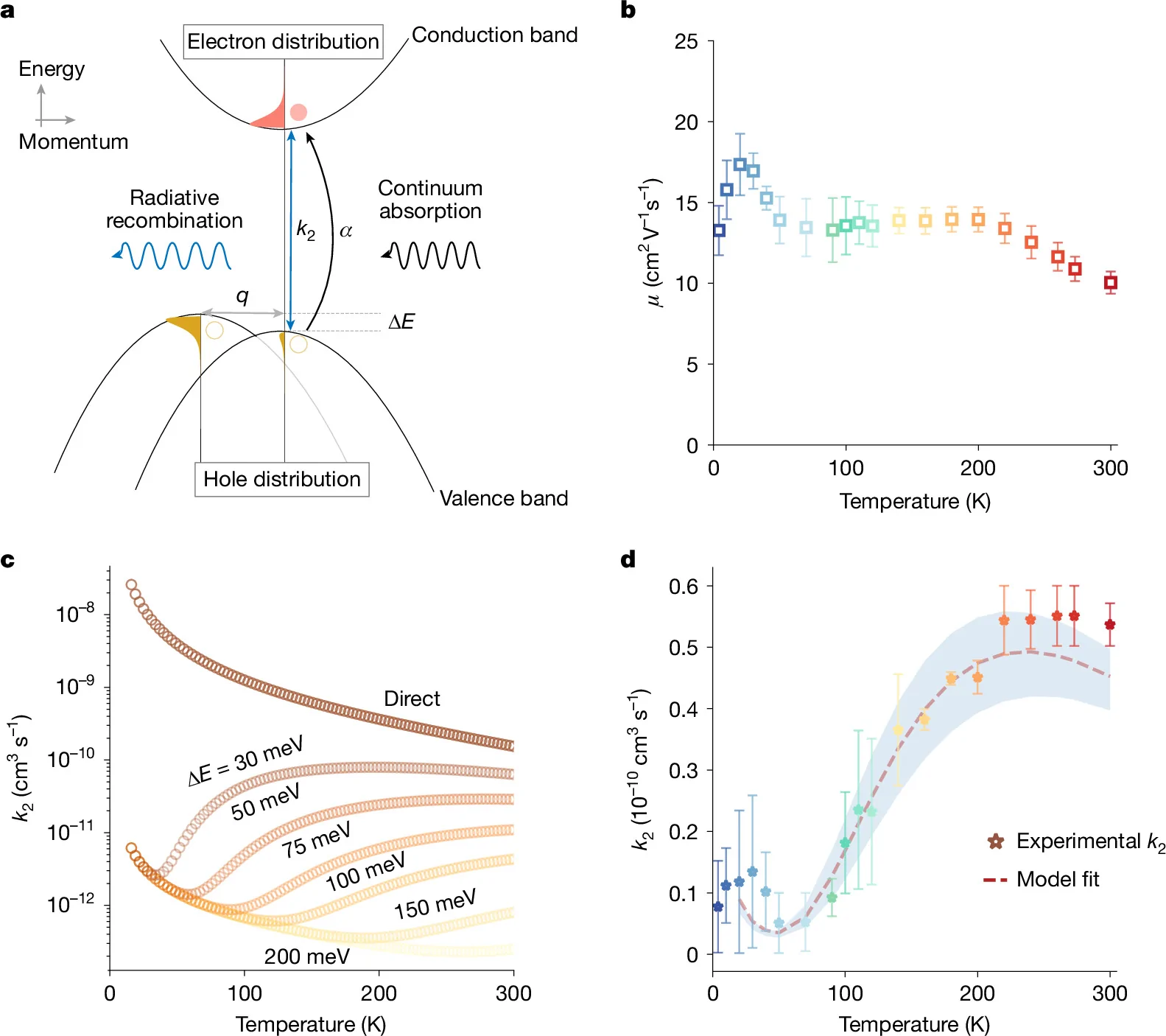
Temperature-dependent charge-carrier recombination dynamics in Bi_2_S_3_ thin films. **a**, Schematic of the energy–momentum dispersion relation and charge-carrier distributions in a model mixed direct–indirect semiconductor. The combination of dispersion relations and distribution functions has a determining role in the bimolecular charge-carrier recombination process, described by the vRS relation. The pink- and gold-filled areas show the Fermi–Dirac distributions of the excited electrons and holes in the conduction and valence bands, respectively. Although the model assumes two valence bands spaced in energy by Δ*E* and momentum by *q*, we note that analogous results are obtained in the case of two conduction bands. **b**, Temperature dependence of effective THz electron–hole sum mobilities for Bi_2_S_3_ thin films extracted from the analysis of photoconductivity transients presented in the Supplementary Information. Data points are the mean of the values extracted from two independent temperature series measured in different fluence ranges (35–75 µJ cm^−2^, Extended Data Fig. 4; 10–60 µJ cm^−2^, Extended Data Fig. 5); error bars represent the standard error on the mean. **c**, Calculated temperature dependence of the bimolecular recombination coefficient *k*_2_ evaluated by applying the vRS relation to the model presented in Fig. 3a. Open circles with different colours represent calculated *k*_2_ values for different Δ*E* values between 30 meV and 200 meV. The upper end of this range is included to illustrate the convergence of pseudo-direct behaviour towards the conventional indirect limit, rather than to indicate favourable values for a pseudo-direct semiconductor (Extended Data Fig. 7b and Supplementary Note 3). Brown open circles represent the case of a direct semiconductor. **d**, Temperature dependence of bimolecular recombination coefficient *k*_2_ extracted from fluence-dependent OPTP measurements (Extended Data Figs. 4 and 5) on Bi_2_S_3_ thin film through the *k*_1_*k*_2_ model described in detail in Supplementary Note 1. The dashed line represents a best-parameter fit to the model shown in **c** and described in Supplementary Note 3. Data points are the mean of the values obtained from global fits to two independent temperature series measured in different fluence ranges (35–75 µJ cm^−2^, Extended Data Fig. 4; 10–60 µJ cm^−2^, Extended Data Fig. 5); error bars represent the standard error on the mean. The blue-shaded areas represent the two confidence intervals (95%) for the fitted parameters.

Owing to the intrinsic nature of bimolecular recombination processes, we posit that the peculiar temperature dependence of *k*_2_ originates from the structure of the Bi_2_S_3_ band edges. As shown in Fig. 3a, the electronic band structure (Fig. 1b) implies that the bimolecular recombination mechanism will be regulated by the thermal equilibrium between an indirect transition and a direct transition lying above by an energy Δ*E*, which is comparable to thermal energies *k*_B_*T* at room temperature. Therefore, at ambient temperatures, thermal broadening of the distribution function across several bands partially unlocks a fast direct bimolecular recombination pathway, thus explaining the direct-like *k*_2_ value obtained for Bi_2_S_3_. However, at low temperatures, a reduction in the thermal energy and the accompanying narrowing of the Fermi–Dirac distribution cause charge carriers to be condensed in the lower-lying band, thus yielding less efficient recombination of an indirect character. The unusual temperature dependence of the charge-carrier mobility in Bi_2_S_3_ thin films (Fig. 3b) further supports the presence of several bands contributing to charge-carrier transport, as discussed in Supplementary Note 4.

**Extended Data Fig. 7:**
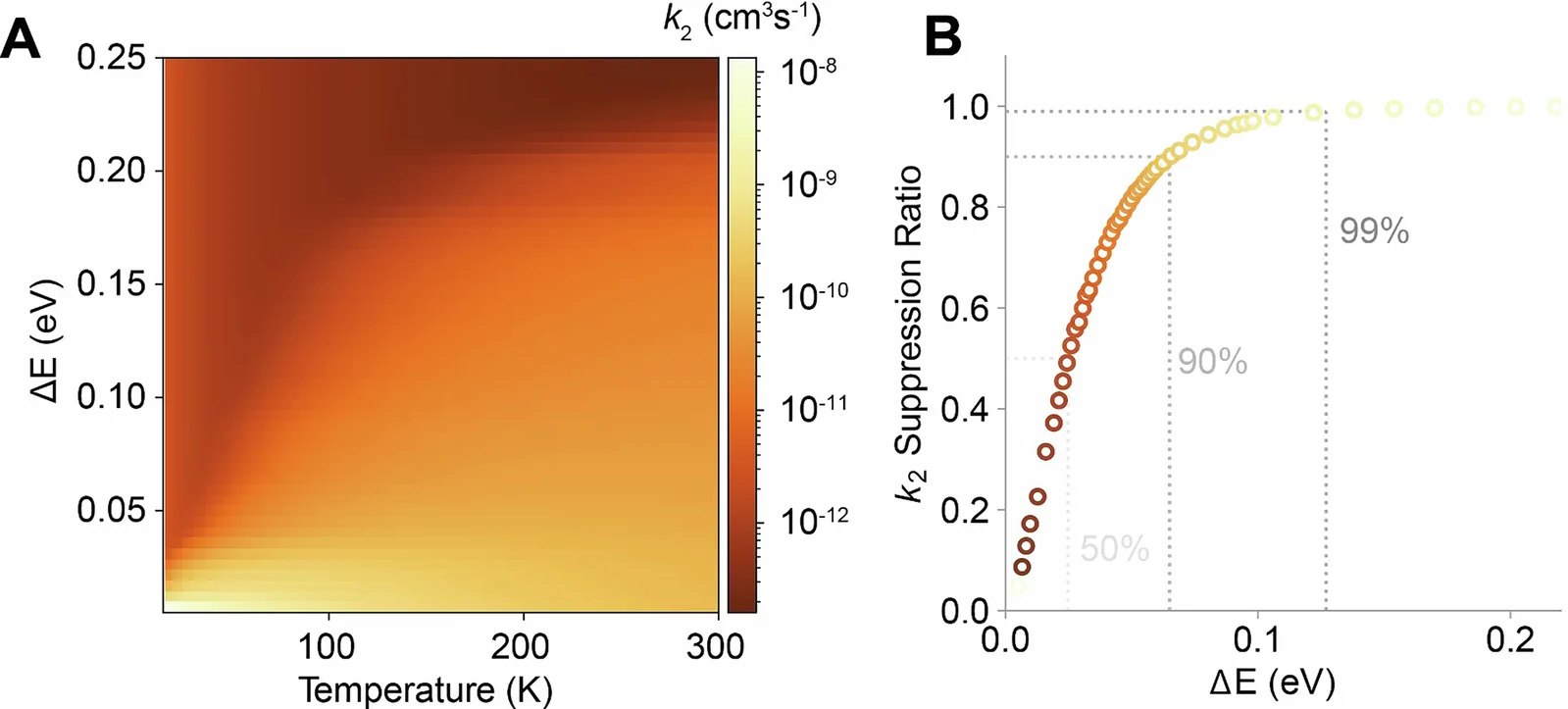
Calculated *k*_2_ suppression ratio at room temperature. (**A**) Calculated bimolecular recombination rate (*k*_2_) for a pseudo-direct semiconductor – see Supplementary Note 3 for details – plotted as a function of temperature *T* and direct-indirect gap splitting ΔE. (**B**) Calculated *k*_2_ suppression ratio for a pseudo-direct semiconductor plotted as a function of direct-indirect gap splitting ΔE, calculated at temperature *T* = 300 K. The *k*_2_ suppression ratio represents the fraction of suppressed bimolecular recombination, as a result of the pseudo-direct bandgap splitting. Grey dotted lines represent 50, 90, and 99% suppression ratio values, found at $\Delta E=\mathrm{24,\; 65,\; 127}\,{\rm{meV}}$, respectively. We note that for larger ΔE values, the calculated suppression saturates, while the range of the indirect absorption character widens, thus resulting in a material progressively reverting to a conventional indirect behaviour (see Fig. 3c).

To demonstrate the implications of the peculiar direct–indirect bandgap character for charge-carrier recombination in Bi_2_S_3_, we modelled the effective recombination rate constant based on the generalized Planck’s law^2,49^ by applying the vRS theory^3^ to the individual scenarios of direct, indirect and mixed direct–indirect bandgap character (Supplementary Note 3). As shown in Fig. 3c, the calculated bimolecular recombination coefficient *k*_2_ for a direct semiconductor increases with decreasing temperature, as expected. For example, a previous study experimentally verified this prediction for methylammonium lead iodide^4^, directly linking such an increase in *k*_2_ to the narrowing of the photon distribution function and the thermal spread of charge carriers across the band edges. For the intermediate case of a mixed indirect–direct semiconductor, for which an indirect transition with an energy difference Δ*E* =* E*_direct_ −* E*_indirect_ lies closely below a direct transition, a substantially different temperature dependence is expected for *k*_2_. Our model shows that for Δ*E* on the order of a few *k*_B_*T*, *k*_2_ should exhibit an initial reduction towards lower temperatures, followed by a moderate increase (Fig. 3c). This trend is explained by the interplay between the narrowing of the Fermi–Dirac distribution functions in the bands and the reduced thermal population of the band involved in the direct transition. As temperature is reduced from room temperature, more charge carriers occupy bands associated with the indirect transition, leading to a suppression of bimolecular recombination. However, at the lowest temperatures, electrons and holes will become mostly located within one respective band, meaning that any further narrowing of the Fermi–Dirac distribution leads to increases in *k*_2_, as captured by the model (Fig. 3c). The experimentally determined temperature dependence of *k*_2_ that we reveal here from OPTP measurements on Bi_2_S_3_ thin film (Fig. 3d) confirms the qualitative trend observed in Fig. 2d and closely follows the modelled recombination for a mixed direct–indirect bandgap semiconductor with an energy splitting Δ*E*. To further support this, we fit the experimental temperature-dependent *k*_2_ values to our recombination models and find that the observed *k*_2_ dependence is compatible with a Δ*E *≈ 38 meV, similar to the value of about 40 meV determined from the first-principles calculations (see Supplementary Table 3). As Extended Data Fig. 7b shows, calculations based on our model (Supplementary Note 3) show that this energy difference Δ*E* yields a substantial suppression of the bimolecular recombination rate constant by approximately 75% at room temperature, with respect to the case of a purely direct semiconductor.

## Discussion

The unique charge-carrier recombination mechanism in Bi_2_S_3_ thin films shows the potential of exploiting multiple, closely spaced electronic bands to favourably tune intrinsic recombination processes in a semiconductor. The absorption coefficient of Bi_2_S_3_ is dominated by direct, strong interband transitions (*α* > 10^5^ cm^−1^) arising within tens of meV of the band edges, making this a promising solar absorber. However, within tens of meV below the band edges associated with direct transitions lies an indirect transition, which slows down intrinsic bimolecular recombination processes in this material appreciably. The combination of our temperature-dependent absorption and temperature- and fluence-dependent OPTP results, together with vRS modelling (Fig. 3c and Extended Data Fig. 7) demonstrates that this configuration suppresses the bimolecular recombination rates in Bi_2_S_3_ by about 75% at room temperature as calculated for Δ*E* = 40 meV. The vRS relation builds on the identity between the absorber and emitter states, and accounts for the well-known behaviour of direct and indirect semiconductors: direct transitions yield high absorption coefficients and fast recombination, whereas indirect transitions are associated with low absorption coefficients and slow intrinsic bimolecular recombination processes. In Bi_2_S_3_, the small energy difference (comparable to *k*_B_*T*) between direct and indirect transitions at the band edges, computed from first principles (Supplementary Table 3) as Δ*E* ≈ 40 meV, effectively decouples light absorption near the band edge and charge-carrier recombination processes. Within such a configuration, although recombination is still dominated by the direct recombination process owing to its substantially higher rate, charge carriers spend a considerable amount of time in a transient ‘reservoir’ formed by states associated with the indirect gap, thereby slowing the overall recombination process. This decoupling circumvents this reciprocity between *α* and *k*_2_ without compromising charge-carrier transport and blends the advantageous properties of direct and indirect semiconductors, a long-sought goal in the field of solar absorbers for photovoltaics and solar fuels.

Therefore, more generally, this interplay between direct and indirect transitions in Bi_2_S_3_ defines a new type of pseudo-direct semiconductor that defies the conventional dichotomy between direct and indirect semiconductors. As our simulations in Extended Data Fig. 7 show, charge-carrier recombination lifetimes in such pseudo-direct semiconductors can potentially be extended by up to two orders of magnitude (see higher *k*_2_ suppression ratios in Extended Data Fig. 7) at the sacrifice of small Δ*E *≈ 100 meV energy loss resulting from the presence of a lower-lying indirect transition. In the Shockley–Queisser theoretical framework, the optical reciprocity principle underpins emission losses^50^, and for this reason, nonreciprocal absorbers were proposed to yield considerable conversion efficiency gains in multijunction solar cells^50,51^. Therefore, we note that circumventing this reciprocity between *α* and *k*_2_ offers a promising strategy to potentially enhance solar energy conversion efficiencies, and to generally extend charge-carrier diffusion lengths (see Supplementary Note 5 for a more detailed discussion). Beyond conversion efficiency gains in sunlight harvesting, modulating the charge-carrier recombination lifetime in highly absorbing semiconductors also has the potential to unlock new photocatalytic applications in which longer timescales (for example, multi-electron transfer reactions, reactant turnover times) are involved.

**Extended Data Fig. 8:**
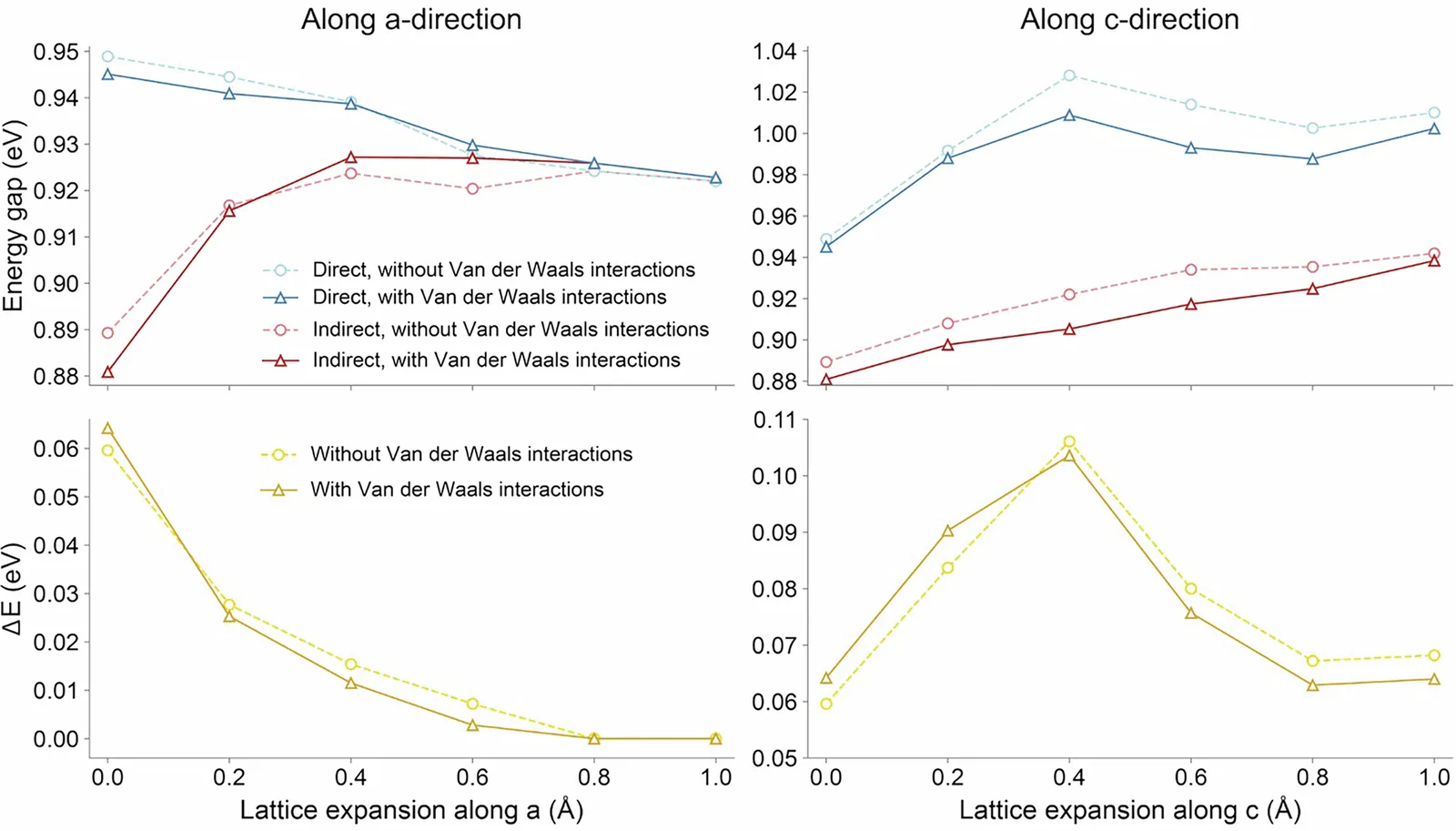
Calculated tuning of direct–indirect splitting through lattice expansion. Direct (lines with open blue symbols) and indirect (lines with open red symbols) band gaps calculated for structures with increasing inter-ribbon distance along the a and c directions. Lower panels show the corresponding energy difference between these transitions (i.e., the ΔE value). Circles and dashed lines indicate structures relaxed without van der Waals corrections, whereas triangles and solid lines denote structures relaxed including van der Waals interactions, starting from structures with extended and fixed a and c lattice parameters, and shifted ribbons (ensuring the ribbons are undistorted). The direct and indirect band gaps are identified from calculations of energy eigenvalues over a Γ-centered k-point grid of 9x24x9.

Our modelling (Fig. 3c and Extended Data Fig. 7) suggests that either identifying new pseudo-direct semiconductors with targeted Δ*E* values or implementing approaches to directly control Δ*E* will be crucial areas of development. We foresee a strategy to discover possible pseudo-direct candidates that builds on a two-phase methodology: (1) an initial coarser screening of large material databases—for example, the Materials Project^52^—to identify candidate semiconductors and insulators showing Δ*E *≈ 2–3 *k*_B_*T* ; and (2) a refinement of the candidate pool based on their band gap magnitude calculated using hybrid functionals, and further sifting through pseudo-direct candidates for other desirable traits, such as high absorption coefficients, low and isotropic effective masses, and favourable defect chemistries. A second strategy is to control and tune Δ*E* values directly through external stimuli, for instance, in materials with van der Waals nature of atomic bonding, such as in Bi_2_S_3_, and similarly transition metal dichalcogenides^53,54^, indium selenide^55,56^ and phosphorene^57^, whose direct–indirect splitting, for example, Δ*E *≈ 60 meV for WSe_2_ and Δ*E *≈ 50 meV for InSe could potentially yield *k*_2_ suppression by 88% and 81%, respectively, according to our model. In these materials, inter-ribbon (or interlayer) interactions may be further exploited to tune Δ*E*. For example, our calculations (Extended Data Fig. 8) show that when increasing the distance between (Bi_4_S_6_)_*n*_ ribbons along the *a*-axis and *c*-axis of the Bi_2_S_3_ crystal structure (that is, when expanding the lattice), Δ*E* can be increased up to around 100 meV. We note that in such van-der-Waals-bonded, layered materials, interlayer interactions may be tuned through exfoliation^58^, strain^32^ and intercalation^59^ strategies, with the potential to directly control intrinsic bimolecular recombination processes in pseudo-direct semiconductors. In conclusion, our findings reveal the unique nature of charge-carrier recombination in Bi_2_S_3_, which embodies the features of pseudo-direct semiconductors, a concept that will not only set a new benchmark in materials discovery but also offer prospective opportunities for substantial advances in solar energy conversion applications.

## Methods

### Bi_2_S_3_ thin film preparation

Bi_2_S_3_ thin films were fabricated following a previously published thermal evaporation method on ST-cut quartz substrates^30^. Briefly, the thin films were deposited in a thermal evaporator. In the evaporation chamber, the vacuum pressure before evaporation was set to about 3 × 10^−5^ Torr, and the substrate temperature was maintained at room temperature. The thickness of the Bi_2_S_3_ film was monitored during deposition and was determined to be about 150 nm by using a profilometer. The as-deposited samples were then annealed for 30 min in an N_2_-filled glove box at 250 °C.

### Absorption measurements

Absorption spectra for Bi_2_S_3_ thin films were measured using a Fourier-transform infrared spectrometer (Bruker-VERTEX 80v) fitted with a transmission–reflection accessory. Reflectance and transmittance spectra in the NIR region (0.5–1.2 eV) were taken using a tungsten halogen source and an InSb detector, and spectra in the visible region (1.2–2.5 eV) were taken using a tungsten halogen source and an Si detector. The obtained spectra were stitched by matching the absorbance curves at 1.2 eV. The absorption coefficient was obtained by dividing the absorbance spectra by the film thickness (about 150 nm) measured by a profilometer. For cryogenic absorption measurements, the samples were mounted in a gas-exchange helium cryostat (OptistatCF2, Oxford Instruments) and measured at different temperatures under a helium atmosphere. For temperature-dependent measurements, the chamber temperature was varied between 4 K and 295 K. The temperature was monitored using a sensor placed near the sample.

### X-ray diffraction

X-ray diffraction patterns were measured using a PANalytical X’Pert diffractometer, using Cu Kα_1_ radiation, across 2*θ* values ranging from 15° to 48°. The scan speed was 0.01° s^−1^, and the step size was 0.004°.

### DFT calculations

Ab initio calculations based on DFT were carried out for this work, as implemented in the plane-wave basis code Quantum Espresso^61,62^ using the Perdew–Burke–Ernzerhof exchange-correlation functional (PBE)^63^, including spin–orbit coupling (DFT/PBE + SOC). We used the standard norm-conserving fully relativistic Vanderbilt pseudopotentials^64^ from the PseudoDojo database^65^. Calculations were performed with a kinetic-energy cut-off of 75 Ry, with a uniform, Γ-centred *k*-point grid^66^ of (4 × 6 × 4). We used the experimental Bi_2_S_3_ structure, as reported in ref. ^67^ in all calculations reported in this study, with the exception of calculations shown in Extended Data Fig. 8, for which details are given below.

We computed the effective masses at the band extrema using the finite difference method, as implemented in the Effective Mass Calculator utility^68^. We used a 5-point stencil with a *k*-step size of 0.019 Å^−1^ to compute the second derivative of the energy with respect to the wave vector.

We performed hybrid functional calculations using the global PBE0 functional^42^, including spin–orbit coupling, to check the robustness of the band edge shape computed from PBE + SOC. We used an exchange cut-off energy of 100 Ry with a commensurate (4 × 6 × 4) *q*-point grid. With this setup, we obtained an indirect bandgap of approximately 2 eV and the lowest direct bandgap of about 2.04 eV (see Supplementary Table 3 for a detailed comparison). Furthermore, we checked the PBE0 correction of the direct band gap at each point of our 4 × 6 × 4 *k*-point grid, and found that the band gap is approximately rigidly shifted by an average value of 1.14 eV (with a standard deviation of about 60 meV). Further details regarding GW/BSE calculations are provided in the Supplementary Information.

Furthermore, we studied the impact of inter-ribbon interactions on the electronic structure by calculating band gaps as a function of the distance between the ribbons along the *a*- and *c*-directions. For these calculations, we increased the distance between the ribbons along the *a*- and *c*-directions separately, and relaxed the atomic positions. All band gaps computed for this analysis were calculated using semilocal DFT/PBE + SOC.

### Time-resolved photoluminescence and mmW conductivity

Time-resolved photoluminescence (TRPL) and mmW conductivity were simultaneously measured using a custom-built setup that has been described in detail before^25^. Briefly, microwave radiation at 34 GHz was generated using a signal generator and converted to mmW radiation at 102 GHz using a frequency tripler and launched into free space with a gain feed horn antenna. The high-frequency mmW radiation was focused onto the Bi_2_S_3_ thin film on a quartz substrate placed in a cold-finger cryostat and re-focused onto a mmW radiation detector by using dielectric lenses. A custom-made GaAs Schottky-diode detector was used to detect the mmW radiation field transmitted through the sample. Photo-induced changes to the transmitted mmW signal were amplified with a custom-built broad-band amplifier and recorded with a fast (4 GHz bandwidth) oscilloscope. TRPL was measured concurrently using a spectral filter (to remove laser scatter) and a fast photodiode detector (time resolution 3 ns, see ref. ^25^), from which the signal was amplified with a broad-band amplifier made in-house and measured with the same oscilloscope. Photoexcitation was provided by pulses of 35-fs duration originating from the fundamental output (1.55 eV) of an amplified Ti:sapphire laser system (Spectra-Physics, Spitfire ACE). For temperature-dependent TRPL measurements, the fundamental output was converted to 1.67 eV using an optical parametric amplifier (OPA, TOPAS-C, Light Conversion) to achieve a higher signal-to-noise ratio.

### OPTP spectroscopy

For OPTP measurements, we used a setup that has been described in detail previously^25,69^. Briefly, pulses of 1.55 eV photon energy, 35-fs duration and 5-kHz repetition rate are provided by an amplified Ti:sapphire laser system (Spectra-Physics, Spitfire ACE). This fundamental output was used to generate single-cycle THz radiation pulses in a spintronic emitter (2nm-W–1.8nm-Co_40_Fe_40_B_20_–2nm-Pt) through the inverse spin Hall effect^70,71^. Fractional changes in the THz transmission are measured by using free-space electro-optic sampling. The THz detection setup comprises a 1-mm-thick ZnTe (110) crystal, a Wollaston prism and a pair of balanced photodiodes. In the setup, samples are excited by a fraction of the fundamental output. We measured Bi_2_S_3_ thin films deposited onto 2-mm thick quartz, achieving a subpicosecond (about 200 fs) time resolution^69^. During OPTP measurements, the THz emission and detection setups and samples are kept under vacuum at pressures below 0.1 mbar. For the temperature-dependent measurements, samples were mounted in a cold-finger cryostat (MicrostatHe, Oxford Instruments) and cooled by liquid helium. The temperature was regulated via a temperature controller connected to a resistive heater.

## Online content

Any methods, additional references, Nature Portfolio reporting summaries, source data, extended data, supplementary information, acknowledgements, peer review information; details of author contributions and competing interests; and statements of data and code availability are available at https://doi.org/10.1038/s41586-026-11223-x.

## Supplementary information

Supplementary Information This file contains additional details of the computational methods, Supplementary Tables 1–4, Supplementary Notes 1–5, Supplementary Figs. 1–9 and Supplementary References.

Peer Review File

## Figures and Tables

**Extended Data Fig. 4:**
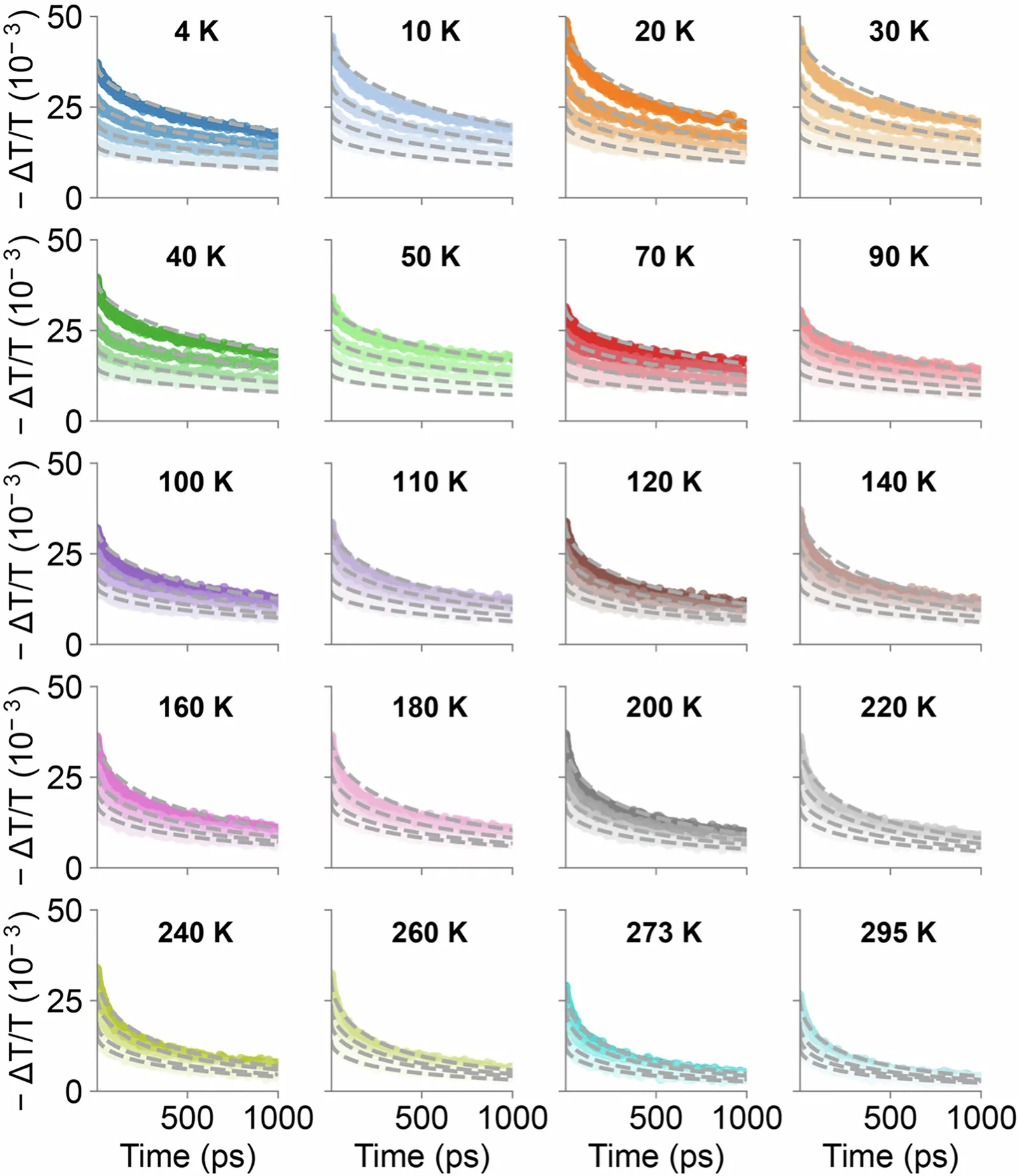
*k*_1_*k*_2_ fits to temperature-dependent OPTP transients in the higher fluence regime. Fluence-dependent OPTP transients measured for Bi_2_S_3_ thin films following 1.55-eV, 35-fs pulsed photoexcitation in the temperature range between 4 and 295 K. For each plot, the different shades of colour represent different fluences in the range 75 to 35 μJ cm^−2^. The grey dashed lines represent fits to the *k*_1_*k*_2_ model described in Supplementary Note 1.

**Extended Data Fig. 5:**
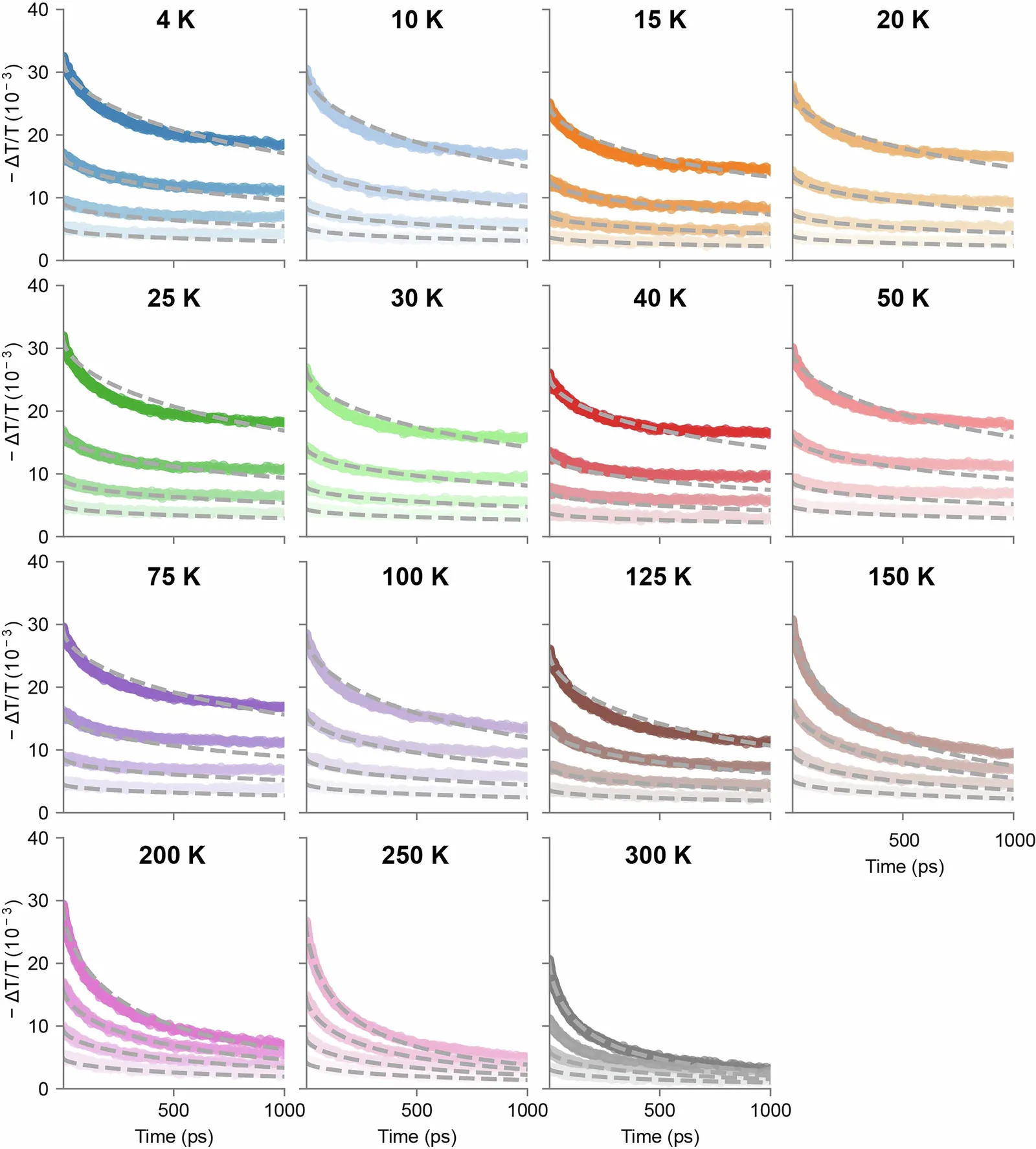
*k*_1_*k*_2_ fits to temperature-dependent OPTP transients of Bi_2_S_3_ in the lower fluence regime. Fluence-dependent OPTP transients measured for Bi_2_S_3_ thin films following 1.55-eV, 35-fs pulsed photoexcitation in the temperature range between 4 and 300 K. For each plot, the different shades of colour represent different fluences in the range 60 to 10 μJ cm^−2^. The grey dashed lines represent fits to the *k*_1_*k*_2_ model described in Supplementary Note 1.

## Data Availability

The datasets supporting the findings of this study are deposited in the Oxford University Research Archive (https://dx.doi.org/10.5287/ora-z6v7dgzra).

## References

[1] Kirchhoff, G. I. On the relation between the radiating and absorbing powers of different bodies for light and heat. *Lond. Edinb. Dubl. Philos. Mag. J. Sci.* **20**, 275–301 (1860).

[2] Würfel, P. The chemical potential of radiation. *J. Phys. C* **15**, 3967 (1982).

[3] van Roosbroeck, W. & Shockley, W. Photon-radiative recombination of electrons and holes in germanium. *Phys. Rev.* **94**, 1558–1560 (1954).

[4] Davies, C. L. et al. Bimolecular recombination in methylammonium lead triiodide perovskite is an inverse absorption process. *Nat. Commun.* **9**, 293 (2018). 10.1038/s41467-017-02670-2 PMC5773627 29348550

[5] deQuilettes, D. W. et al. Charge-carrier recombination in halide perovskites. *Chem. Rev.* **119**, 11007–11019 (2019). 10.1021/acs.chemrev.9b00169 31496228

[6] Krückemeier, L., Liu, Z., Kirchartz, T. & Rau, U. Quantifying charge extraction and recombination using the rise and decay of the transient photovoltage of perovskite solar cells. *Adv. Mater.* **35**, 2300872 (2023). 10.1002/adma.202300872 37147880

[7] Sum, T. C., Righetto, M. & Lim, S. S. Quo vadis, perovskite emitters? *J. Chem. Phys.* **152**, 130901 (2020). 10.1063/1.5132366 32268753

[8] Chen, Z. et al. Recombination dynamics study on nanostructured perovskite light-emitting devices. *Adv. Mater.* **30**, 1801370 (2018). 10.1002/adma.201801370 30088297

[9] Pastor, E. et al. Electronic defects in metal oxide photocatalysts. *Nat. Rev. Mater.* **7**, 503–521 (2022).

[10] Zachäus, C., Abdi, F. F., Peter, L. M. & van de Krol, R. Photocurrent of BiVO_4_ is limited by surface recombination, not surface catalysis. *Chem. Sci.* **8**, 3712–3719 (2017). 10.1039/c7sc00363c PMC5437485 28580106

[11] Johnston, M. B. & Herz, L. M. Hybrid perovskites for photovoltaics: charge-carrier recombination, diffusion, and radiative efficiencies. *Acc. Chem. Res.* **49**, 146–154 (2016). 10.1021/acs.accounts.5b00411 26653572

[12] Jin, H. et al. It’s a trap! On the nature of localised states and charge trapping in lead halide perovskites. *Mater. Horiz.* **7**, 397–410 (2020).

[13] Zhang, X., Turiansky, M. E., Shen, J.-X. & Van de Walle, C. G. Defect tolerance in halide perovskites: a first-principles perspective. *J. Appl. Phys.* **131**, 090901 (2022).

[14] National Laboratory of the Rockies (formerly NREL). Best research-cell efficiency chart. https://www.nlr.gov/pv/cell-efficiency (2026).

[15] Hossain, K., Sivadas, D., Kabra, D. & Nair, P. R. Perovskite solar cells dominated by bimolecular recombination─how far is the radiative limit? *ACS Energy Lett.* **9**, 2310–2317 (2024).

[16] Simbula, A. et al. Direct measurement of radiative decay rates in metal halide perovskites. *Energy Environ. Sci.* **15**, 1211–1221 (2022).

[17] Nguyen, H. T., Baker-Finch, S. C. & Macdonald, D. Temperature dependence of the radiative recombination coefficient in crystalline silicon from spectral photoluminescence. *Appl. Phys. Lett.* **104**, 112105 (2014).

[18] Trupke, T. et al. Temperature dependence of the radiative recombination coefficient of intrinsic crystalline silicon. *J. Appl. Phys.* **94**, 4930–4937 (2003).

[19] Milot, R. L., Eperon, G. E., Snaith, H. J., Johnston, M. B. & Herz, L. M. Temperature-dependent charge-carrier dynamics in CH_3_NH_3_PbI_3_ perovskite thin films. *Adv. Funct. Mater.* **25**, 6218–6227 (2015).

[20] Cowan, A. J. & Durrant, J. R. Long-lived charge separated states in nanostructured semiconductor photoelectrodes for the production of solar fuels. *Chem. Soc. Rev.* **42**, 2281–2293 (2013). 10.1039/c2cs35305a 23023269

[21] Buizza, L. R. V. & Herz, L. M. Polarons and charge localization in metal-halide semiconductors for photovoltaic and light-emitting devices. *Adv. Mater.* **33**, 2007057 (2021). 10.1002/adma.202007057 33955594

[22] Pastor, E. et al. In situ observation of picosecond polaron self-localisation in α-Fe_2_O_3_ photoelectrochemical cells. *Nat. Commun.* **10**, 3962 (2019). 10.1038/s41467-019-11767-9 PMC6722133 31481691

[23] Hutter, E. M. et al. Direct–indirect character of the bandgap in methylammonium lead iodide perovskite. *Nat. Mater.* **16**, 115–120 (2017). 10.1038/nmat4765 27698354

[24] Sarritzu, V. et al. Direct or indirect bandgap in hybrid lead halide perovskites? *Adv. Opt. Mater.* **6**, 1701254 (2018).

[25] Ulatowski, A. M. et al. Contrasting charge-carrier dynamics across key metal-halide perovskite compositions through in situ simultaneous probes. *Adv. Funct. Mater.* **33**, 2305283 (2023).

[26] Zhang, X., Shen, J.-X., Wang, W. & Van de Walle, C. G. First-principles analysis of radiative recombination in lead-halide perovskites. *ACS Energy Lett.* **3**, 2329–2334 (2018).

[27] Rath, A. K., Bernechea, M., Martinez, L. & Konstantatos, G. Solution-processed heterojunction solar cells based on p-type PbS quantum dots and n-type Bi_2_S_3_ nanocrystals. *Adv. Mater.* **23**, 3712–3717 (2011). 10.1002/adma.201101399 21732560

[28] Manna, G., Bose, R. & Pradhan, N. Photocatalytic Au–Bi_2_S_3_ heteronanostructures. *Angew. Chem. Int. Ed.* **53**, 6743–6746 (2014). 10.1002/anie.201402709 24844409

[29] Huo, N. et al. Engineering vacancies in Bi_2_S_3_ yielding sub-bandgap photoresponse and highly sensitive short-wave infrared photodetectors. *Adv. Opt. Mater.* **7**, 1900258 (2019).

[30] Yao, F. et al. Charge-carrier dynamics of evaporated bismuth-based chalcogenide thin films probed with time-resolved microwave conductivity. *J. Phys. Chem. Lett.* **14**, 5517–5523 (2023). 10.1021/acs.jpclett.3c00966 37290010

[31] Wang, X., Li, Z., Kavanagh, S. R., Ganose, A. M. & Walsh, A. Lone pair driven anisotropy in antimony chalcogenide semiconductors. *Phys. Chem. Chem. Phys.* **24**, 7195–7202 (2022). 10.1039/d1cp05373f 35262534

[32] Linhart, W. M., Zelewski, S. J., Scharoch, P., Dybała, F. & Kudrawiec, R. Nesting-like band gap in bismuth sulfide Bi_2_S_3_. *J. Mater. Chem. C* **9**, 13733–13738 (2021).

[33] Bernechea, M., Cao, Y. & Konstantatos, G. Size and bandgap tunability in Bi_2_S_3_ colloidal nanocrystals and its effect in solution processed solar cells. *J. Mater. Chem. A* **3**, 20642–20648 (2015).

[34] Aresti, M. et al. Colloidal Bi_2_S_3_ nanocrystals: quantum size effects and midgap states. *Adv. Funct. Mater.* **24**, 3341–3350 (2014).

[35] Yu, P. Y., Cardona, M. Optical properties I. in *Fundamentals of Semiconductors: Physics and Materials Properties* 4th edn., 243–344 (Springer, 2010).

[36] Hohenberg, P. & Kohn, W. Inhomogeneous electron gas. *Phys. Rev.* **136**, B864–B871 (1964).

[37] Kohn, W. & Sham, L. J. Self-consistent equations including exchange and correlation effects. *Phys. Rev.* **140**, A1133–A1138 (1965).

[38] Righetto, M. et al. Cation-disorder engineering promotes efficient charge-carrier transport in AgBiS_2_ nanocrystal films. *Adv. Mater.* **35**, 2305009 (2023). 10.1002/adma.202305009 37670455

[39] Huang, Y.-T. et al. Strong absorption and ultrafast localisation in NaBiS_2_ nanocrystals with slow charge-carrier recombination. *Nat. Commun.* **13**, 4960 (2022). 10.1038/s41467-022-32669-3 PMC9402705 36002464

[40] Lal, S. et al. Bandlike transport and charge-carrier dynamics in BiOI films. *J. Phys. Chem. Lett.* **14**, 6620–6629 (2023). 10.1021/acs.jpclett.3c01520 PMC10388347 37462354

[41] Farooq, S. et al. High gain solution-processed carbon-free BiSI chalcohalide thin film photodetectors. *Adv. Funct. Mater.* **31**, 2104788 (2021).

[42] Perdew, J. P., Ernzerhof, M. & Burke, K. Rationale for mixing exact exchange with density functional approximations. *J. Chem. Phys.* **105**, 9982–9985 (1996).

[43] Hybertsen, M. S. & Louie, S. G. Electron correlation in semiconductors and insulators: band gaps and quasiparticle energies. *Phys. Rev. B* **34**, 5390–5413 (1986). 10.1103/physrevb.34.5390 9940372

[44] Filip, M. R., Patrick, C. E. & Giustino, F. *GW* quasiparticle band structures of stibnite, antimonselite, bismuthinite, and guanajuatite. *Phys. Rev. B* **87**, 205125 (2013).

[45] Crothers, T. W. et al. Photon reabsorption masks intrinsic bimolecular charge-carrier recombination in CH_3_NH_3_PbI_3_ perovskite. *Nano Lett.* **17**, 5782–5789 (2017). 10.1021/acs.nanolett.7b02834 28792767

[46] Saba, M. et al. Correlated electron–hole plasma in organometal perovskites. *Nat. Commun.* **5**, 5049 (2014). 10.1038/ncomms6049 25266869

[47] ’t Hooft, G. W. & van Opdorp, C. Temperature dependence of interface recombination and radiative recombination in (Al, Ga)As heterostructures. *Appl. Phys. Lett.* **42**, 813–815 (1983).

[48] Schenk, A. A model for the field and temperature dependence of Shockley-Read-Hall lifetimes in silicon. *Solid State Electron.* **35**, 1585–1596 (1992).

[49] Würfel, P., Finkbeiner, S. & Daub, E. Generalized Planck’s radiation law for luminescence via indirect transitions. *Appl. Phys. A* **60**, 67–70 (1995).

[50] Hirst, L. C. & Ekins-Daukes, N. J. Fundamental losses in solar cells. *Prog. Photovolt. Res. Appl.* **19**, 286–293 (2011).

[51] Park, Y., Zhao, B. & Fan, S. Reaching the ultimate efficiency of solar energy harvesting with a nonreciprocal multijunction solar cell. *Nano Lett.* **22**, 448–452 (2022). 10.1021/acs.nanolett.1c04288 34939814

[52] Horton, M. K. et al. Accelerated data-driven materials science with the Materials Project. *Nat. Mater.* **24**, 1522–1532 (2025). 10.1038/s41563-025-02272-0 40610673

[53] Feng, J., Qian, X., Huang, C.-W. & Li, J. Strain-engineered artificial atom as a broad-spectrum solar energy funnel. *Nat. Photon.* **6**, 866–872 (2012).

[54] Sun, Y., Wang, D. & Shuai, Z. Indirect-to-direct band gap crossover in few-layer transition metal dichalcogenides: a theoretical prediction. *J. Phys. Chem. C* **120**, 21866–21870 (2016).

[55] Hamer, M. J. et al. Indirect to direct gap crossover in two-dimensional InSe revealed by angle-resolved photoemission spectroscopy. *ACS Nano* **13**, 2136–2142 (2019). 10.1021/acsnano.8b08726 30676744

[56] Mudd, G. W. et al. The direct-to-indirect band gap crossover in two-dimensional van der Waals Indium Selenide crystals. *Sci. Rep.* **6**, 39619 (2016). 10.1038/srep39619 PMC5180233 28008964

[57] Peng, X., Wei, Q. & Copple, A. Strain-engineered direct-indirect band gap transition and its mechanism in two-dimensional phosphorene. *Phys. Rev. B* **90**, 085402 (2014).

[58] Dhar, N. et al. Exfoliation behavior of van der Waals Strings: case study of Bi_2_S_3_. *ACS Appl. Mater. Interfaces* **10**, 42603–42611 (2018). 10.1021/acsami.8b14702 30426735

[59] Semenov, V. N., Ostapenko, O. V., Lukin, A. N., Zavalishin, E. I. & Zavrazhnov, A. Y. Solid-state reactions in CdS–Bi_2_S_3_ thin films. *Inorg. Mater.* **36**, 1197–1199 (2000).

[60] Gant, S. E. et al. Optimally tuned starting point for single-shot *GW* calculations of solids. *Phys. Rev. Mater.* **6**, 053802 (2022).

[61] Giannozzi, P. et al. Advanced capabilities for materials modelling with Quantum ESPRESSO. *J. Phys. Condens. Matter* **29**, 465901 (2017). 10.1088/1361-648X/aa8f79 29064822

[62] Giannozzi, P. et al. QUANTUM ESPRESSO: a modular and open-source software project for quantum simulations of materials. *J. Phys. Condens. Matter* **21**, 395502 (2009). 10.1088/0953-8984/21/39/395502 21832390

[63] Perdew, J. P., Burke, K. & Ernzerhof, M. Generalized gradient approximation made simple. *Phys. Rev. Lett.* **77**, 3865–3868 (1996). 10.1103/PhysRevLett.77.3865 10062328

[64] Hamann, D. R. Optimized norm-conserving Vanderbilt pseudopotentials. *Phys. Rev. B* **88**, 085117 (2013).

[65] van Setten, M. J. et al. The PseudoDojo: training and grading a 85 element optimized norm-conserving pseudopotential table. *Comput. Phys. Commun.* **226**, 39–54 (2018).

[66] Monkhorst, H. J. & Pack, J. D. Special points for Brillouin-zone integrations. *Phys. Rev. B* **13**, 5188–5192 (1976).

[67] Caracas, R. & Gonze, X. First-principles study of the electronic properties of A2B3 minerals, with A=Bi,Sb and B=S,Se. *Phys. Chem. Miner.* **32**, 295–300 (2005).

[68] Fonari, A. & Sutton, C. Effective mass calculator. *GitHub* https://github.com/afonari/emc (2025).

[69] Buizza, L. R. V. et al. Charge-carrier mobility and localization in semiconducting Cu_2_AgBiI_6_ for photovoltaic applications. *ACS Energy Lett.* **6**, 1729–1739 (2021). 10.1021/acsenergylett.1c00458 PMC8155390 34056108

[70] Seifert, T. et al. Efficient metallic spintronic emitters of ultrabroadband terahertz radiation. *Nat. Photon.* **10**, 483–488 (2016).

[71] Wagner, F. M. et al. Optimised spintronic emitters of terahertz radiation for time-domain spectroscopy. *J. Infrared Millim. Terahertz Waves* **44**, 52–65 (2023).

