# Supplementary material for: Delayed electron–hole recombination in a pseudo-direct semiconductor
Source: Nature. 2026 Oct 28;659(8139):76–82. doi: 10.1038/s41586-026-11223-x (PMC13736635; doi:10.1038/s41586-026-11223-x)
Supplement: Supplementary file 1 [file 41586_2026_11223_MOESM1_ESM.pdf]

nature portfolio

https://doi.org/10.1038/s41586-026-11223-x

Supplementary information

Delayed electron–hole recombination in a pseudo-direct semiconductor

In the format provided by the authors and unedited

Supplementary Information for

Delayed Electron-Hole Recombination in a Pseudo-Direct Semiconductor

*Marcello Righetto[1,2], Debalina Banerjee[1], Zhenglin Jia[3], Joshua R. S. Lilly[1], Vincent J.- Y. Lim[1], Snigdha Lal[1], Siyu Yan[1], Jae Eun Lee[1], Jay B. Patel[4], Michael B. Johnston[1], Qianqian Lin[3], Marina R. Filip[1], Laura M. Herz [1]**

[1.] Department of Physics, University of Oxford, Clarendon Laboratory, Parks Road, Oxford, OX1 3PU, United Kingdom

[2.] Department of Chemical Science, Università degli Studi di Padova, Via Marzolo 1, 35131, Padova, Italy

[3.] Key Lab of Artificial Micro- and Nano-Structures of Ministry of Education of China, School of Physics and Technology, Wuhan University, Wuhan 430072, Hubei, China

[4.] Department of Physics, King's College London, Strand, London, WC2R 2LS, United Kingdom

*Corresponding Author's Email: laura.herz@physics.ox.ac.uk

TABLE OF CONTENTS

Additional Details of Computational Methods 3
Table S1 – Convergence of the quasiparticle band gap at Γ with respect to k-point grid 3
Figure S1 – Convergence of the quasiparticle band gap at Γ with respect to polarizability cutoff and number of bands 4
Table S2 – Convergence of the first excited-state energy with respect to k-point grids and number of bands 5
Table S3 – Calculated lowest direct and indirect transition energies from PBE, PBE0, G_0W_0@PBE ... 6
Figure S2 – Experimental X-ray diffraction patterns of Bi_2S_3 thin films on different substrates 7
Supplementary Note 1 – Charge-carrier recombination dynamics 8
Figure S3 – Photoluminescence in Bi_2S_3 thin films 11
Supplementary Note 2 – Dispersive monomolecular recombination and implications for PL spectroscopy 12
Figure S4 – Temperature-dependent TRPL transients and radiative efficiency for Bi_2S_3 thin films ... 14
Figure S5 – Temperature-dependent OPTP transients for selected temperatures 15
Supplementary Note 3 – Radiative recombination rate simulations 16
Figure S6 – k_2 simulation inputs for direct and pseudo-direct semiconductors 22
Supplementary Note 4 – Derivation of charge-carrier mobility from OPTP and interpretation of temperature-dependent mobility trends 24
Figure S7 – Surface morphology of Bi_2S_3 thin films 26
Figure S8 – Photoconductivity spectrum for Bi_2S_3 thin films 27
Table S4 – Effective masses of Bi_2S_3 near the band edges 29
Supplementary Note 5 – Impact of pseudo-direct bandgap character on solar energy conversion devices 30
Figure S9 – Diffusion length calculations for Bi_2S_3 thin films 31
Supplementary References 32

Additional Details of Computational Methods

GW/BSE calculations

We calculate the quasiparticle band structure of Bi_2S_3 within the single-shot G_0W_0 approximation (*1*), as implemented in the BerkeleyGW code (*2, 3*), with a DFT/PBE starting point, including spin-orbit coupling. We approximate the frequency-dependent dielectric function using the Godby-Needs plasmon pole model(*4*). We use a dielectric function cutoff of 6 Ry, and sum over 600 bands (408 unoccupied) with a Γ-centered coarse grid of 4x8x4 to calculate the quasiparticle self-energy corrections. These parameters are consistent with prior work(*5*).

We have checked numerical convergence of the band gap with respect to the number of bands and dielectric function cutoff by testing parameters between 300 and 800 bands, and sweeping the cutoff between 6 Ry and 16 Ry, as shown in Figure S1. We found that within this range, the quasiparticle band gap at Γ converges slowly, and changes by less than 50 meV, as shown in Figure S1. In order to contain the computational effort for subsequent BSE calculations, and given that band gaps appear to vary slowly with the increase of these two parameters, we have chosen to restrict the number of bands to 600 and the polarizability cutoff to 6 Ry. Furthermore, we have also checked convergence with respect to the density of the k-point grid, reported in Table S1.

K-point grid	Number of bands	Polarizability cutoff (Ry)	Band gap at Γ (eV)
2x2x4	800	12	1.24
2x4x2	800	12	1.27
2x4x2	600	6	1.24
**4x8x4**	**600**	**6**	**1.24**

Table S1 | Convergence of the quasiparticle band gap at Γ with respect to k-point grid. All grids tested are centered at Γ. Values highlighted in bold are the parameters used in the final calculation.

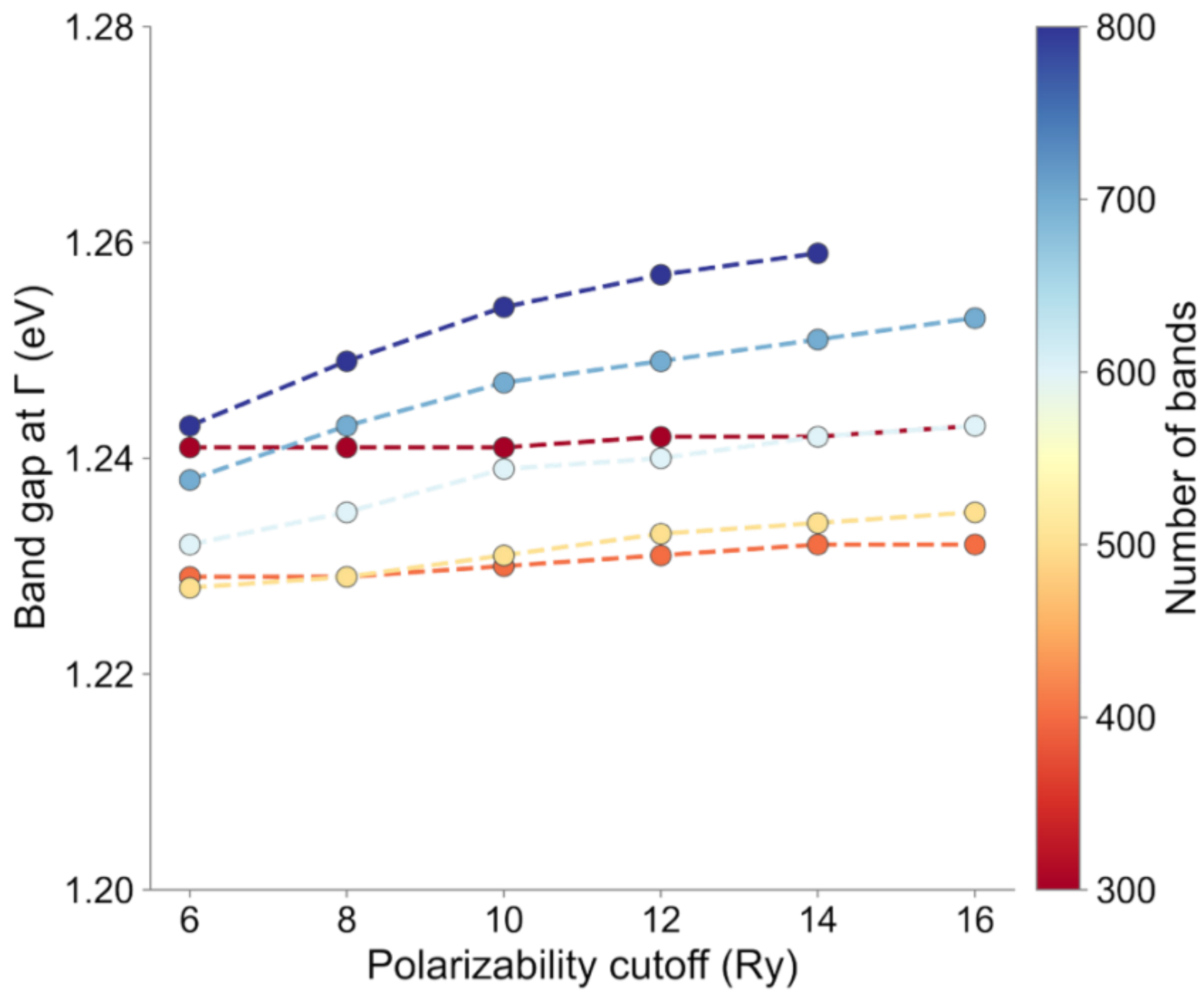

Figure S1 | Convergence of the quasiparticle band gap at Γ with respect to the polarizability cutoff and the number of bands. Calculations are performed using a Γ-centered kpoint grid of 2x2x4.

We calculate the linear optical absorption spectrum of Bi_2S_3 by solving the Bethe-Salpeter Equation (BSE)(*6*), as implemented in the BerkeleyGW code. Calculation of the optical absorption spectrum includes only direct transitions, and does not account for phonon-assisted processes. We calculate the BSE kernel on a Γ-centered grid of 4x8x4, including 12 valence and 16 conduction bands. We interpolate this kernel on a fine, Γ-centered grid of 8x16x8, before we diagonalize the BSE hamiltonian, and compute the real and imaginary part of the dielectric function, including 8 valence and 12 conduction bands. This computational setup yields energies of the first excited state which are converged within less than 50 meV, as shown in Table S2.

Coarse k-point grid	Valence bands (coarse grid)	Conduction bands (coarse grid)	Fine k-point grid	Valence bands (fine grid)	Conductio n bands (fine grid)	1st excited state energy (eV)
2x4x2	12	16	4x8x4	8	12	1.131
2x4x2	12	16	4x8x4	8	16	1.130
2x4x2	12	16	8x16x8	8	12	1.133
2x4x2	16	16	4x8x4	12	12	1.133
2x4x2	28	20	4x8x4	24	16	1.138
**4x8x4**	**12**	**16**	**8x16x8**	**8**	**12**	**1.124**

Table S2 | Convergence of the energy of the first excited state with respect to coarse and fine k-point grids, and the number of valence and conduction bands included in each calculation. All grids tested are centered at Γ. Values highlighted in bold are the parameters used in the final calculation.

We calculate the optical absorption spectrum using the expression (7) $\alpha(\omega) = \frac{\omega}{c}\sqrt{2\left(\sqrt{\varepsilon_1(\omega)^2 + \varepsilon_2(\omega)^2} - \varepsilon_1(\omega)\right)}$, where ω is the photon frequency, ε_1 and ε_2 are the real and imaginary parts of the dielectric function, respectively, calculated as described above, and averaged over all polarization directions. The optical absorption spectrum reported in Figure 1, is calculated using an arbitrary broadening of 50 meV.

Functional	Direct E_g [eV]	Fundamental Indirect E_g [eV]	E_{diff} [meV]
**PBE (dense grid)**	1.004<br>(0, 0, 1/4)	0.969<br>VBM: (1/3, 0, 1/12) and (1/3, 0, 0)<br>CBM: (0, 0, 1/6)	35
**PBE (sparse grid)**	1.018<br>(0,0,1/4)	1.002<br>VBM: (1/4, 0, 0)<br>CBM: (0, 0, 1/4)	16
**PBE0 (sparse grid)**	2.038<br>(0, 0, 1/4)	1.999<br>VBM: (1/4, 0, 0)<br>CBM: (0, 0, 0)	39
G_0W_0@PBE (sparse grid)	1.160<br>(0, 0, 1/4)	1.130<br>VBM: (1/4, 0, 0)<br>CBM: (0, 0, 0)	30

Table S3 | Calculated lowest direct and indirect transition energies from PBE, PBE0 and G0W0@PBE Lowest direct and indirect transition energies calculated using the standard semi-local PBE functional and the global hybrid functional PBE0 including spin-orbit coupling. For the dense grid calculation, we used a Γ centered 36x1x36 k-point grid to locate the band edges (informed by the band structure in Figure 1). We found two nearly degenerate indirect bandgaps, corresponding to two valence band maxima, and one conduction band minimum. For the sparse grid calculations, we used Γ-centered k-point-grid of 4×6×4, commensurate with the hybrid PBE0 calculations performed with the same k- and q-point grids. GW band gaps are calculated on a 4x8x4 grid centered at Γ. E_{diff} in the table corresponds to the difference between the direct band gap and the fundamental indirect band gap (where available), as well as the direct band gap and the lowest indirect band gap between respective high symmetry points. For each band gap we also report the corresponding reciprocal space location in units of the reciprocal lattice.

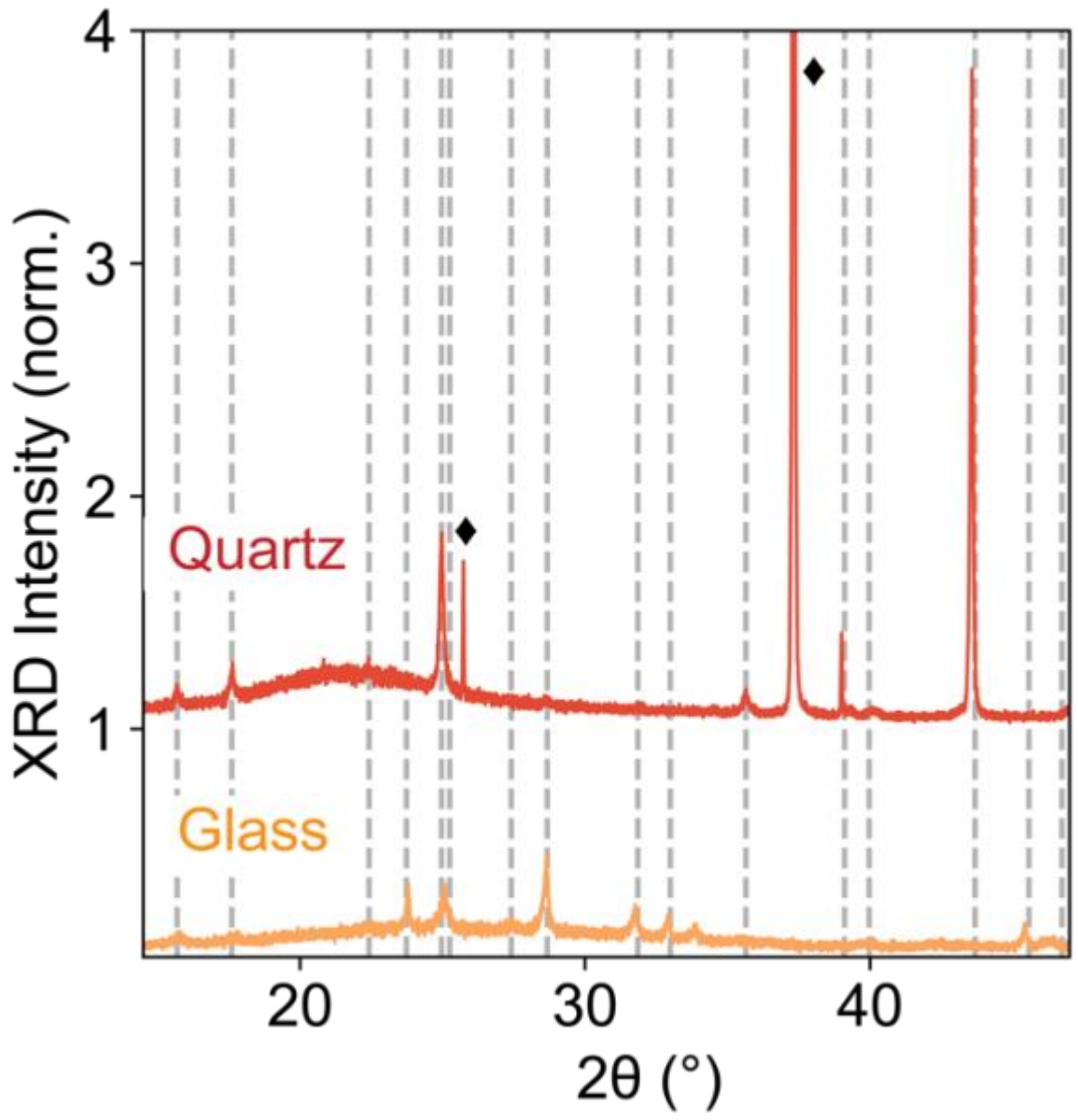

Figure S2 | Experimental X-ray diffraction patterns of Bi_2S_3 thin films on different substrates. Experimental X-ray diffraction pattern of evaporated Bi_2S_3 thin films on quartz (red line) and glass (yellow line) substrates, measured using Cu K-α_1 radiation. Vertical dashed lines indicate the reference peak positions for Bi_2S_3 (ICDD Coll. Code 89324). Diamond symbols indicate substrate peaks.

Supplementary Note 1: Charge-carrier recombination dynamics

Determining the charge-carrier recombination rate constants is paramount to disentangling extrinsic and intrinsic charge-carrier recombination processes. The well-established k_1, k_2, k_3 (also known as ABC) model describes the total charge-carrier recombination rate in a semiconductor as the sum of first- (monomolecular), second- (bimolecular), and third-order (Auger) recombination contributions (*8, 9*). Such a model has proven effective in distinguishing different charge-carrier recombination processes in several semiconductors (*9-11*) through injection-dependent (or fluence-dependent) measurements of charge-carrier dynamics. In fluence-dependent measurements, different charge-carrier recombination mechanisms contribute to a different extent depending on the excess charge-carrier density (vide infra).

Given the fluence range (up to tens of μJ cm^{-2}) of our optical-pump THz-probe (OPTP) measurements, we limited the model to monomolecular and bimolecular recombination processes and assumed negligible Auger contributions ($k_3n^3 \sim 0$) to the observed recombination (*8*). Therefore, the resulting charge-carrier dynamics can be described by the following rate equation:

$$\frac{dn}{dt} = -k_1 n - k_2 n^2, \quad \text{(S1)}$$

where n is the charge-carrier density, k_1 is the monomolecular recombination constant, which we ascribe to trap-mediated recombination processes, and k_2 is the bimolecular recombination constant, ascribed to the band-to-band radiative electron-hole recombination process.

Several theoretical and experimental studies on charge-carrier trapping in disordered semiconductors have found that the trap-mediated monomolecular recombination rate exhibits a dispersive (i.e., with a time constant that varies with time) character and can be satisfactorily

described by a stretched (or Kohlrausch) exponential function (*12-14*). Hence, to describe the monomolecular recombination in Bi_2S_3 thin films, we adopt the Kohlrausch-Williams-Watts (KWW) decay rate $k_1(t) = \beta\tau_0^{-\beta}t^{\beta-1}$,(*15*) which, in the absence of any other deexcitation processes, yields stretched-exponential transients $I(t) = I_0 \exp\left[-(t/\tau_0)^{\beta}\right]$. Here, the stretching parameter β gauges deviations from the canonical single exponential decay, and can vary between 0 and 1, with $\beta \to 1$ being the single exponential case. As shown in Figure 2A of the Main Text, we find that such decay well describes the charge-carrier dynamics over a long time window with a $\beta{\sim}0.5$.

We numerically solved Equation S1 in terms of the experimentally observed quantity $x(t)$ = $\Delta T/T$, which is the photoinduced change in THz transmission (*16*). Here, the charge-carrier density $n(t)$ is proportional to $x(t)$ with the following relationship:

$$n(t) = \varphi C x(t) \tag{S2}$$

Here φ is the photon-to-charge branching ratio, and $C = N_0/x(0)$ is the ratio between the instantaneous OPTP response $x(0)$ and the absorbed photon density for thin films thicker than $1/\alpha(\lambda)$ (~130 nm for Bi_2S_3 at 800nm-excitation) where N_0 can be expressed as:

$$N_0 = \frac{E\lambda}{hcA_{\text{eff}}}\alpha(\lambda)\left(1 - R_{\text{pump}}\right), \tag{S3}$$

where A_{eff} is the effective area of overlap between the THz and the pump beams. As reported by Xia et al.(*17*), this is defined as $A_{eff} = 2\pi\left(\sigma_{pump}^2 + \sigma_{THz}^2\right)$, where σ_{pump} and σ_{THz} are the pump and THz beam waists, respectively. In our model, we note that the spatially varying charge-carrier density profile generated by the visible pump photoexcitation is described according to the Lambert-Beer law. Therefore, in our fitting algorithm, the resulting exponentially decaying charge-carrier density is taken into account by dividing the thin films

into 10 equally thick (i.e., 15 nm) slices. Equations S1, S2 and S3 are combined and solved numerically for each slice. Using this method, the differential equation is globally fitted to the fluence-dependent OPTP datasets (see Extended Data Fig. 4, 5).

As a result, our fluence-dependent OPTP measurements enable effective decoupling of monomolecular and bimolecular recombination processes in Bi_2S_3. Such decoupling arises from the distinct dependencies on the charge-carrier density n (see Equation S1), which is varied via the excitation fluence parameter (see Equation S3). Here, owing to the different n-scaling of the mono- and bimolecular terms, increasing excitation fluence enhances recombination through the bimolecular channel relative to the monomolecular channel. Such behaviour is observed as an accelerating photoconductivity decay at early times, which leaves a photoconductivity tail dominated by monomolecular processes. Such a process underlies the characteristic "fanning-out" of normalised transients with increasing fluence that is clearly observed in our Bi_2S_3 datasets (Figure 2D) and represents a hallmark of bimolecular electron–hole recombination.

Crucially, such fluence-dependent behaviour is incompatible with the two main mechanisms often invoked to explain charge-carrier-density-dependent dynamics related to defect-mediated recombination, namely disorder-broadened monomolecular recombination and trap saturation. On the one hand, the effect of disorder is fully captured in our model by the KWW formulation of the k_1 parameter, as disorder mainly broadens the distribution of monomolecular rates but does not change the order of the rate law. On the other hand, in the presence of finite trap densities, photogenerated charge carriers can fill the available trap states in a process known as trap saturation (*18-20*). The result of this process is an effectively suppressed monomolecular recombination rate. It is worth noting that trap-saturation effects yield slower decays at higher fluence, which is the opposite behaviour to what is observed in our Bi_2S_3 OPTP transients. Therefore, the observed acceleration of photoconductivity decay at increasing

excitation fluences excludes trap saturation as the origin of the fluence dependence and requires a second-order term in the rate law.

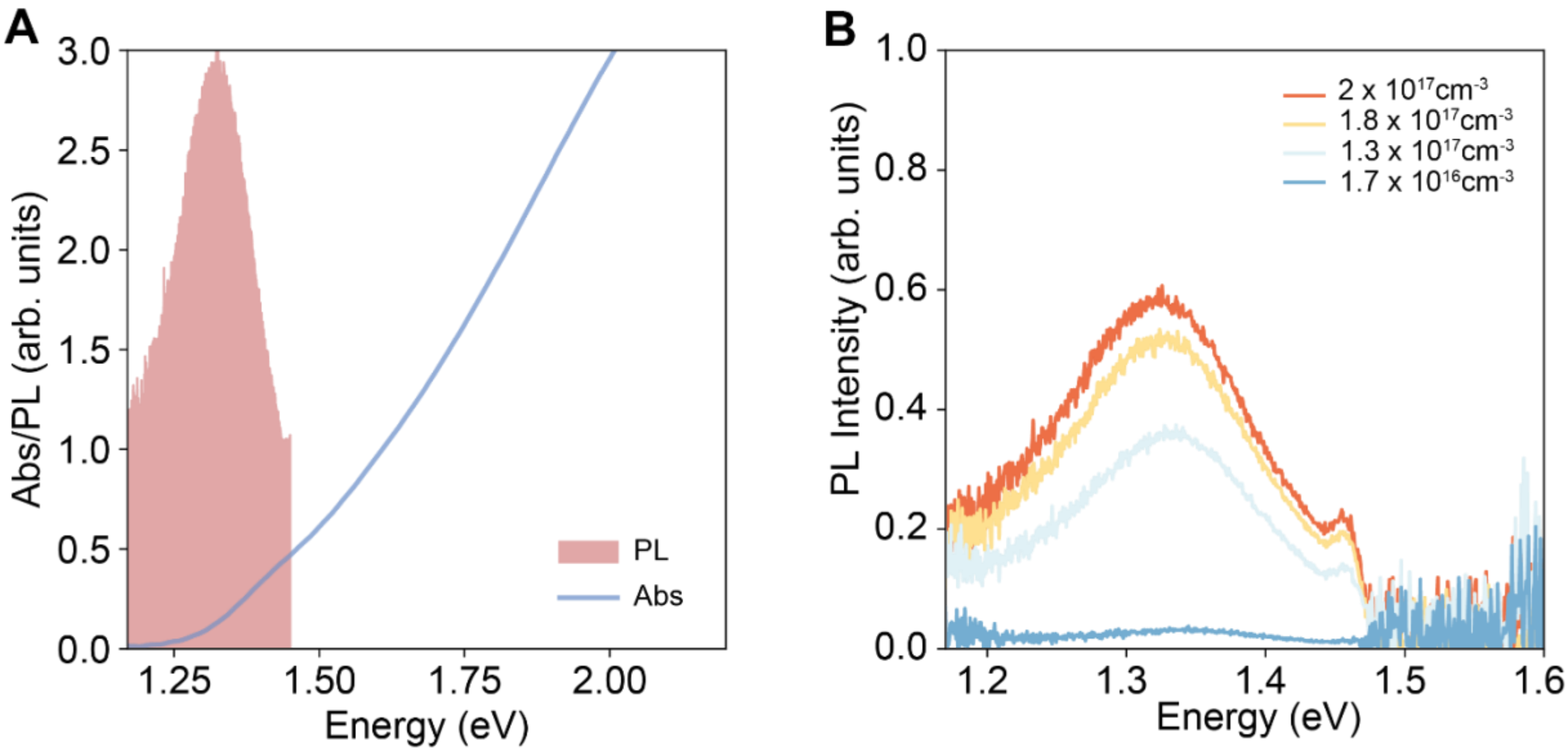

Figure S3 | Photoluminescence in Bi_2S_3 thin films. (**A**) Photoluminescence spectrum (red shaded area) measured for Bi_2S_3 thin films on quartz following 1.67-eV, 35-fs pulsed excitation. For comparison purposes, the absorption spectrum of Bi_2S_3 thin films is plotted as a blue solid line. Measurements were performed using a Si-CCD detector coupled with a spectrometer, and a 1.5 eV cutoff filter was used to reduce the excitation laser scattering. (**B**) Comparison between photoluminescence spectra measured for Bi_2S_3 thin films on quartz following 1.67-eV, 35-fs pulsed excitation, exciting a different initial charge-carrier population in the range of 10^{16}-10^{17} cm^{-3}, indicated in the legend. A 1.5eV cutoff filter was used to reduce the excitation laser scattering.

Supplementary Note 2: Dispersive monomolecular recombination and implications for PL spectroscopy

As discussed in Supplementary Note 1 and in the Main Text, the dispersive nature of trap-mediated recombinations in Bi_2S_3 thin films causes initially fast (on the picosecond timescale) monomolecular recombination processes. The presence of these fast recombination processes carries significant implications for the extraction of bimolecular recombination rates. As demonstrated by Sarritzu et al. and others (*21-23*), the prompt PL intensity (per absorbed photon) emitted immediately following photoexcitation – $PL(t = 0)$ – can in principle be used as a direct proxy of the radiative bimolecular recombination rate $k_{2,rad}$. Such a method has been successfully used to decouple the temperature dependence of k_1 and k_2 in lead halide perovskites.(*21*) However, we note that in the presence of these fast monomolecular recombination, the finite time resolution of conventional time-resolved photoluminescence detection schemes – usually around a few nanoseconds for time-correlated single photon counting and electronic gating – can limit the applicability of this method. Here, limitations arise from the considerable monomolecular contribution to the decay within the instrument response function (IRF), which causes the $PL(t = IRF)$ value to be contributed by both k_1 and k_2. Such overlapping contributions can therefore conceal the real k_2 temperature dependence. As shown in Figure S4A, the TRPL decay that we observed is likely dominated by the instrument response function of our systems (~ 3ns, see Reference (*22*) for further description). Interestingly, we find that the TRPL peak decreases at lower temperatures. However, when the temperature-dependent change in the absorption coefficient is included to estimate prompt PL (Figure S4B), the trend becomes less clear, with an initial increase from 295 to 150K, followed by a decrease at lower temperatures. We posit that this trend originates from the complicated interplay of the KWW k_1 and k_2 rates. To further support this, we estimated the radiative efficiency using the recombination rates obtained from our k_1k_2 model. When using pulsed

excitation, the radiative efficiency or photoluminescence quantum efficiency can be defined as(*24*):

$$\eta_{em} = \frac{\int_0^\infty k_2 n(t)^2 dt}{n_0}, \qquad \text{(S4)}$$

Where n_0 is defined as $n_0 = \int_0^\infty [k_1(t)n(t) + k_2 n(t)^2 + k_3 n(t)^3] dt$. As shown in Figure S4C, the resulting η_{em} increases with increasing charge-carrier density, owing to the k_2 term and finally peaks and decreases. While nonradiative recombination is driven by the k_1(t) term at low charge-carrier density, at higher charge-carrier density the k_3 term (assumed here to be $k_3 \sim 10^{-31}\ cm^6 s^{-1}$) causes the reduction. The estimated radiative efficiency for Bi_2S_3 thin films, Figure S4D, based on the k_1(t) and k_2 parameters obtained by the temperature-dependent OPTP fits (Figure 3, Extended Data Figures 4, 5) and obtained by solving Equation S4 for a given charge-carrier density ($n_0 = 10^{15}\ cm^{-3}$) reveals a non-monotonous dependence. Interestingly, as with the prompt PL, the radiative efficiency increases as the temperature decreases from 295 to 150 K and decreases at lower temperatures. We note that this finding supports the hypothesis that, owing to the finite time resolution of the TRPL setup and the fast, dispersive trapping processes, the observed prompt PL values integrate both k_1 and k_2 contributions.

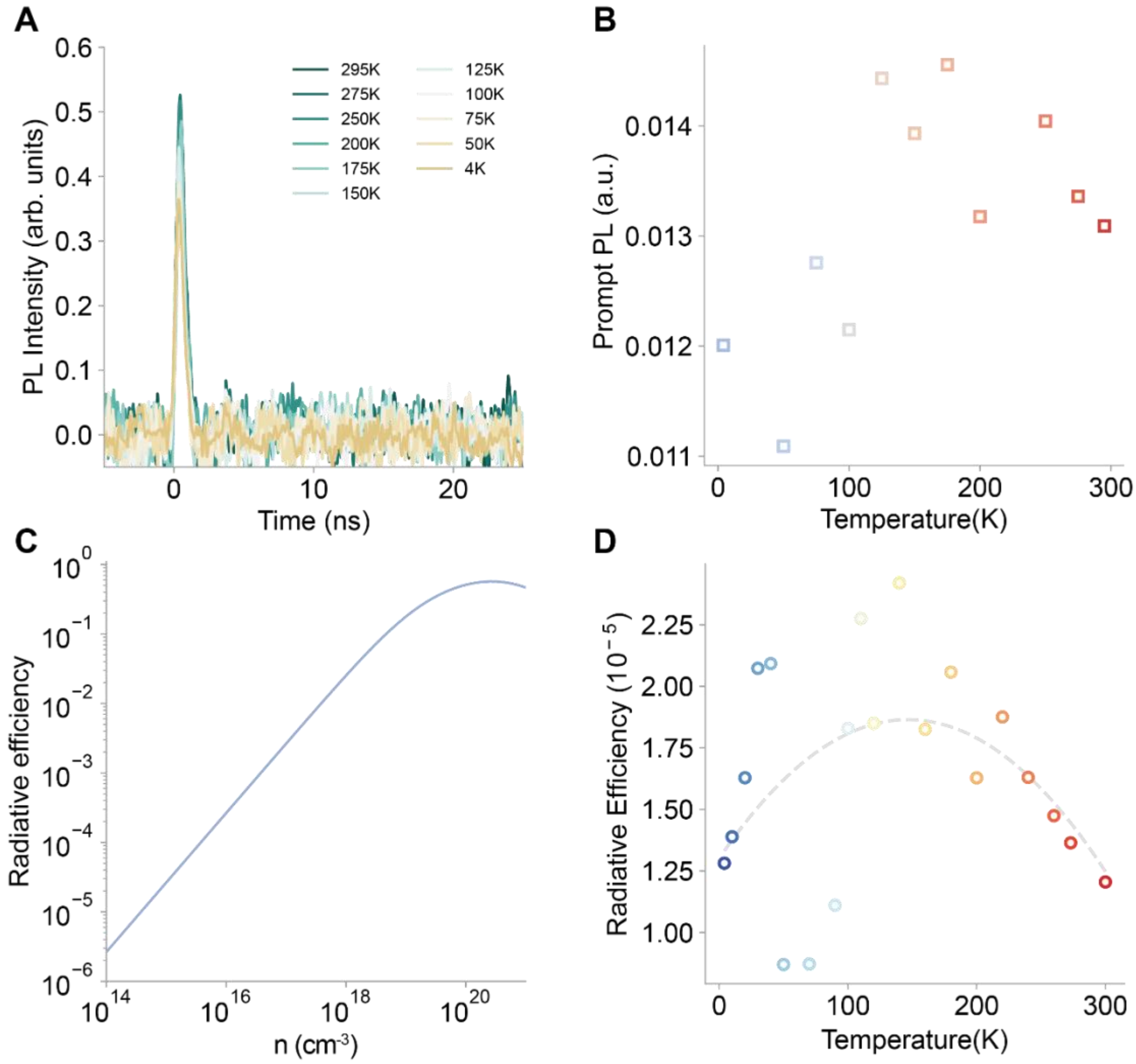

Figure S4 | Temperature-dependent TRPL transients and radiative efficiency for Bi_2S_3 thin films. (**A**) Comparison between early-time TRPL transients measured for Bi_2S_3 thin films on quartz different at temperatures following 1.67-eV, 35-fs pulsed excitation with a fluence of 60 µJ cm^{-2}, and detected integrated across the whole spectrum after suppression of the excitation with a 1.5 eV bandedge filter. (**B**) Temperature evolution of the prompt PL intensity – i.e., the initial PL intensity, PL (t=0), recorded immediately following excitation, per absorbed photon – extracted from TRPL transients. (**C**) Calculated radiative efficiency based on rate constants from OPTP fitting parameters (Figure 2B, Main text) as a function of initial charge-carrier density. The radiative efficiency value was computed by solving Equation S4. (**D**) Calculated radiative efficiency based on rate constants from temperature-dependent OPTP fitting parameters (Figure 3, Extended Data Fig. 4, 5) for an initial charge carrier density of 10^{15} cm^{-3}. Dashed line is a guide to the eye.

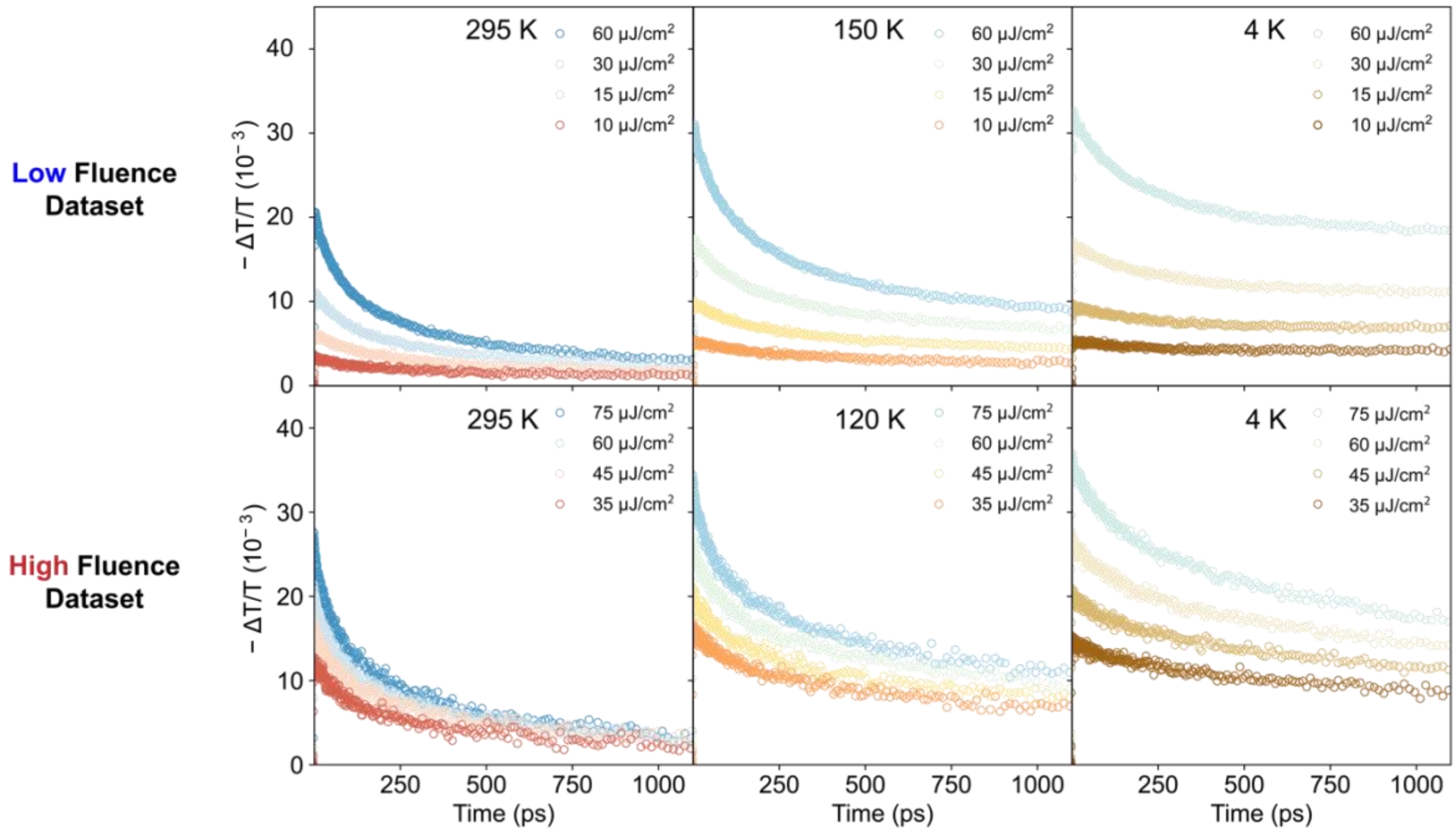

Figure S5 | Temperature-dependent optical-pump-THz-probe (OPTP) transients for selected temperatures. Qualitative comparison between fluence-dependent OPTP transients measured for Bi_2S_3 thin films following 1.55-eV pulsed photoexcitation at selected temperatures. The upper and lower panels represent two datasets recorded using different fluence ranges.

Supplementary Note 3: Radiative recombination rate simulations

The van Roosbroeck and Shockley (vRS) theory builds on the detailed balance principle to define the radiative recombination rate (R_{rad}) in a semiconductor. Following the vRS theory, R_{rad} within the energy interval dE centred at energy E is found to be equal to the electron-hole pair generation rate at that energy under thermal equilibrium conditions. The vRS theory was developed to describe thermal generation processes and has been extended to non-thermal (i.e., light-induced) generation processes by Würfel and coworkers (*25, 26*). The generation rate can be defined as the product between the density of photons in the material in the energy interval dE, $\rho(E,T)d(E)$, and the probability per unit time that a photon of energy E is absorbed $P(E,T)$. Therefore, following the vRS theory, the total radiative recombination rate per unit of volume can be obtained by integrating over E the generation rate:

$$R_{\mathrm{rad}}(T) = \int_{0}^{\infty} \rho(E,T)P(E,T)dE \tag{S5}$$

By using Planck's law (*25, 27*), the generation rate per unit of energy (*28*) may be defined as:

$$\rho(E,T)P(E,T)dE = \frac{\alpha(E,T)}{\hbar^3}\left(\frac{n_{\mathrm{r}}E}{\pi c}\right)^2 \frac{1}{e^{E/k_BT}-1}dE \tag{S6}$$

where n_{r} is the average refractive index, $\alpha(E,T)$ is the absorption coefficient. For energies around the bandgap – since $e^{E/k_{\mathrm{B}}T} \gg 1$ – we can approximate the Bose-Einstein term to a Boltzmann $e^{-E/k_{\mathrm{B}}T}$ term. Thus, the total radiative recombination rate is:

$$R_{\mathrm{rad}}(T) = \int_{0}^{\infty} \frac{\alpha(E,T)}{\hbar^3}\left(\frac{n_rE}{\pi c}\right)^2 e^{-E/k_{\mathrm{B}}T}\,dE \tag{S7}$$

Following Davies et al. (*28*), the bimolecular recombination rate can be obtained from the total recombination rate by dividing by the intrinsic charge-carrier density:

$$k_2(T) = \frac{R_{\mathrm{rad}}(T)}{n_{\mathrm{i}}^2} \tag{S8}$$

where n_i is the intrinsic charge-carrier density. By assuming an intrinsic semiconductor, Davies et al. (*28*) calculates n_{i} as the product of intrinsic electron and hole concentration, n_0 and p_0, in terms of the electron and hole effective masses, m_{e}^* and m_{h}^* respectively, and the energy gap E_{g}

$$n_i = \sqrt{n_0 p_0} = 2\left(\frac{k_B T}{2\pi\hbar^2}\right)^{3/2} (m_{\mathrm{e}}^* m_{\mathrm{h}}^*)^{3/4} e^{-E_{\mathrm{g}}/2k_{\mathrm{B}}T} \tag{S9}$$

The derivation provided by Davies et al. (*28*), builds on the well-established vRS theory (*27*) and applies to the case of thermally excited semiconductors. An analogous derivation applied to the non-thermal case has been provided by Würfel (*25*). In this theory, the chemical potential of photons μ is defined as the quasi-Fermi level splitting. We note that the additional Boltzmann weighing term from the chemical potential appears equally in the numerator and denominator, thus cancelling out and making resulting k_2 values independent on the source of excitation (i.e., thermal or non-thermal).

To demonstrate the role of multiple, closely-spaced band edges on the charge-carrier dynamics, we evaluated Equation S8 in the case of direct and pseudo-direct semiconductors. As illustrated in Figure S6, our simulation assumes parabolic bands, giving rise to a square-root energy dependence of both the absorption coefficient and the density-of-states. Conversely, in the case of the pseudo-direct semiconductor, we assumed an indirect polynomial absorption tail with an

amplitude $\alpha_{\text{ind}} \sim \alpha_{\text{dir}}/r$, where r is a constant, to account for the phonon-assisted character of indirect absorption processes. Our simulation solved Equation S8 as a function of temperature *T*. Furthermore, we performed different simulations for different direct-to-indirect energy splittings ΔE in the 30-200 meV range and an arbitrarily chosen, fixed $r = 300$. Here, we have deliberately explored an extended ΔE range, which is well beyond the splitting relevant to Bi_2S_3, with the aim of mapping full crossover from direct to pseudo-direct, and finally to conventional indirect behaviour.

Crucially, as clearly shown in Figure S6C, intrinsic charge carriers are spread across the band-edge energies at room temperature in a purely direct-gap system. At lower temperatures, the narrowing of the Fermi-Dirac distribution function causes a reduction in the intrinsic charge-carrier density, which yields increasing k_2 values at lower temperatures. This result reproduces those reported by Davies et al. (*28*) for direct band-gap lead halide perovskites and by Nguyen et al. (*29*) for indirect band-gap silicon. On the other hand, as shown in Figure S6D for pseudo-direct semiconductors, intrinsic charge carriers at room temperature populate both the bands contributing to direct and indirect absorption processes. When the temperature is reduced, the narrowing of the Fermi-Dirac distribution function causes a depopulation of the band contributing to the direct recombination pathway, thus yielding decreasing k_2 values at lower temperatures. As shown in Figure 3C of the main text, when such a higher-lying band is entirely depopulated, the increasing k_2 temperature trend is retrieved with reducing temperature as it will then be dominated by narrowing of the Fermi-Dirac distribution function near the points associated with solely the indirect transition.

To quantify the effect of retarded electron-hole recombination arising from the pseudo-direct bandgap character, we calculated a k_2 suppression ratio, defined as $1 - (\frac{k_{2,\text{pseudo}}}{k_{2,\text{dir}}})$, as a function of the direct-indirect energy splittings ΔE. Here, $k_{2,\text{dir}}$ is calculated by applying Equation S8

to the case of a conventional direct semiconductor (see yellow curves in Figure S6 A,B), while $k_{2,pseudo}$ is calculated by applying Equation S8 to the case of a pseudo-direct semiconductor (see pink curves in Figure S6 A,B) for a range of different ΔE values. As shown in Extended Data Fig. 7, within the assumptions of our model, $\Delta E \sim 65$ meV can potentially retard the electron-hole recombination by one order of magnitude. Importantly, we observe a saturation effect at high ΔE values ($\Delta E > 100$ meV) as a result of the direct recombination channel becoming thermally inaccessible, and consequently k_2 approaching the values set by the indirect channel. Therefore, further increases in ΔE yield only marginal suppression, while increasing the range of the absorption dominated by indirect transitions (see Figure S6A) and, with it, the associated photovoltage penalty. Therefore, we note that the benefits of the pseudodirect character are mostly achieved within splittings of a few k_BT.

Lastly, we stress that the suppression of k_2 in pseudo-direct semiconductors arises from charge carriers populating bands, giving rise to both direct and indirect transitions. Given that bimolecular recombination is significantly impaired in the case of indirect transitions, the respective bands can effectively act as a transient reservoir of charge carriers if the thermal energy is sufficient ($\Delta E \sim k_B T$) for them to establish an equilibrium with a band participating in a direct transition (*30*). Therefore, we propose such a condition ($\Delta E \sim k_B T$) to be defining of a different type of semiconductor, referred to as a pseudo-direct semiconductor, whose identifying features are strong direct absorption coefficients coupled with retarded electron-hole recombination. We stress that the pseudo-direct character definition here is probabilistic rather than a sharp cut-off: ΔE must be large enough for reducing the thermal population of the band-edges associated with the direct recombination, yet ΔE must also be kept as small as possible to ensure efficient absorption via the intense direct transition. As shown in Figure 3D, our fitting further supports the hypothesis that Bi_2S_3 is an exponent of this type of semiconductors. The observed temperature dependent k_2 trend is compatible with the presence

of an indirect transition with a two-orders-of magnitude lower absorption coefficient $r{\sim}250$ that lies $\sim 38\ meV$ below the direct one.

Relation to photovoltaic performance. The generalised Planck framework presented above also, in principle, permits estimates of the radiative-limit open-circuit voltage. Overall, in the presence of a direct-indirect bandgap combination, a boost in charge-carrier extraction and fill factor properties, close to the radiative limit, can be expected. Such gains may be particularly substantial for materials exhibiting lower long-range charge-carrier mobilities (*30*) that are often common for inorganic semiconductors with layered structures.

Role of the momentum offset (Δ*k*) between the band edges. In principle, the pseudo-direct character of Bi_2S_3 is not only defined by the ΔE parameter, but also by a momentum offset Δk parameter, which accounts for the different position of the involved band-edges in the first Brillouin zone. Crucially, radiative band-to-band recombination is bound to conserve crystal quasi-momentum *k*. Owing to the negligible momentum carried by a band-edge photon ($\sim 10^4 - 10^5\ cm^{-1}$ for band edge transitions in Bi_2S_3), with respect to the dimensions of the Brillouin zone ($\sim 10^7 - 10^8\ cm^{-1}$), radiative transitions are effectively vertical in *k*-space (Δk = 0). Consequently, the recombination channel associated with the indirect transition requires the assistance of a momentum-conserving phonon and is therefore orders of magnitude slower than the direct one.

Crucially, while the Δk value thus determines the direct or indirect character of the recombination, we posit that momentum conservation is not the rate-limiting factor for the temperature dependence of k_2. Owing to the large discrepancy between the direct and indirect recombination rates, the direct channel dominates recombination whenever it is thermally accessible, and therefore the temperature dependence of k_2 is instead governed by the energy offset ΔE, which controls the Boltzmann population of the higher-lying direct states. We note that our hypothesis builds on the assumption that charge carriers maintain a quasi-thermal-

equilibrium population across band edges that are closely spaced in energy. The underlying requirement is that intervalley scattering must be fast compared with the radiative lifetime. Importantly, this condition is generally satisfied, as intervalley scattering in semiconductors occurs on the tens of femtosecond timescale(*31*), which is significantly faster than bimolecular radiative recombination.

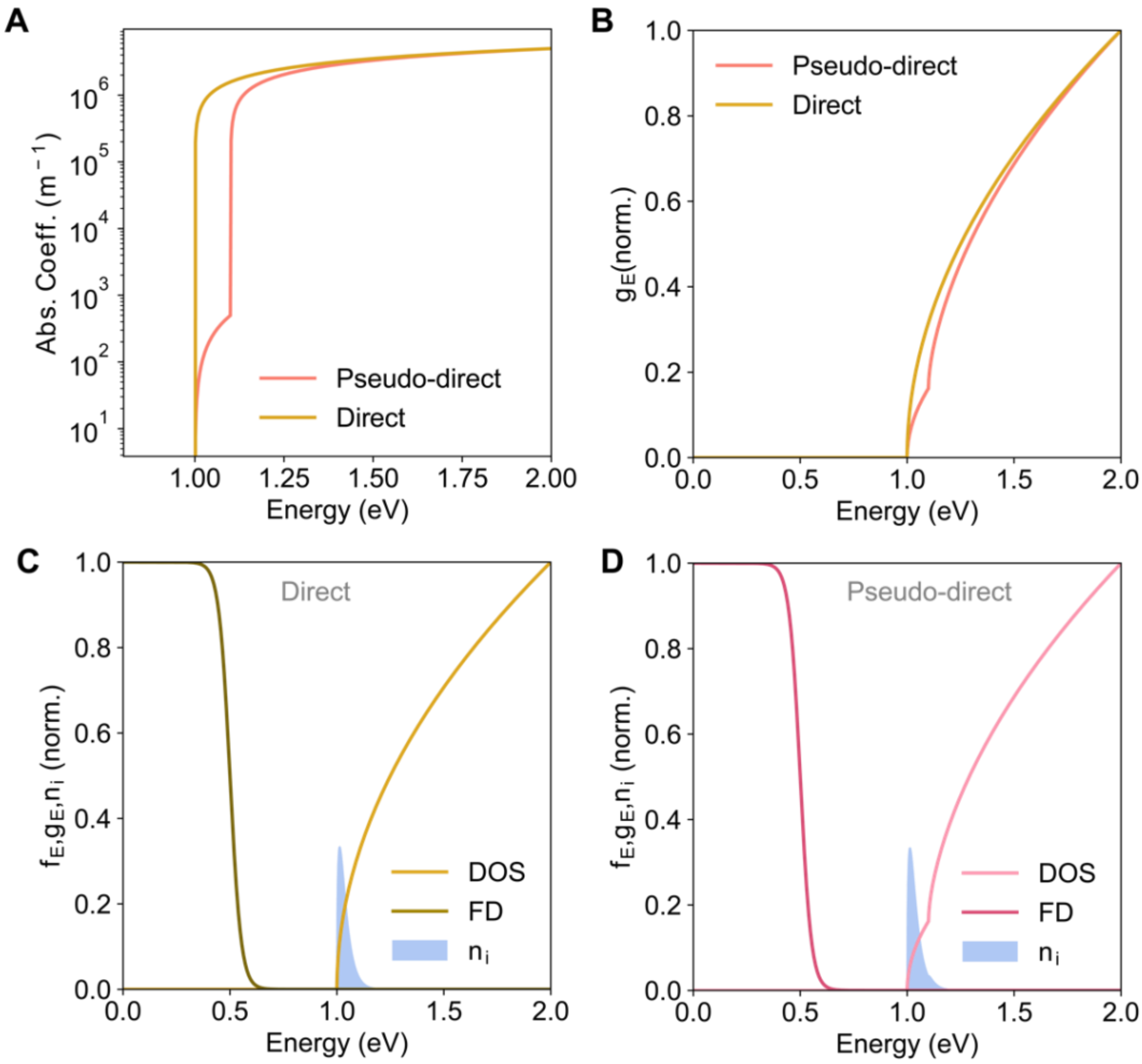

Figure S6 | k_2 simulation inputs for direct and pseudo-direct semiconductors. (**A**) k_2 simulation input absorption coefficient for a direct (yellow line) and a pseudo-direct (pink line) semiconductor. For the direct semiconductor, a conventional square-root dependence of the absorption coefficient is assumed. For the pseudo-direct semiconductor, a weak indirect absorption tail (plotted here as three orders of magnitude – r = 1000 – weaker than the direct term) is set at $\Delta E = 100$meV below the direct absorption onset. (**B**) k_2 simulation input density of states for a direct (yellow line) and pseudo-direct (pink line) semiconductor. For the direct semiconductor, a conventional square-root dependence of the density of states is assumed. For the pseudo-direct semiconductor, the density of states is the sum of two closely spaced bands $\Delta E = 100$meV, each carrying a square-root dependent contribution. In the simulations (Figure 3C of the main text), the ΔE parameter is varied. (**C**) Input intrinsic charge-carrier density in the case of a direct semiconductor calculated by solving Equation S9 at T=300 K. (**D**) Input

intrinsic charge-carrier density in the case of pseudo-direct semiconductors calculated by solving Equation S9 at T=300 K. We note that at room temperature, both direct and indirect bands are populated at the band-edge, i.e., a non-negligible fraction of intrinsic charge carriers can be found with energy ΔE spacing above the energy gap.

Supplementary Note 4: Derivation of charge-carrier mobility from OPTP and interpretation of temperature-dependent mobility trends

Temperature-dependent charge-carrier mobility values for Bi_2S_3 thin films shown in Figure 3B of the Main Text were determined from OPTP measurements by using the approach developed by Wehrenfennig et al. (*32*) and explained in detail by Xia et al. (*17*). Here, for thin film semiconductor materials with thicknesses smaller than the wavelength of the incident THz radiation, the sheet photoconductivity can be derived from the fractional change in the transmitted THz electric field $\Delta T/T$, and can be expressed as

$$\Delta S = -\epsilon_0 c(n_1 + n_3)\left(\frac{\Delta T}{T}\right) \tag{S10}$$

where $n_3 = 2.13$ and $n_1 = 1$ are the refractive indexes of quartz and vacuum, respectively, at THz frequencies (*33*). For temperature-dependent OPTP measurements, n_3 values have been assumed constant, given the weak temperature dependence measured on analogous quartz substrates (*34*).

The effective charge-carrier mobility can be derived from the sheet photoconductivity by calculating the number of initially photogenerated carriers, defined as:

$$N = \varphi\frac{E\lambda}{hc}\left(1 - R_{\mathrm{p}} - T_{\mathrm{p}}\right) \tag{S11}$$

where E is the pump pulse energy, λ is the excitation wavelength, φ is the photon-to-charge branching ratio (the fraction of generated charges per absorbed photon absorbed), and R_p and T_p are the reflectance and transmittance of the sample at the excitation pump wavelength (800 nm, 1.55 eV). Following the definition of photoconductivity (*35*), the charge-carrier mobility μ can be defined as:

$$\mu = \frac{\Delta S\, A_{\mathrm{eff}}}{Ne} \tag{S12}$$

where A_{eff} is the effective overlap area between THz and pump beam, defined in Supplementary Note 1 and e is the elementary charge. Therefore, combining Equation S10 and Equation S11 with Equation S12, the effective (i.e., multiplied by the photon-to-charge branching ratio) electron-hole sum mobility becomes:

$$\varphi\mu = -\epsilon_0 c(n_1 + n_3)\frac{A_{\mathrm{eff}}hc}{eE\lambda(1 - R_{\mathrm{p}} - T_p)}\left(\frac{\Delta T}{T}\right) \quad \text{(S13)}$$

Absence of grain-boundary effects on extracted THz charge-carrier mobilities and implications for OPTP dynamics

The photoconductivity signal obtained by the OPTP technique, which underlies the extracted charge-carrier mobility values, reflects intrinsic intragrain transport. As a result, the THz-derived charge-carrier mobilities for Bi_2S_3 thin films ($\sim 15\ cm^2V^{-1}s^{-1}$) are significantly higher than the long-range DC mobilities extracted from Bi_2S_3-based devices ($\sim 0.01 - 0.001\ cm^2V^{-1}s^{-1}$ for Bi_2S_3-based transistors) (*36*).

Such a selectivity of THz spectroscopy in polycrystalline thin films towards intragrain transport originates from the length scales probed in linear THz spectroscopy. In OPTP measurements, the photogenerated charge carriers are displaced by the oscillating electric field associated with the THz radiation. As discussed in detail by Lloyd-Hughes et al. (*37*), for the low THz-fields analogous to those used in our measurements ($E_{THz} < 10^5\ V\ m^{-1}$), the field-induced charge-carrier excursion is below ~2 nm even for high-mobility semiconductors such as GaAs. Crucially, this excursion length scale is significantly smaller than the size of the Bi_2S_3 grains in our thin films, as shown in Figure S7, thereby implying that THz conductivity measurements probe intrinsic transport largely unaffected by grain boundaries. While THz mobilities are extracted from the peak of the OPTP signal at early sub-ps timescales, we note that the subsequent OPTP dynamics are still generally influenced by defects, surfaces, and interfaces.

For this reason, as described in Supplementary Note 1, we model the fluence-dependent OPTP dynamics as a sum of contributions from a defect-related monomolecular terms (k_1) and a rate constant arising from intrinsic bimolecular recombination (k_2), and are therefore able to differentiate between these contributions.

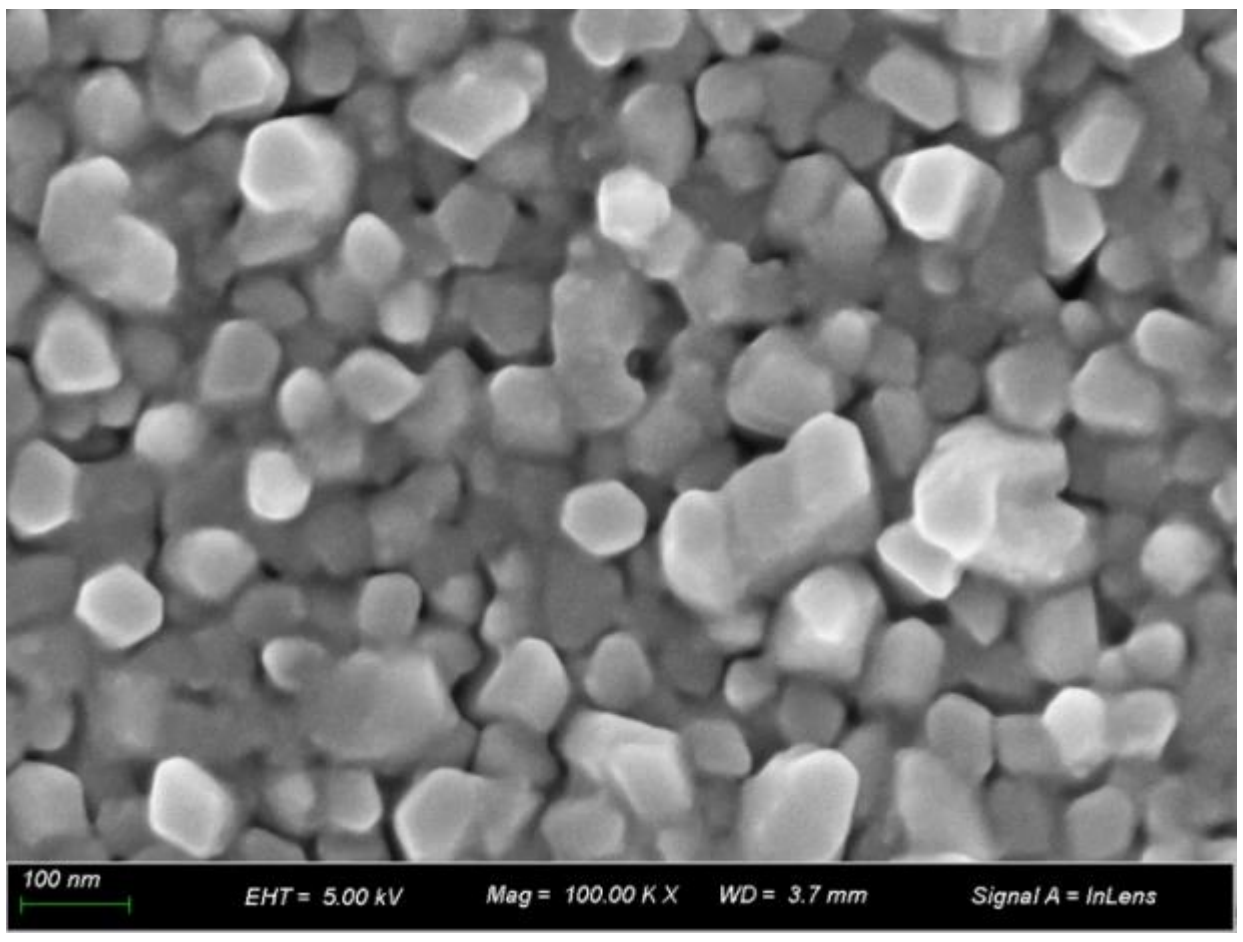

Figure S7 | Surface morphology of Bi_2S_3 thin films. The surface morphology of the samples was characterised using a Zeiss GeminiSEM 500 field-emission scanning electron microscope (SEM). All measurements were performed under high-vacuum conditions at an acceleration voltage of 5 kV and a working distance of 3.7 mm in secondary electron (SE) mode. The samples possess sufficient electrical conductivity to prevent charging artifacts during imaging, and therefore, no metallic coating was applied. SEM micrographs show grain sizes on the order of ~70 - 100 nm.

Our hypothesis is further supported by the THz photoconductivity spectra measured for our Bi_2S_3 thin films. Importantly, several works have reported how nanoscale morphologies (for instance, in nanocrystals and quantum dots (*35, 38*)) can yield backscattering processes that determine a specific phase relation between the oscillating electrons and the THz field, summarised in the Drude-Smith model (*39*). The effect of surfaces is therefore observed mainly in THz photoconductivity spectra as the emergence of a non-zero imaginary part and a non-

monotonous real part of the photoconductivity. Conversely, the THz photoconductivity spectra recorded at 4 K on the photoconductivity peak (Figure S8) exhibit a Drude-like response, i.e. a near-zero imaginary part and a near-constant real part. Such behaviour reflects the classic presence of a free charge-carrier density with low momentum scattering time. Here, the Drude-like behaviour of charge carriers in our Bi_2S_3 thin films shows no evidence of strong grain-boundary scattering effects.

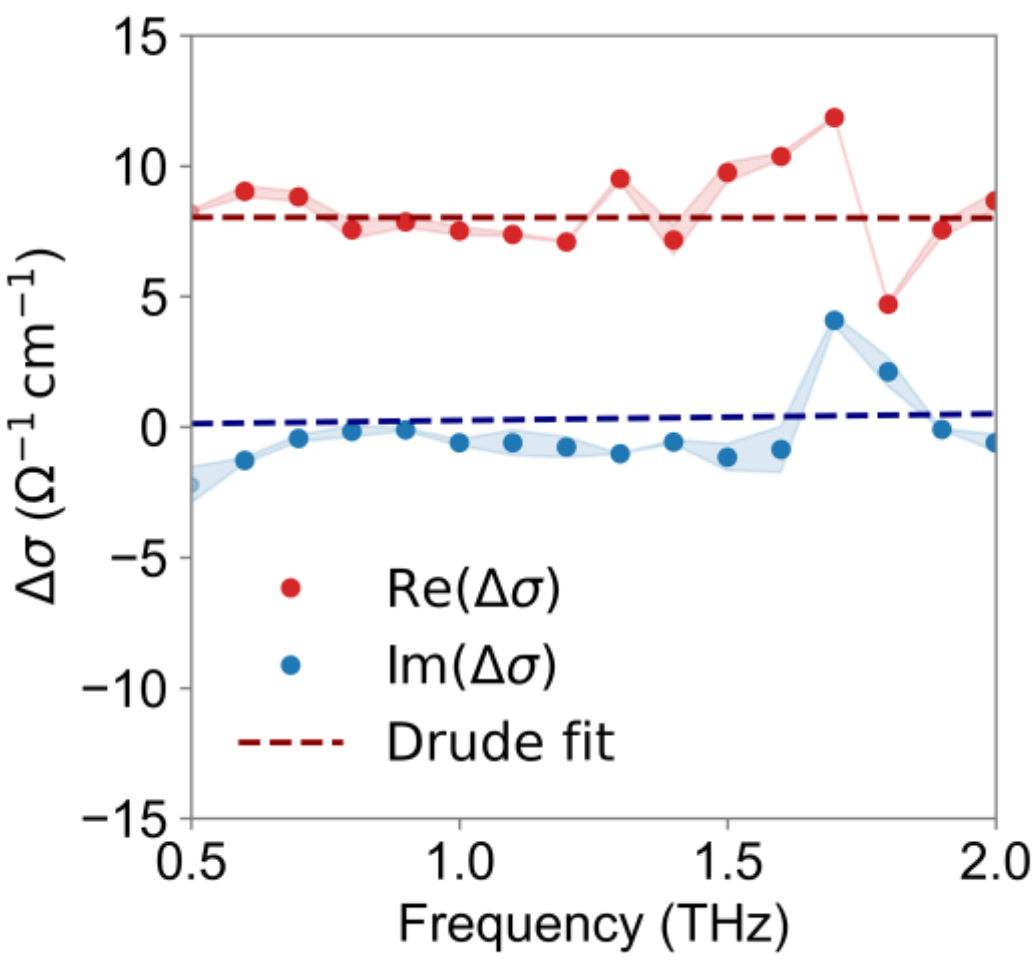

Figure S8 | Photoconductivity spectrum for Bi_2S_3 thin films. Real (red) and imaginary (blue) part of the photoinduced THz conductivity spectra for Bi_2S_3 thin films, measured at 4 K around the peak photoconductivity signal (t = 2 ps) following 1.55 eV excitation at a fluence of 15 μJ cm^{-2}. Dots represent experimental data (with shaded areas indicating statistical error), whereas dashed lines are fits according to the Drude model. We note that the near-zero imaginary part and near constant real part reflect the classic Drude picture for a free density of charge carriers.

Interpretation of temperature-dependent charge-carrier mobility trends

The observed temperature dependence of the charge-carrier mobility in Bi_2S_3 thin films (Figure 3B) is strongly indicative of multiple bands contributing to charge-carrier transport. In

conventional semiconductors, the charge-carrier mobility typically increases with decreasing temperature according to a simple power law $\mu \propto T^{-m}$, where the m exponent is dictated by the predominant phonon coupling mechanism (*40*) – such as Fröhlich or acoustic deformation potential coupling (*41*). While for conventional semiconductors, such terms yield a drastic increase in charge-carrier mobilities – e.g. ~10-fold increase between room temperature (RT) and 4 K for lead halide perovskites (*40, 42*), ~20-fold increase between RT and 77 K for GaAs (*43*), and ~200-fold increase between RT and 10 K for Si(*44*) – we observe < 2-fold increase in charge-carrier mobility for Bi_2S_3 thin films (Figure 3B). The observed mobility behaviour arises from the interplay among multiple bands that contribute to charge-carrier conduction. As shown in Table S4, the calculated higher-lying valence band maxima exhibit a higher effective mass ($m_h^* \sim 2.7, 4.2\ m_0$, respectively) than the lower-lying valence band maximum along the $\Gamma - Z$ direction ($m_h^* \sim 1.3\ m_0$) associated with the direct transition. Therefore, the narrowing of the Fermi-Dirac distribution function at lower temperatures yields a decreasing contribution from the $\Gamma - Z$ maximum, which mitigates the mobility increase related to phonon scattering.

Table S4 | Effective masses of Bi_2S_3 near the band edges. Effective masses of Bi_2S_3 calculated around different points of the Brillouin zone, near the band edges from DFT/PBE + SOC. The mean effective mass for each point is calculated as harmonic mean of the effective mass tensor eigenvalues. Masses are reported in units of the electron rest mass m_0.

**Band-k**	**k (reciprocal lattice units)**	**Charge-carrier type**	**Value (Direction in units of the reciprocal lattice)**			**Harmonic Mean**
**VBM-1**	(1/3, 0, 0)	Hole	1.804 (1, 0, 0)	13.606 (0, 1, 0)	11.662 (0, 0, 1)	4.204
**VBM-2**	(1/3, 0, 1/12)	Hole	1.865 (1, 0, 0.78380)	3.474 (0, 1, 0)	3.893 (-0.76205, 0, 1)	2.775
**CBM**	(0, 0, 1/6)	Electron	0.563 (1, 0, 0)	0.451 (0, 1, 0)	0.751 (0, 0, 1)	0.563
**Direct gap**	(0, 0, 1/5)	Hole	12.599 (1, 0, 0)	1.498 (0, 1, 0)	0.643 (0, 0, 1)	1.303
	(0, 0, 1/5)	Electron	0.618 (1, 0, 0)	0.413 (0, 1, 0)	0.658 (0, 0, 1)	0.540

Supplementary Note 5: Impact of pseudo-direct bandgap character on solar energy conversion devices

The central idea of our work is that the pseudo-direct bandgap character, demonstrated here in Bi_2S_3 thin films, can effectively suppress intrinsic bimolecular recombination (up to 75% in Bi_2S_3) while still retaining a remarkably high absorption coefficient. Both these parameters are key factors in determining the efficiency of solar cells and other solar energy conversion devices (e.g., photoelectrochemical cells). Therefore, we discuss the implications of a pseudo-direct character on these devices, based on three main points:

(i) The charge-carrier lifetime in a semiconductor is generally limited by both trap-mediated monomolecular and intrinsic bimolecular recombination processes(*11*). As described in Supplementary Note 1, monomolecular recombination processes generally dominate in the low charge-carrier density regime. However, in optimised solar absorbers (e.g., silicon, GaAs, lead halide perovskites), the charge-carrier lifetime is mainly limited by bimolecular recombination processes $\tau_{bim} = (nk_2)^{-1}$, as a result of the reduced trap densities (and hence reduced k_1 rates) (*28*). As discussed in Supplementary Note 3, a pseudo-direct semiconductor such as Bi_2S_3 can achieve a ~75% suppression of the bimolecular recombination rate, with respect to a direct semiconductor with the same absorption coefficient α. As shown in Figure S9, the increase in lifetime translates into a longer charge-carrier diffusion length $L_D = \sqrt{D\tau}$ for optimised solar absorbers, which are expected to reduce transport and collection losses (i.e., potentially increasing short-circuit current and fill factor parameters) in devices. We note that, based on the extracted k_1 values for our Bi_2S_3 thin films, the charge-carrier lifetime at solar (AM 1.5) injection levels is dominated by the monomolecular term (see Figure S9). Therefore, this intrinsic advantage is currently latent and would emerge upon defect passivation.

(ii) The suppression of the intrinsic recombination channel also has the potential to reduce fundamental losses in a solar cell. These emission-related losses (which include recombination losses and only partially Boltzmann losses) amount to several per cent, depending on the absorber's bandgap: for example, precise calculations based on the original Shockley-Queisser concept have been made by Hirst et al. (*45*) .

(iii) The extended charge-carrier lifetime (discussed above) carries significant implications for applications beyond photovoltaics. Importantly, long charge-carrier lifetimes are crucial for applications in photocatalysis, where longer lifetimes are necessary to enable time for surface chemical reactions to occur. Importantly, such a scheme trading off photovoltage for longer charge-carrier lifetimes is already adopted in nature: biological photosystems sacrifice part of their photovoltage to achieve gains in excited state lifetimes, as explained in Ref.(*46*) .

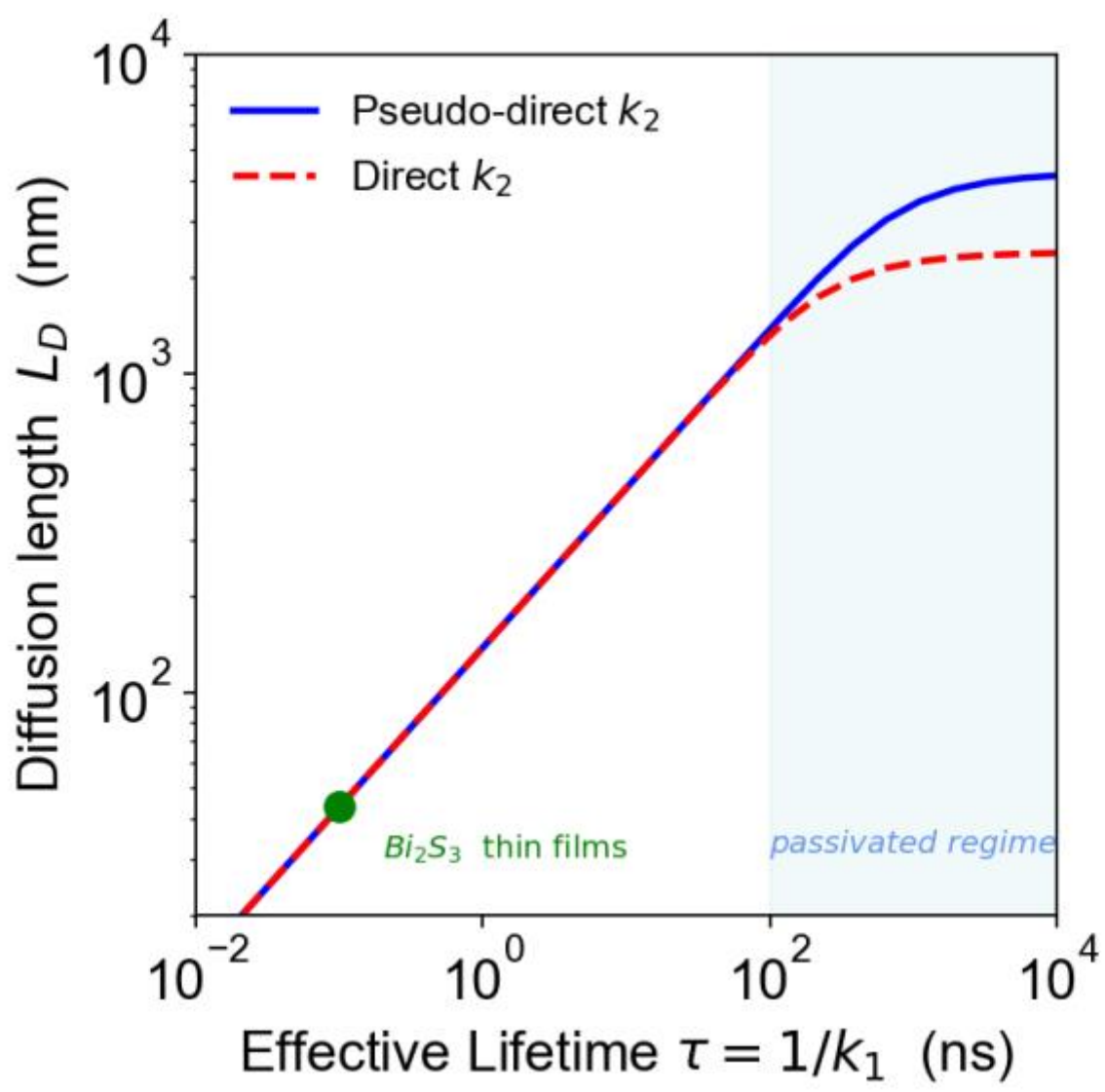

Figure S9 | Diffusion length calculations for Bi_2S_3 thin films. Calculated charge-carrier diffusion length as a function of the effective charge-carrier lifetime for a pseudo-direct bandgap character (blue solid line) and for a standard direct bandgap character (red dashed line). The green dot indicates the

obtained values for k_1 and k_2 experimentally obtained for Bi_2S_3 films examined in this study. The k_2 value for the pseudo-direct line is value of $k_{2,Bi2S3} = 5 \times 10^{-11}$ cm^3 s^{-1} experimentally determined at 300 K, while for the direct line, we use the extracted 75% suppression, i.e. $k_{2,\mathrm{direct}} = 4 \times k_{2,\mathrm{pseudodirect}}$. The comparison highlights that the impact of a pseudo-direct bandgap character will only become apparent in the radiative regime (blue shading) for a well-passivated material.

Supplementary References

1. M. S. Hybertsen, S. G. Louie, Electron correlation in semiconductors and insulators: Band gaps and quasiparticle energies. *Physical Review B* **34**, 5390–5413 (1986).
2. J. Deslippe *et al.*, BerkeleyGW: A massively parallel computer package for the calculation of the quasiparticle and optical properties of materials and nanostructures. *Computer Physics Communications* **183**, 1269–1289 (2012).
3. B. A. Barker *et al.*, Spinor GW/Bethe-Salpeter calculations in BerkeleyGW: Implementation, symmetries, benchmarking, and performance. *Physical Review B* **106**, 115127 (2022).
4. R. W. Godby, R. J. Needs, Metal-insulator transition in Kohn-Sham theory and quasiparticle theory. *Physical Review Letters* **62**, 1169–1172 (1989).
5. M. R. Filip, C. E. Patrick, F. Giustino, *GW* quasiparticle band structures of stibnite, antimonselite, bismuthinite, and guanajuatite. *Physical Review B* **87**, 205125 (2013).
6. M. Rohlfing, S. G. Louie, Electron-Hole Excitations in Semiconductors and Insulators. *Physical Review Letters* **81**, 2312–2315 (1998).
7. F. Giustino, *Materials Modelling Using Density Functional Theory: Properties and Predictions; Chapter 10, Eq. 10.53*. (Oxford University Press, 2014).
8. L. M. Herz, Charge-Carrier Dynamics in Organic-Inorganic Metal Halide Perovskites. *Annual Review of Physical Chemistry* **67**, 65–89 (2016).
9. T. C. Sum, M. Righetto, S. S. Lim, Quo vadis, perovskite emitters? *The Journal of Chemical Physics* **152**, 130901 (2020).
10. A. Kiligaridis *et al.*, Are Shockley-Read-Hall and ABC models valid for lead halide perovskites? *Nature Communications* **12**, 3329 (2021).
11. M. B. Johnston, L. M. Herz, Hybrid Perovskites for Photovoltaics: Charge-Carrier Recombination, Diffusion, and Radiative Efficiencies. *Accounts of Chemical Research* **49**, 146–154 (2016).
12. G. Zatryb, A. Podhorodecki, J. Misiewicz, J. Cardin, F. Gourbilleau, On the nature of the stretched exponential photoluminescence decay for silicon nanocrystals. *Nanoscale Research Letters* **6**, 106 (2011).
13. M. Taddei *et al.*, Interpreting Halide Perovskite Semiconductor Photoluminescence Kinetics. *ACS Energy Letters* **9**, 2508–2516 (2024).
14. A. D. Wright *et al.*, Band-Tail Recombination in Hybrid Lead Iodide Perovskite. *Advanced Functional Materials* **27**, 1700860 (2017).
15. M. N. Berberan-Santos, B. Valeur, Luminescence decays with underlying distributions: General properties and analysis with mathematical functions. *Journal of Luminescence* **126**, 263–272 (2007).
16. W. Rehman *et al.*, Charge-Carrier Dynamics and Mobilities in Formamidinium Lead Mixed-Halide Perovskites. *Advanced Materials* **27**, 7938–7944 (2015).

17. C. Q. Xia *et al.*, Limits to Electrical Mobility in Lead-Halide Perovskite Semiconductors. *The Journal of Physical Chemistry Letters* **12**, 3607–3617 (2021).
18. M. J. Trimpl *et al.*, Charge-Carrier Trapping and Radiative Recombination in Metal Halide Perovskite Semiconductors. *Advanced Functional Materials* **30**, 2004312 (2020).
19. R. K. Ahrenkiel, B. M. Keyes, D. J. Dunlavy, Intensity-dependent minority-carrier lifetime in III-V semiconductors due to saturation of recombination centers. *Journal of Applied Physics* **70**, 225–231 (1991).
20. S. D. Stranks *et al.*, Recombination Kinetics in Organic-Inorganic Perovskites: Excitons, Free Charge, and Subgap States. *Physical Review Applied* **2**, 034007 (2014).
21. V. Sarritzu *et al.*, Direct or Indirect Bandgap in Hybrid Lead Halide Perovskites? *Advanced Optical Materials* **6**, 1701254 (2018).
22. A. M. Ulatowski *et al.*, Contrasting Charge-Carrier Dynamics across Key Metal-Halide Perovskite Compositions through In Situ Simultaneous Probes. *Advanced Functional Materials* **33**, 2305283 (2023).
23. J. M. Richter *et al.*, Direct Bandgap Behavior in Rashba-Type Metal Halide Perovskites. *Advanced Materials* **30**, 1803379 (2018).
24. M. Kaiser *et al.*, Interpreting the Time-Resolved Photoluminescence of Quasi-2D Perovskites. *Advanced Materials Interfaces* **8**, 2101326 (2021).
25. P. Wurfel, The chemical potential of radiation. *Journal of Physics C: Solid State Physics* **15**, 3967 (1982).
26. P. Würfel, S. Finkbeiner, E. Daub, Generalized Planck's radiation law for luminescence via indirect transitions. *Applied Physics A* **60**, 67–70 (1995).
27. W. van Roosbroeck, W. Shockley, Photon-Radiative Recombination of Electrons and Holes in Germanium. *Physical Review* **94**, 1558–1560 (1954).
28. C. L. Davies *et al.*, Bimolecular recombination in methylammonium lead triiodide perovskite is an inverse absorption process. *Nature Communications* **9**, 293 (2018).
29. H. T. Nguyen, S. C. Baker-Finch, D. Macdonald, Temperature dependence of the radiative recombination coefficient in crystalline silicon from spectral photoluminescence. *Applied Physics Letters* **104**, 112105 (2014).
30. T. Kirchartz, U. Rau, Decreasing Radiative Recombination Coefficients via an Indirect Band Gap in Lead Halide Perovskites. *The Journal of Physical Chemistry Letters* **8**, 1265–1271 (2017).
31. R. Wallauer, J. Reimann, N. Armbrust, J. Güdde, U. Höfer, Intervalley scattering in MoS2 imaged by two-photon photoemission with a high-harmonic probe. *Applied Physics Letters* **109**, 162102 (2016).
32. C. Wehrenfennig, G. E. Eperon, M. B. Johnston, H. J. Snaith, L. M. Herz, High Charge Carrier Mobilities and Lifetimes in Organolead Trihalide Perovskites. *Advanced Materials* **26**, 1584–1589 (2014).
33. H. J. Joyce *et al.*, A review of the electrical properties of semiconductor nanowires: insights gained from terahertz conductivity spectroscopy. **31**, 103003 (2016).
34. C. L. Davies, J. B. Patel, C. Q. Xia, L. M. Herz, M. B. Johnston, Temperature-Dependent Refractive Index of Quartz at Terahertz Frequencies. *Journal of Infrared, Millimeter, and Terahertz Waves* **39**, 1236–1248 (2018).
35. M. Righetto *et al.*, Cation-Disorder Engineering Promotes Efficient Charge-Carrier Transport in $AgBiS_2$ Nanocrystal Films. *Advanced Materials* **35**, 2305009 (2023).
36. X. Fang *et al.*, Solution-processed bismuth sulfide incorporated with zinc for high-gain and low-noise photodetection. *Applied Physics Letters* **122**, (2023).
37. J. Lloyd-Hughes, T.-I. Jeon, A Review of the Terahertz Conductivity of Bulk and Nano-Materials. *Journal of Infrared, Millimeter, and Terahertz Waves* **33**, 871–925 (2012).

38. S. G. Motti *et al.*, CsPbBr3 Nanocrystal Films: Deviations from Bulk Vibrational and Optoelectronic Properties. *Advanced Functional Materials* **30**, 1909904 (2020).
39. T. L. Cocker *et al.*, Microscopic origin of the Drude-Smith model. *Physical Review B* **96**, 205439 (2017).
40. L. M. Herz, Charge-Carrier Mobilities in Metal Halide Perovskites: Fundamental Mechanisms and Limits. *ACS Energy Letters* **2**, 1539–1548 (2017).
41. L. R. V. Buizza, L. M. Herz, Polarons and Charge Localization in Metal-Halide Semiconductors for Photovoltaic and Light-Emitting Devices. *Advanced Materials* **33**, 2007057 (2021).
42. R. L. Milot, G. E. Eperon, H. J. Snaith, M. B. Johnston, L. M. Herz, Temperature-Dependent Charge-Carrier Dynamics in $CH_3NH_3PbI_3$ Perovskite Thin Films. *Advanced Functional Materials* **25**, 6218–6227 (2015).
43. G. E. Stillman, C. M. Wolfe, J. O. Dimmock, Hall coefficient factor for polar mode scattering in n-type GaAs. *Journal of Physics and Chemistry of Solids* **31**, 1199–1204 (1970).
44. C. Jacoboni, C. Canali, G. Ottaviani, A. Alberigi Quaranta, A review of some charge transport properties of silicon. *Solid-State Electronics* **20**, 77–89 (1977).
45. L. C. Hirst, N. J. Ekins-Daukes, Fundamental losses in solar cells. *Progress in Photovoltaics: Research and Applications* **19**, 286–293 (2011).
46. E. Pastor *et al.*, Electronic defects in metal oxide photocatalysts. *Nature Reviews Materials* **7**, 503–521 (2022).
