# Supplementary material for: Delayed electron–hole recombination in a pseudo-direct semiconductor
Source: Nature. 2026 Oct 28;659(8139):76–82. doi: 10.1038/s41586-026-11223-x (PMC13736635; doi:10.1038/s41586-026-11223-x)
Supplement: Supplementary file 2 [file 41586_2026_11223_MOESM2_ESM.pdf]

nature portfolio

Peer Review File

Delayed Electron-Hole Recombination in a Pseudo-Direct Semiconductor

Corresponding Author: Professor Laura Herz

Any redactions in this file are there to maintain patient confidentiality, the confidentiality of unpublished data, or to remove third-party material.

This file contains all reviewer reports in order by version, followed by all author rebuttals in order by version.

Version 0:

Reviewer comments:

Referee #1

(Remarks to the Author)
This manuscript presents a comprehensive study of charge-carrier recombination dynamics in Bi2S3 thin films. The authors introduce the concept of pseudo-direct semiconductors, in which a high absorption coefficient coexists with a markedly suppressed bimolecular recombination rate. This behaviour is attributed to a mixed direct–indirect bandgap structure with an energy separation of approximately 40 meV, allowing light absorption and recombination to be effectively decoupled. The proposed concept challenges conventional optical reciprocity principles and, if generalizable, could offer a new materials design strategy for solar energy conversion.
The work is of broad interest to the fields of semiconductor physics, photovoltaics and photocatalysis. However, several aspects require clarification or additional support should be considered. These are outlined below.

Major Comments
1. Limited generalizability of the proposed concept:
The title is “Delayed Electron-Hole Recombination in a Pseudo-Direct Bandgap Semiconductor”, this article mainly talks about Bi2S3, the indirect transition defines pseudo-direct semiconductor. Authors mentioned other materials like dichacogenides, indium selenide and phosphorene possessing similar characters, and propose that the effect is caused by layered nature, why not have a deeper research in here?
2. Lack of experimental support
In Discussion (Page 18), the authors state that “charge-carrier recombination lifetimes in such pseudo-direct semiconductors can potentially be extended by up to two orders of magnitude …,” citing simulation results in Figure S14. While this simulation is informative, is there any experimental evidence or existing examples to support this?

Minor Comments
1. Language and Grammar:
Page 4, Lines 1-5: "However, the associated..." This sentence might be a bit long. Consider splitting it for improved readability?
2. Tables in SI consecutiveness
Why is there only Table 2 in the Supplementary Information but no Table 1?
3. Lack of experimental support
The authors report DFT-calculated indirect bandgap 2.0 eV and direct bandgap 2.04 eV. Although it might be indirectly proved in other figures, but a direct experimental verification would be more convincing.

Conclusion
This is an excellent piece of work that introduces a significant new concept to the field. The experimental data is robust, and the theoretical modelling supports the conclusions well. The only poential gap is that all the conclusions come from Bi2S3. As a result, due to it’s universality, I recommend rejection.
Given the strength of the experimental data on Bi2S3 and the relevance to nanostructured materials, the work would be well suited to the scope of Nature Materials, which regularly publishes studies on the fundamental properties of emerging nanomaterials.

Referee #2

(Remarks to the Author)
In this study, Righetto et al. propose a compelling "pseudo-direct" semiconductor model to decouple light absorption from charge-carrier recombination, aiming to circumvent the fundamental constraints of the van Roosbroeck-Shockley (vRS) relation. This work is highly relevant and timely, as the optical reciprocity principle, where efficient absorbers are inherently efficient emitters, often imposes a ceiling on the open-circuit voltage (Voc) and limits carrier lifetimes in photovoltaic and photoelectrochemical devices. The authors focus on the unique electronic structure of Bi2S3, where the presence of multiple indirect transition states at energy levels nearly degenerate with the direct gap offers a potential pathway to suppress intrinsic recombination without compromising absorption. The authors categorize Bi2S3 as a direct semiconductor given its high absorption coefficient (alpha > 10^5 cm^-1), yet they acknowledge an absorption profile that mimics indirect semiconductors. Through JDOS analysis and low-temperature absorption measurements, they argue that the observed optical behavior is dominated by direct transitions, despite a calculated indirect gap that is approximately 40 meV lower. The THz conductivity measurements provide experimental evidence for this decoupling; Bi2S3 exhibits a significantly suppressed bimolecular recombination coefficient (k2), suggesting that carriers relax into a transient "reservoir" of indirect states. The concept is timely and potentially significant. However, in its current form the manuscript contains several experimental and theoretical gaps that must be addressed before it meets the high standard required for Nature.

1. The central mechanistic claim — that photoexcited carriers scatter from the direct Γ valley into the indirect reservoir states — is supported only by macroscopic observables. While the suppressed k2 and the THz conductivity data are consistent with this picture, they do not constitute momentum-resolved proof of the proposed population transfer. Time-resolved ARPES (TR-ARPES) would provide direct, valley-selective tracking of carrier dynamics and would substantially strengthen the mechanistic interpretation. Without such data, the intervalley scattering pathway remains an assumption, and alternative explanations for the suppressed k_2 cannot be excluded.

2. The manuscript references Figure S16 in connection with inter-ribbon interactions and their influence on the electronic structure, but this figure does not appear in the current version of the Supporting Information. This data should be provided, as the role of structural coupling between ribbons is directly relevant to the computed band structure and, by extension, to the ΔE value on which the entire pseudo-direct framework rests.

3. Several aspects of the DFT calculations require clarification. Regarding the computational methodology, the authors should specify the energy and force convergence criteria for structural optimization. Furthermore, please clarify how the Pulay stress was managed during the variable-cell relaxation (vc-relax) process, as it can significantly affect the lattice parameters in such systems.

4. Importantly, the omission of van der Waals (vdW) corrections is a significant concern. For a quasi-1D material like Bi2S3, where inter-ribbon distances are governed by dispersion forces, the lack of a vdW scheme (e.g., DFT-D3) could lead to inaccurate lattice parameters. Given the extreme sensitivity of the Delta E (~40 meV) offset to the structural environment, it is imperative to verify if these energy levels remain consistent when vdW interactions are properly accounted for.
5. All measurements are performed on thermally evaporated polycrystalline films. This is a significant confound, because grain boundaries and structural disorder are independently known to produce weak temperature dependence of carrier mobility, elevated monomolecular recombination rates, and broadened absorption onsets — precisely the signatures that the authors invoke as evidence for the pseudo-direct mechanism. As it stands, it is not possible to unambiguously distinguish intrinsic band-structure effects from extrinsic disorder-related phenomena. Reproducing the key observations on structurally well-defined material — for example, epitaxial films grown on lattice-matched substrates — would be an important step towards establishing that the reported behaviour is intrinsic.

6. Closely related to the above is the question of defect chemistry. Sulfur vacancies are thermodynamically favourable under typical chalcogenide growth conditions and are expected to introduce states within the bandgap. The stretched-exponential trapping behaviour and the large monomolecular recombination component k1 observed in the present films are both consistent with a significant density of such defect states. Under these circumstances, the suppression of k2 may reflect, at least in part, carrier trapping and localisation rather than solely the intrinsic pseudo-direct band structure. The manuscript would benefit from a more explicit characterisation or discussion of defect densities and their electronic impact — for instance, through sub-bandgap spectroscopy or temperature-dependent admittance measurements — to delineate the relative contributions of intrinsic and extrinsic processes.

7. While the manuscript highlights potential implications for solar energy conversion, no device-level validation is provided. Demonstrating the feasibility of this concept in an actual solar cell device, or at least providing a quantitative estimate of the expected gain in quasi-Fermi level splitting or open-circuit voltage, would greatly strengthen the relevance of the work.

Minor Comments
1. Terminology: For academic precision, "conduction band bottom" and "valence band top" should be replaced with Conduction Band Minimum (CBM) and Valence Band Maximum (VBM).
2. Clarification of Extremas: On page 14, the discussion of "valence band minima and maxima" should be clarified to specify whether the authors are referring to higher-lying or lower-lying sub-bands.

Referee #3

(Remarks to the Author)
This manuscript investigates how the optical reciprocity constraint, rooted in Planck's law and the detailed balance limit, may be mitigated in semiconductors. Using Bi_2S_3 as a model system, the authors demonstrate a pseudo-direct bandgap with closely spaced indirect and direct transitions, enabling strong absorption while suppressing bimolecular recombination. This decoupling of absorption and recombination suggests a potential pathway toward improved solar energy conversion beyond conventional limits. Overall, the work presents sufficient novelty and depth to meet the standards of Nature. I therefore recommend acceptance of the manuscript subject to minor revisions.
1. The authors calculated the joint density of states and electronic band structure of Bi2S3, identifying a lowest-energy direct transition of ~40 meV. This conclusion is indirectly supported by the charge-carrier recombination dynamics in Fig. 3. More direct experimental evidence would be beneficial to quantitatively determine the magnitude of this energy difference.
2. In Fig. 3c, the authors discuss the effect of the valence band maximum energy difference (ΔE) on the bimolecular intrinsic recombination process (k2). However, it is unclear whether the momentum difference (Δk) between the valence band maxima also influences the carrier dynamics. I recommend adding a discussion on this aspect.
3. The authors mention that Bi2S3 has significant potential for photoelectric applications and compare its k2 values and absorption coefficient with those of common photovoltaic materials. However, as shown in Fig. 3b, the carrier mobility of Bi2S3 at room temperature is quite low, which would limit its carrier diffusion length. It is recommended that the authors fabricate a prototype device based on Bi2S3 to experimentally validate its potential applications in the optoelectronic field.
4. The samples are thermally evaporated polycrystalline films. While the authors acknowledge dispersive trapping and selective carrier trapping, the possibility remains that grain-boundary or surface effects contribute to the observed k_2 suppression or to the mobility temperature dependence. A brief comment (or SI section) comparing to literature single-crystal or colloidal Bi_2S_3 data (or noting why the intrinsic band-structure picture still dominates) would strengthen the claim.
5. The abstract promises "far-reaching implications for solar energy conversion." The discussion quantifies the ~75 % k2 suppression but does not estimate the resulting gain in detailed-balance efficiency or carrier lifetime under 1-sun illumination. A short paragraph or SI calculation (e.g., using the measured absorption spectrum + reduced k2 in a drift-diffusion or SQ-like model) would make the applied impact concrete.

Referee #4

(Remarks to the Author)
In their manuscript entitled "Delayed Electron-Hole Recombination in a Pseudo-Direct Bandgap Semiconductor", Righetto et al. discuss the balance between light emission and absorption in the semiconductor Bi2S3 that features an indirect band gap just 40 meV below its direct gap. Since direct-gap absorption and indirect-gap emission after carrier thermalization do not involve the same electronic states, the two are not constrained by Kirchhoff's law. The authors argue that the combination of efficient direct-gap absorption and delayed indirect-gap emission is a beneficial property for future photovoltaic materials to circumvent the Shockley-Queisser efficiency limit. They combine first-principles density-functional theory simulations with luminescence and conductivity experiments as well as carrier-dynamics simulations to substantiate their claims.

As a theorist, I will mainly comment on the theory sections of the present study. The manuscript is physically sound and very well written. The argumentation is conclusive, sheds light on the controversial band-edge physics of Bi2S3, and suggests a convincing mechanism to explain the available literature data as well as the experiments conducted in this work. Due to its potential wider implications for more efficient future photovoltaics, it is of interest to a wide community, and I can recommend publication in Nature provided that the following issues are addressed:

* In the discussion, the authors claim the charge-carrier recombination mechanism to be "unique" to Bi2S3 thin films. In principle, it should be a quite abundant property present in a number of semiconductors. DFT databases like the Materials Project contain millions of data sets including band-gap data on semiconductors. Shouldn't it be easy to extract other candidate materials with similar band-gap properties as Bi2S3 and identify those with comparable optical properties?

* What do the authors consider a good "threshold" to distinguish between an indirect and a pseudo-direct semiconductor at room temperature? In the manuscript, indirect-direct-gap energy differences ranging up to 7 k_B*T at room temperature are discussed. Would, for instance, germanium with a 0.14 eV gap difference still count as pseudo-direct here (e.g. considering the data in Figs. 3C and S14), even though it is usually considered indirect?

* The manuscript never mentions the suitability of the Bi2S3 band gap for photovoltaics.

* Multiple band extrema lying close in energy often come along with high effective masses as evident from Table S3. How do these high effective masses impact the carrier extraction properties in a photovoltaic context?

* Figure 1D shows the joint density of states as a measure for the absorption properties of the material. However, this can be highly misleading as optical-matrix-element effects are disregarded. Dipole-forbidden excitations would show in the joint density of states, but not contribute to absorption. The authors should present the absorption spectrum instead of the joint density of states, which is standard and doable with negligible additional computational cost.

* The position of the direct-gap transition along Gamma-Z in the Brillouin zone is confusing: According to the main text (p. 7), it is located at 0.5*pi/c. According to Figs. 1E,F and S4, it is located at 0.2*2*pi/c = 0.4*pi/c. And according to Table S2, it is located at 0.33*pi/c, provided that the "PBE (dense grid)" setting is the relevant one which is stated nowhere but seems to be the case, as the data for this grid have apparently been used for the conduction-band minimum and the direct-indirect-gap

energy difference. How many grid points (not irreducible points) were used for the dense grid? Further, the statement "the valence band top (Figure 1F) is found near the Gamma-U direction ..." (p. 7) is in contradiction to the given coordinates and Fig. 1F where the valence-band maximum is rather close to Gamma-X.

Manuscript preparation issues:

* Undefined abbreviations: LHP (p. 3), RT (p. 13), FD (legend of Fig. S13C, D).

* It may be a matter of personal taste, but I consider Fig. S2 much more insightful than Fig. 1B.

* Caption of Fig. 1E, F: There is no BZ path, but a plane in the BZ shown in the figure. There is also no yellow cross in the figure, rather something like an angle.

* The y-axis label in Fig. 2B, C, D is not explained.

* "... the higher-lying valence band minima ..." (p. 14) should probably read "valence band maxima".

* In the caption of Fig. 3A, it reads "a two valence bands".

* Missing units for k coordinates in Table S3.

* In Eq. (S6), k_b should read k_B.

* Below Eq. (S2), it reads "Here i is the recombination order" even though there is no i.

Referee #5

(Remarks to the Author)
I co-reviewed this manuscript with one of the reviewers who provided the listed reports.

Version 1:

Reviewer comments:

Referee #1

(Remarks to the Author)
Compared with the initial version, the revised manuscript is more logically coherent, and the explanations of the physical mechanism are clearer and more convincing. The concept of a pseudo-direct bandgap semiconductor, in which strong direct-like optical absorption is decoupled from suppressed bimolecular recombination, remains intriguing and potentially significant for both semiconductor physics and photovoltaics. Regarding the presentation of error bars and statistical information, I have examined the relevant figures (Figure 3B, Figure 3D in the main text, and Figure S13 in the Supplementary Information). In all cases, the error bars are appropriately defined in the corresponding figure legends and represent uncertainties arising from experimental reproducibility and data processing. Nevertheless, several issues remain that should be addressed.
1.One issue throughout the manuscript is the use of several different quantitative metrics to describe how much bimolecular recombination is “suppressed”, yet these metrics are not clearly differentiated. The vRS model predicts ~75% suppression (i.e., k2 is 4 times lower) at room temperature for ΔE = 40 meV, relative to a hypothetical direct semiconductor with the same direct-gap properties. This is stated in the Discussion. However, the authors also compare the experimental k2 of Bi2S3 (~5 × 10-11 cm3 s-1) with those of lead halide perovskites (6–12 × 10-10 cm3 s-1) and GaAs (~7 × 10-10 cm3 s-1), implying 12–24-fold and 14-fold differences, respectively. These are cross-material comparisons and do not reflect a suppression factor within Bi2S3 itself. In addition, the Discussion states that lifetimes can be extended by “up to two orders of magnitude” for pseudo-direct semiconductors in general (with larger ΔE < 100 meV), not specifically for Bi2S3. Given the coexistence of these three distinct frames of reference, the authors should explicitly clarify what each value represents and avoid conflating them. A concise summary table or a dedicated explanatory paragraph would help readers distinguish these metrics.
2.The authors have substantially expanded the Discussion to address generalizability and have outlined a two-step high-throughput screening strategy using ΔE as a descriptor. This is helpful and well-reasoned. However, the discussion would be considerably strengthened if the authors could provide even a preliminary quantitative comparison with known layered materials that have been proposed to exhibit similar pseudo-direct character.

Referee #2

(Remarks to the Author)
The authors have made a substantial effort to address my previous concerns, including the addition of SEM characterization, frequency-resolved THz conductivity data, expanded kinetic analysis, and further discussion of the implications for solar-energy conversion. These additions improve the manuscript. However, I do not consider some comments were sufficiently resolved.

Regarding comment 5: The added SEM image (Figure S16) and THz photoconductivity spectrum (Figure S17) support the conclusion that the measured THz mobility predominantly reflects intragrain transport and that strong grain-boundary backscattering is not evident within the measured THz response. This substantially addresses the transport-related concern. However, supplementary note 4 extends this conclusion to intrinsic intragrain transport and recombination dynamics and states that the Drude-like response confirms the absence of grain-boundary-induced effects. Figures S16 and S17 directly support the intragrain nature of the THz transport, but they do not by themselves establish that the recombination dynamics or broadened absorption onset are unaffected by structural disorder, defects, surfaces, or interfaces. Please restrict the conclusion to what is directly supported by Figures S16 and S17 and explicitly acknowledge that possible microstructural effects on recombination and optical broadening cannot be fully excluded.

Regarding comment 6: The expanded analysis in Supplementary Note 1 appropriately explains that the faster decay at higher fluence in Figures 2B and 2D is inconsistent with a simple trap-saturation scenario. This supports the presence of a substantial second-order recombination contribution. Nevertheless, the statements that disorder- and trap-mediated processes cannot reproduce the observations and that the data unambiguously identify intrinsic bimolecular recombination are too categorical. The manuscript itself shows significant selective trapping and dispersive monomolecular recombination in Figure 2A and Figure S7. Please revise these statements to specify that the data exclude a simple trap-saturation explanation, rather than all possible defect-coupled kinetics. The authors should also clarify whether the fitted k2 is intended as a microscopic band-to-band recombination coefficient or an effective second-order coefficient extracted from the mobile-carrier THz signal.

Regarding comment 7: Supplementary Note 5 and Figure S19 provide a useful discussion of carrier lifetime and diffusion length. The authors also appropriately acknowledge that, in the present Bi2S3 films, recombination under solar injection conditions remains dominated by the monomolecular term. However, my original comment 7 specifically requested either device-level validation or a quantitative estimate of the expected quasi-Fermi-level splitting or open-circuit voltage. Figure S19 presents a diffusion-length analysis but does not provide either quantity. Therefore, this part of the comment remains unresolved. Please provide a quantitative estimate of the quasi-Fermi-level splitting or radiative Voc for the pseudo-direct and corresponding direct-gap cases, including the effect of delta E. Alternatively, the manuscript should moderate claims concerning device-efficiency gains and clearly state that the voltage and efficiency benefits remain prospective rather than demonstrated in the present Bi2S3 films.

Referee #3

(Remarks to the Author)
The revised manuscript has been thoroughly improved in accordance with the reviewers' feedback. The authors have adequately clarified the previous points of concern, and the added discussions are satisfactory. Consequently, I consider the manuscript well-suited for publication and recommend its acceptance.

Referee #4

(Remarks to the Author)
The authors have addressed all criticisms thoroughly and amended and extended the manuscript with further discussions where appropriate. I consider the manuscript now suitable for publication in Nature.

I would just like to raise a minor point regarding the newly added GW/BSE calculations for the optical absorption. First of all, the calculated absorption spectrum matches the measured absorption data very well, much better than the joint density of states from the original version of the manuscript. However, the convergence study for the GW corrections (see Fig. S1 and first line in Tab. S1) was performed with a technically not suitable k-point grid. As evidenced from the band structure in Fig. 1B, the Brillouin zone extension in y direction is about twice as long as in x and z direction. Therefore, the y direction should be sampled with twice as many k points as the x and z direction to ensure a homogeneous sampling of the reciprocal space. For unexplained reasons, the authors have used a set of k-point grids that fulfills this condition (see all but the first line of Tab. S1) and then chose to add the inappropriate 2x2x4 grid and conduct their GW convergence for this very grid. I acknowledge that this will not change the conclusions of the manuscript, since a PBE+G0W0 calculation with a plasmon-pole model is anyway not expected to reach experimental gap values (as the authors mention correctly) and the exact gap value is not a major point of discussion. However, I also do not want to leave a technically not sound convergence study uncommented.

Manuscript preparation issues:

* Figs. 1E, F still contain the old abbreviations CBB and VBT instead of CBM and VBM. Likewise, in the caption, there is still reference to the Y point of the Brillouin zone that is actually not depicted in the plane shown in the figures.

* Caption of Fig. S3: The blue dashed line indicates the highest - not the lowest - occupied energy level.

Referee #5

(Remarks to the Author)
I co-reviewed this manuscript with one of the reviewers who provided the listed reports.

Version 2:

Reviewer comments:

Referee #1

(Remarks to the Author)
The authors have made efforts in addressing all remaining concerns raised in the previous round of review. I have carefully examined their point-by-point reply and the revised manuscript, and I am satisfied with the changes implemented. All editorial requirements have been fulfilled. The scientific content of the manuscript has been further improved in terms of clarity, precision, and scope. I consider the manuscript in its present form to be suitable for publication in Nature and recommend its acceptance.

Referee #2

(Remarks to the Author)
The authors have satisfactorily addressed comments 5 and 6.

On comment 7, I think the revised, more cautious wording on device-efficiency gains is sufficient. I am still not convinced by the statement that a reciprocity-based radiative-limit Voc estimate would be "not meaningful". In principle, such an estimate should still be possible within the same generalized Planck/detailed-balance framework used in Supplementary Note 3. I would suggest that the authors either clarify this point or add a brief estimate in the SI.

This is a minor point and would not prevent publication. I am otherwise satisfied with the revision and support publication.

Referee #4

(Remarks to the Author)
Fine for me.

Referee #5

(Remarks to the Author)
I co-reviewed this manuscript with one of the reviewers who provided the listed reports.

Open Access This Peer Review File is licensed under a Creative Commons Attribution 4.0 International License, which permits use, sharing, adaptation, distribution and reproduction in any medium or format, as long as you give appropriate credit to the original author(s) and the source, provide a link to the Creative Commons license, and indicate if changes were made.
In cases where reviewers are anonymous, credit should be given to 'Anonymous Referee' and the source.
The images or other third party material in this Peer Review File are included in the article's Creative Commons license, unless indicated otherwise in a credit line to the material. If material is not included in the article's Creative Commons license and your intended use is not permitted by statutory regulation or exceeds the permitted use, you will need to obtain

permission directly from the copyright holder.
To view a copy of this license, visit https://creativecommons.org/licenses/by/4.0/

Response to Reviewers of Nature Manuscript 2026-03-06751 "Delayed Electron-Hole Recombination in a Pseudo-Direct Bandgap Semiconductor" by Righetto et al.

We thank all Reviewers for their very positive overall appraisal of our study, their careful reading of our manuscript and for their thoughtful and constructive comments. Their feedback has helped us significantly improve the clarity, scope, and presentation of the work. We have carefully addressed all reviewer points and hope that you may now find the manuscript suitable for publication.

Below, reviewers' comments are given in *green italics*, our responses are given in black, and our highlighted revisions in the manuscript are given in *blue italics*. Deleted parts are given in ~~*brown italics*~~.

Reviewer #1

This manuscript presents a comprehensive study of charge-carrier recombination dynamics in Bi2S3 thin films. The authors introduce the concept of pseudo-direct semiconductors, in which a high absorption coefficient coexists with a markedly suppressed bimolecular recombination rate. This behaviour is attributed to a mixed direct–indirect bandgap structure with an energy separation of approximately 40 meV, allowing light absorption and recombination to be effectively decoupled. The proposed concept challenges conventional optical reciprocity principles and, if generalizable, could offer a new materials design strategy for solar energy conversion.

The work is of broad interest to the fields of semiconductor physics, photovoltaics and photocatalysis. However, several aspects require clarification or additional support should be considered. These are outlined below.

We thank the Reviewer for their interest in our work and for recognising the broad impact of this discovery, which adds a new dimension to materials design for solar energy conversion.

Major Comments

1. Limited generalizability of the proposed concept:

The title is "Delayed Electron-Hole Recombination in a Pseudo-Direct Bandgap Semiconductor", this article mainly talks about Bi2S3, the indirect transition defines pseudo-direct semiconductor. Authors mentioned other materials like dichacogenides, indium selenide

and phosphorene possessing similar characters, and propose that the effect is caused by layered nature, why not have a deeper research in here?

The Reviewer asks whether the pseudo-direct concept can be extended to other layered materials. We note that our work is a proof-of-principle study that establishes both the existence and the photophysical consequences of pseudo-direct bandgap character in a semiconductor, for the first time and on a rigorous experimental and theoretical basis. Crucially, our choice of Bi_2S_3 for this demonstration is deliberate rather than incidental: first, Bi_2S_3 is technologically relevant as a non-toxic solar absorber, with an ideal absorption onset ~1.4 eV for photovoltaic and photocatalytic applications (see for instance refs. 35-37 in the main text). Second, it has attracted increasing attention in the rapidly expanding field of perovskite-inspired materials (PIMs) owing to its similarity to other PIMs, such as $AgBiS_2$, BiSI, and $CuBiS_2$, among others. Third, its bandgap nature has remained controversial for many years (see refs. 40-43 in the main text). This made Bi_2S_3 an ideal platform for experimentally establishing the pseudo-direct concept.

We agree with the Reviewer that the broader applicability of this concept is an exciting and important direction for future work. In the revised manuscript, we have therefore now expanded the discussion of both future identification and direct engineering of pseudo-direct semiconductors.

As a first approach, we propose that high-throughput screening approaches based on large materials databases, such as the Materials Project (*Nat. Mater. 24, 1522–1532 (2025)*), could be used to identify candidate pseudo-direct semiconductors by using the direct–indirect energy splitting (ΔE) as a descriptor. Such exploration should not only discover other materials with a pseudo-direct character but also target materials with properties desirable for specific applications (e.g., bandgap for sunlight absorption, absorption coefficients, effective masses, transport properties, electronic dimensionality, defect properties, etc.). This approach would initially consist of screening the Materials Project (*Nat. Mater. 24, 1522–1532 (2025)*) (> 150k compounds) to identify semiconductors with a direct-indirect band gap difference of up to 2-3 times k_BT at room temperature. A secondary step would further refine the candidate pool based on the criteria of strong optical absorption in the visible range and good charge-carrier transport; this screening stage would rely on the use of hybrid functionals such as PBE0 (*J. Chem. Phys. 105, 9982–9985 (1996)*), HSE (*J. Chem. Phys. 118, 8207–8215 (2003)*), or more

recently developed range separated hybrid functionals (PNAS 2021, 118(34), e2104556118), which have documented superior accuracy for predictions of band gaps and band structures. The overarching goal will be to filter candidates that couple the pseudo-direct bandgap character with other desirable traits, such as high absorption coefficients, low effective masses, electronic dimensionality, and favourable defect properties (i.e., shallow defect character). In addition, we show that tuning van-der-Waals interactions may be able to directly influence the value of ΔE.

Therefore, our study establishes the concept of pseudo-direct character as a principal descriptor for this search, which can be readily implemented in broad screening studies.

To address the Reviewer's comment, we have now revised the Discussion section accordingly to better frame the generality and future development of the pseudo-direct semiconductor concept. The revised last paragraph in the Discussion section (p. 18-19) now reads:

"*Our modelling (Figure 3C and Figure S15) suggests that either identifying new pseudo-direct semiconductors with targeted ΔE values or implementing approaches to directly control ΔE will be crucial areas of development. We envision a strategy to discover possible pseudo-direct candidates that builds on a two-phase methodology: (i) an initial coarser screening of large material databases – e.g., the Materials' Project (72) – to identify candidate semiconductors and insulators showing* $\Delta E \sim 2 - 3\ k_B T$*, and (ii) a refinement of the candidate pool based on their band gap magnitude calculated using hybrid functionals, and further sift through pseudo-direct candidates for other desirable traits, such as high absorption coefficients, low and isotropic effective masses, and favourable defect chemistries. A second strategy is to control and tune ΔE values directly through external stimuli. For example, we propose that for materials with van der Waals nature of atomic bonding, such as in* Bi_2S_3*, and similarly transition metal dichalcogenides (73, 74), indium selenide (75, 76), and phosphorene (77), in which direct-indirect character crossovers have been reported, inter-ribbon(or inter-layer) interactions may be exploited to tune ΔE. For example, our calculations (Figure S18) reveal that when increasing the distance between* $(Bi_4S_6)_n$ *ribbons along the a- and c-axis of the* Bi_2S_3 *crystal structure (i.e., when expanding the lattice), ΔE evolves in the range between ~10 to ~100 meV. We note that in such van der Waals-bonded, layered materials, interlayer interactions may be tuned through exfoliation (78), strain (40), and intercalation (79)*

strategies, with the potential to directly control intrinsic bimolecular recombination processes in pseudo-direct semiconductors."

2. Lack of experimental support

In Discussion (Page 18), the authors state that "charge-carrier recombination lifetimes in such pseudo-direct semiconductors can potentially be extended by up to two orders of magnitude ...," citing simulation results in Figure S14. While this simulation is informative, is there any experimental evidence or existing examples to support this?, if generalizable, could offer a new materials design strategy for solar energy conversion.

We thank the reviewer for raising this important point. We would like to emphasise that experimental evidence for suppressed charge-carrier recombination had been provided as a central part of this work. Specifically, our fluence-dependent OPTP measurements on Bi_2S_3 thin films (see Figure 2B) directly revealed a bimolecular recombination rate of $k_2 \sim 5\times10^{-11}$ $cm^3\ s^{-1}$. This value is unusually low considering the simultaneously measured high absorption coefficient of Bi_2S_3, which is $5 - 10\times10^4\ cm^{-1}$ across a large portion of the visible spectrum. Given that bimolecular recombination is linked to absorption via the van Roosbroeck-Shockley relation (vRS), this discrepancy is direct indication that the concept of the pseudo-direct semiconductor applies to Bi_2S_3.

For comparison, the conventional direct semiconductor gallium arsenide (GaAs) exhibits significantly lower absorption coefficients near the band edge (typically in the range of $\sim 10^4$ cm^{-1}; *Phys. Rev. 127, 768, 1962*) but substantially larger bimolecular recombination coefficients (in the range 3–7 x $10^{-10}\ cm^3s^{-1}$; DOI 10.1016/j.solmat.2009.01.020, Nano Lett. 2017, 17, 9, 5782–5789) compared with Bi_2S_3. The coexistence in Bi_2S_3 of strong direct-like absorption, together with a suppressed bimolecular recombination coefficient, therefore constitutes clear experimental evidence that absorption and recombination are partially decoupled.

Our modelling in Figure S14 (Figure S15 in the revised manuscript) formalises this observation quantitatively and demonstrates that the measured suppression is consistent with a pseudo-direct gap splitting of approximately 40 meV, which is in good agreement with the value computed from first-principles.

We thank the reviewer for pointing out that this needed further emphasis and have now clarified this point further in the revised manuscript. The initial paragraph of the discussion now reads:

"The absorption coefficient of Bi_2S_3 is dominated by direct, strong interband transitions ($\alpha > 10^5$ cm^{-1}) arising within tens of meV of the band edges, making this a promising solar absorber. However, within tens of meV below the band edges associated with direct transitions lies an indirect transition, which slows down intrinsic bimolecular recombination processes in this material appreciably. *The combination of our temperature-dependent absorption and temperature- and fluence-dependent OPTP results, together with vanRoosbroeck-Shockley modelling (Figure 3C and Figure S15) demonstrates* that such a configuration suppresses the bimolecular recombination rates in Bi_2S_3 by ~ 75% at room temperature as calculated for $\Delta E = 40\ meV$."

Minor Comments

1. Language and Grammar:

Page 4, Lines 1-5: "However, the associated..." This sentence might be a bit long. Consider splitting it for improved readability?

We agree with the reviewer that this sentence is too long. We have now split it to two parts to improve the readability of the manuscript.

2. Tables in SI consecutiveness

Why is there only Table 2 in the Supplementary Information but no Table 1?

We thank the Reviewer for noticing this point, this typo has now been rectified. The tables have been re-numbered correctly.

3. Lack of experimental support

The authors report DFT-calculated indirect bandgap 1.0 eV and direct bandgap 1.04 eV. Although it might be indirectly proved in other figures, but a direct experimental verification would be more convincing.

We agree that a direct experimental determination of the direct–indirect energy splitting would be desirable. However, this is intrinsically challenging for pseudo-direct semiconductors. The key issue is that the indirect transition is both substantially weaker than the nearby direct

transition owing to its phonon-assisted character, and (by pseudo-direct semiconductor design) must be separated from the direct transition by only a few tens of meV.

For any indirect semiconductor, the low absorption coefficient (~10^0-10^2 cm^{-1}) of indirect transitions makes experimental measures of their bandgap already a challenge on its own. For conventional indirect bandgap semiconductors such as silicon and germanium, where the difference between the fundamental indirect transition and the higher-lying direct transition is very large, the onset of the indirect transition has been quantified. However, for pseudo-direct bandgap semiconductors $\Delta E \sim k_B T$ at room temperature, such a determination is made significantly more challenging by the close proximity of the direct transition. In this case, any broadening factors of the direct transition – such as phonons, disorder, optical interference effects – will conceal the indirect onset in optical measurements. For instance, both phonon broadening and the Urbach tail are expected to be in the range of a several tens of meV (see *J. Phys. Chem. Lett. 2022, 13, 33, 7702–7711*), thus making it very hard to disentangle the indirect onset from these contributions. Given the orders-of-magnitude difference between the direct and indirect absorption coefficients, optical absorption measurements thus predominantly probe the direct transition.

For this reason, our determination of the pseudo-direct character relies on the combined consistency between temperature-dependent recombination measurements and van Roosbroeck–Shockley modelling as well as DFT calculations. Importantly, all these approaches independently converge toward a direct–indirect splitting of approximately 40 meV.

Conclusion

This is an excellent piece of work that introduces a significant new concept to the field. The experimental data is robust, and the theoretical modelling supports the conclusions well. The only poential gap is that all the conclusions come from Bi2S3. As a result, due to it's universality, I recommend rejection. Given the strength of the experimental data on Bi2S3 and the relevance to nanostructured materials, the work would be well suited to the scope of Nature Materials, which regularly publishes studies on the fundamental properties of emerging nanomaterials.

We thank the Reviewer once again for acknowledging the value of our work and for the helpful suggestions for improving the manuscript.

Reviewer #2

In this study, Righetto et al. propose a compelling "pseudo-direct" semiconductor model to decouple light absorption from charge-carrier recombination, aiming to circumvent the fundamental constraints of the van Roosbroeck-Shockley (vRS) relation. This work is highly relevant and timely, as the optical reciprocity principle, where efficient absorbers are inherently efficient emitters, often imposes a ceiling on the open-circuit voltage (Voc) and limits carrier lifetimes in photovoltaic and photoelectrochemical devices. The authors focus on the unique electronic structure of Bi2S3, where the presence of multiple indirect transition states at energy levels nearly degenerate with the direct gap offers a potential pathway to suppress intrinsic recombination without compromising absorption. The authors categorize Bi2S3 as a direct semiconductor given its high absorption coefficient (alpha > 10^5 cm^-1), yet they acknowledge an absorption profile that mimics indirect semiconductors. Through JDOS analysis and low-temperature absorption measurements, they argue that the observed optical behavior is dominated by direct transitions, despite a calculated indirect gap that is approximately 40 meV lower. The THz conductivity measurements provide experimental evidence for this decoupling; Bi2S3 exhibits a significantly suppressed bimolecular recombination coefficient (k2), suggesting that carriers relax into a transient "reservoir" of indirect states. The concept is timely and potentially significant. However, in its current form the manuscript contains several experimental and theoretical gaps that must be addressed before it meets the high standard required for Nature.

We thank the reviewer for the constructive scientific feedback on our investigation and for acknowledging the significance of our work. We have addressed the points as follows below:

1. The central mechanistic claim — that photoexcited carriers scatter from the direct Γ valley into the indirect reservoir states — is supported only by macroscopic observables. While the suppressed k2 and the THz conductivity data are consistent with this picture, they do not constitute momentum-resolved proof of the proposed population transfer. Time-resolved ARPES (TR-ARPES) would provide direct, valley-selective tracking of carrier dynamics and would substantially strengthen the mechanistic interpretation. Without such data, the intervalley scattering pathway remains an assumption, and alternative explanations for the suppressed k_2 cannot be excluded.

The Reviewer suggests that time-resolved ARPES experiments should be carried out to directly observe intervalley scattering for Bi_2S_3. tr-ARPES uses high-energy photons with a few-femtosecond resolution to investigate the ultrafast dynamics across momentum space However, while such experiments are fascinating in general, we note that they would not be able to discern the presence of a pseudo-direct semiconductor. This is because in tr-ARPES, achieving the temporal resolution required to capture sub-100-fs intervalley scattering necessarily broadens the energy resolution because of the Fourier/Heisenberg uncertainty relation. Therefore, any measurement capable of resolving the intentionally small energy spacing $\Delta E \sim 40\ meV$ between the bands involved in the direct and indirect transitions would not have the time resolution to disentangle the intervalley scattering process. For example, the trade-off between short time resolution and broad energetic resolution has limited the energetic resolution of tr-ARPES to 150-60 meV [*Phys. Rev. Lett. 115, 086803 (2015)*] in more conventional tr-ARPES measurements. Meanwhile, lower energetic resolutions (30 meV, see *Nat Commun 10, 3535 (2019)*) were achieved only with a ~200 fs time-resolution conditions, unable to capture fast intervalley scattering processes (usually reported at ~10-50 fs, see *Appl. Phys. Lett. 53, 2089–2090 (1988)* for GaAs, *Appl. Phys. Lett. 109, 162102 (2016)* for MoS_2). Crucially, the trade-off is a fundamental, originating from the Heisenberg uncertainty principle, which causes the Fourier-limited bandwidth of a ~15 fs pulse to be >100 meV. Therefore, we note that observing these processes in Bi_2S_3 thin films (as well as in other pseudo-direct semiconductors) in which the ΔE is purposely on the order of $k_B T$ at room temperature cannot be addressed using TR-ARPES.

Importantly, we would like to stress that the most direct proof for pseudo-direct semiconductor behaviour is the combined study we have carried out here, i.e. temperature-dependent k_2 data, absorption data, and first-principles modelling. The combination of these data strongly supports the pseudo-direct bandgap character of Bi_2S_3 and cannot be reconciled with a purely disorder- or trap-mediated origin.

We have now added a paragraph explicitly discussing alternative scenarios in charge-carrier dynamics, such as disorder or charge-carrier trapping (further discussed in Point 6 below), and why they cannot reproduce the observed trend. The Supplementary Note 1 now reads:

"As a result, our fluence-dependent OPTP measurements enable effective decoupling of monomolecular and bimolecular recombination processes in Bi_2S_3. Such decoupling arises from the distinct dependencies on the charge-carrier density n (see Equations S1), which is varied via the excitation fluence parameter (see Equation S3). Here, owing to the different n-scaling of the mono- and bimolecular terms, increasing excitation fluence enhances recombination through the bimolecular channel relative to the monomolecular channel. Such behaviour is observed as an accelerating photoconductivity decay at early times, which leaves a photoconductivity tail dominated by monomolecular processes. Such a process underlies the characteristic "fanning-out" of normalised transients with increasing fluence that is clearly observed in our Bi_2S_3 datasets (Figure 2D) and represents a hallmark of bimolecular electron–hole recombination.

Crucially, such fluence-dependent behaviour cannot be reproduced by disorder-related effects nor trap-mediated processes, even in the case of a finite trap density. On the one hand, the effect of disorder is fully captured in our model by the KWW formulation of the k_1 parameter, as disorder mainly broadens the distribution of monomolecular rates but does not change the order of the rate law. On the other hand, in the presence of finite trap densities, photogenerated charge carriers can fill the available trap states in a process known as trap saturation(25-27). The result of this process is an effectively suppressed monomolecular recombination rate. It is worth noting that trap-saturation effects yield slower decays at higher fluence, which is the opposite behaviour to what is observed in our Bi_2S_3 OPTP transients. Therefore, the observed acceleration of photoconductivity decay at increasing excitation fluences unambiguously identifies the intrinsic second-order term as intrinsic bimolecular electron-hole recombination."

We have further clarified this in Main Text on page 9, as follows:

"We modelled the charge-carrier dynamics through a k_1k_2 model, which is capable of clearly separating contributions from monomolecular (e.g., defect-mediated, extrinsic) and bimolecular intrinsic (electron-hole band-to-band) recombination processes (see detailed description in Supplementary Note 1)."

2. The manuscript references Figure S16 in connection with inter-ribbon interactions and their influence on the electronic structure, but this figure does not appear in the current version of

the Supporting Information. This data should be provided, as the role of structural coupling between ribbons is directly relevant to the computed band structure and, by extension, to the ΔE value on which the entire pseudo-direct framework rests.

We thank the reviewer for spotting this mistake; the references to Figure S16 on page 4 of the Supporting Information should have been to the original Figure S15 instead, now Figure S18. This figure already reports the dependence of the electronic band gaps and the direct–indirect splitting on the inter-ribbon spacing. Therefore, we hope that with the corrected figure reference, this satisfies the reviewer's comment. We have also carefully re-checked that all figure references in the main manuscript and SI are now correct following the addition of further figures.

3. Several aspects of the DFT calculations require clarification. Regarding the computational methodology, the authors should specify the energy and force convergence criteria for structural optimization. Furthermore, please clarify how the Pulay stress was managed during the variable-cell relaxation (vc-relax) process, as it can significantly affect the lattice parameters in such systems.

We thank the referee for this point, which allows us to further clarify our approach. In fact, we did *not* perform a variable-cell structural optimisation for the structural models used in our calculations.

Instead, all electronic-structure calculations were performed using experimentally refined crystal structures reported in the literature (reference 7 of the SI), which is why we do not include a discussion on Pulay stress or energy and force convergence criteria. Incidentally, we have separately checked that structural optimization does not have a significant effect on the energetic spacings between the direct and indirect gaps. We have now clarified this point in the revised Supporting Information, and thank the reviewer again for pointing out this gap in our presentation.

The description of the computational methods has now been updated as follows:

"Ab initio calculations based on Density Functional theory (DFT) were carried out for this work, as implemented in the plane wave basis code Quantum Espresso (2, 3) using the Perdew-

Burke-Ernzerhof exchange correlation functional (PBE)(4), including spin-orbit coupling (DFT/PBE+SOC). We used the standard norm-conserving fully relativistic Vanderbilt pseudopotentials (5) from the PseudoDojo database (6). Calculations were performed with a kinetic-energy cut-off of 75 Ry, with a uniform, Γ-centered k-point grid (7) of (4x6x4). We used the experimental Bi_2S_3 structure, as reported in (8) in all calculations reported in this study, with the exception of calculations shown in Figure S18, for which details are given below.

We computed the effective masses at the band extrema using the finite difference method, as implemented in the Effective Mass Calculator utility (9). We used a 5-point stencil with a k-step size of 0.019 $Å^{-1}$ to compute the second derivative of the energy with respect to the wave vector. We performed hybrid functional calculations using the global PBE0 functional (10), including spin-orbit coupling, in order to check the robustness of the band edge shape computed from PBE+SOC."

4. Importantly, the omission of van der Waals (vdW) corrections is a significant concern. For a quasi-1D material like Bi2S3, where inter-ribbon distances are governed by dispersion forces, the lack of a vdW scheme (e.g., DFT-D3) could lead to inaccurate lattice parameters. Given the extreme sensitivity of the Delta E (~40 meV) offset to the structural environment, it is imperative to verify if these energy levels remain consistent when vdW interactions are properly accounted for.

We thank the Reviewer for raising this important point. As we have already pointed out in the previous point, all electronic structure calculations reported in the manuscript are performed using the experimental structure of Bi_2S_3. This is a deliberate choice, so as to ensure that our calculations do not incur additional systematic errors originating from the computational setup of structural optimization calculations (e.g. different exchange correlation functionals, consideration of dispersive forces). Prior studies (Phys. Rev. B (2013) 87, 205125), as well as our tests confirm that while structural optimization may change slightly the precise difference between the direct and indirect band gap, the pseudo-direct nature of the band gap remains consistent regardless of whether structures are experimentally determined or computationally optimized.

We have used fixed cell structural optimization only for the study of band gap trends with increasing inter-ribbon distance (Figure S18 of the SI). As described in the SI, we have modelled this by initially increasing the lattice parameters a and c, and translating the ribbons along the a and c directions rigidly without distorting them, and then by allowing the atomic positions to relax in the expanded structure. Here, we believe the referee's point is valid and that the impact of van der Waals interactions for these particular calculations should be checked.

To address this point, in the revised Figure S18, we now show a direct comparison of results computed with and without inclusion of van der Waals forces in the structural optimization calculations. We find only minor changes in the calculated direct and indirect band gaps, and very similar trends as seen below. These results are now included in the SI of the resubmitted manuscript.

We would also like to note that while doing this check, we have improved the precision of our calculations, and changed all panels in Figure S18 accordingly.

We have added the following figure to the Supplementary Information:

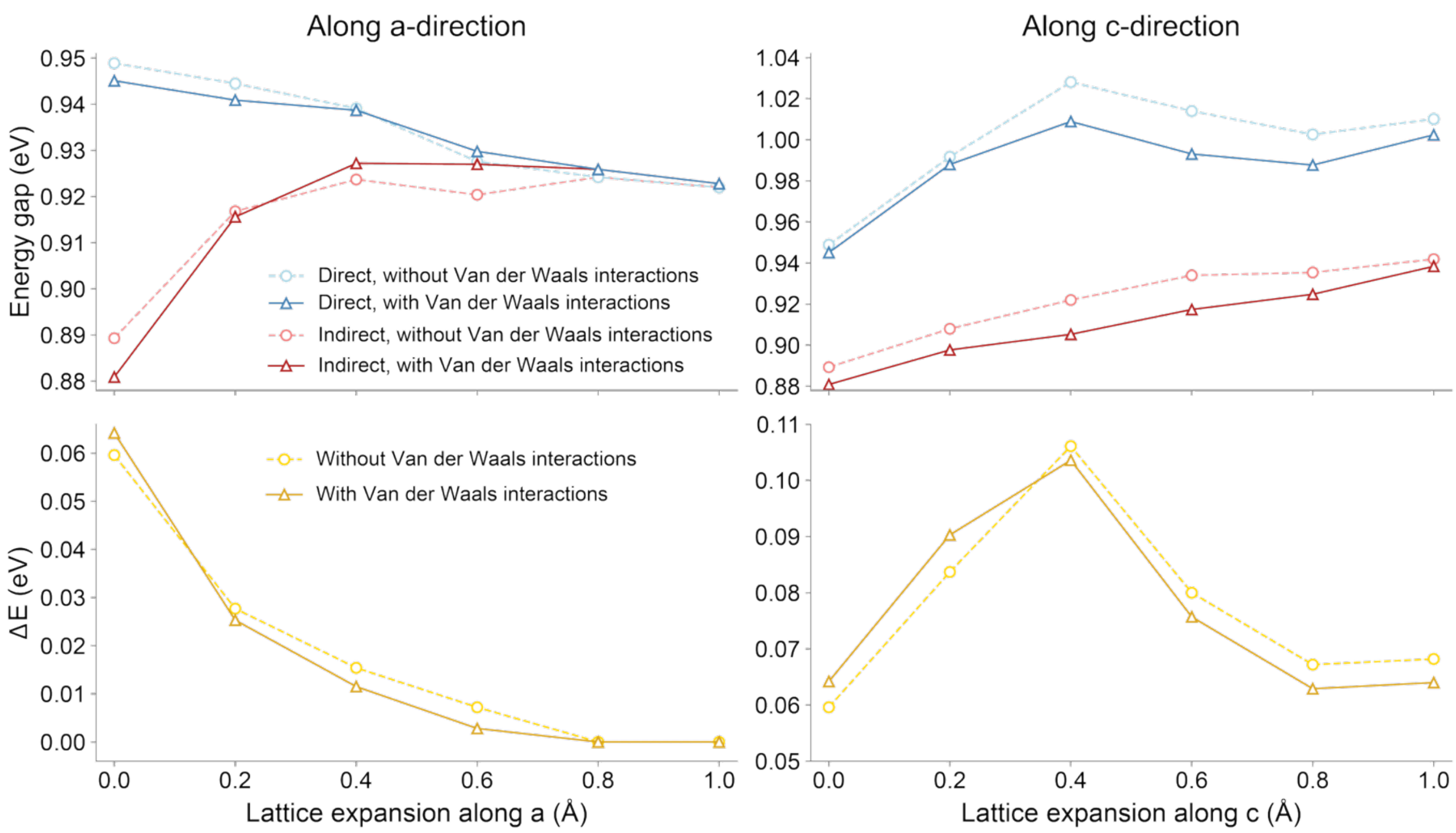

Fig. R2.1 | **Change in band gap with lattice expansion** Direct (lines with open blue symbols) and indirect (lines with open red symbols) band gaps calculated for structures with increasing inter-ribbon distance along the a and c directions. Lower panels show the corresponding energy difference between these transitions (i.e., the ΔE value). Circles and dashed lines indicate structures relaxed without van der Waals corrections, whereas triangles and solid lines denote structures relaxed including van der Waals interactions (see *J. Chem. Phys. 132, 154104 (2010)*), starting from structures with extended and fixed a and c lattice parameters, and shifted ribbons (ensuring the ribbons are undistorted). The direct and indirect band gaps are identified from calculations of energy eigenvalues over a Γ-centered k-point grid of 9x24x9.

5. All measurements are performed on thermally evaporated polycrystalline films. This is a significant confound, because grain boundaries and structural disorder are independently known to produce weak temperature dependence of carrier mobility, elevated monomolecular recombination rates, and broadened absorption onsets — precisely the signatures that the authors invoke as evidence for the pseudo-direct mechanism. As it stands, it is not possible to unambiguously distinguish intrinsic band-structure effects from extrinsic disorder-related phenomena. Reproducing the key observations on structurally well-defined material — for example, epitaxial films grown on lattice-matched substrates — would be an important step towards establishing that the reported behaviour is intrinsic.

We thank the Reviewer for raising an important point about our Bi_2S_3 thin-film samples and for giving us the opportunity to further clarify our argument. We reassure the Reviewer that we specifically chose optical-pump-THz-probe spectroscopy because this high-frequency AC technique monitors intragrain mobility, which is far less dependent on the microstructure and therefore generally reflects intrinsic mobilities probed over nanometer length scales.

The length scales probed by such linear THz spectroscopy are well documented in literature examining charge-carrier displacement driven by electric fields associated with the THz radiation. Under typical conditions, the THz field-driven displacement of charge carriers is intrinsically small within the period of the THz radiation (~1 ps). As discussed in detail by Lloyd-Hughes et al. (*J. Infrared Millim. Terahertz Waves 33, 871 (2012)*), for the low-field regime adopted in our measurements ($E_{THz} < 10^5\ V\ m^{-1}$), the field-induced charge-carrier excursion is below ~2 nm even for high-mobility semiconductors such as GaAs. Therefore, if

the excursion length scale is smaller than the grain size of the Bi_2S_3 thin films, such THz conductivity measurements probe intrinsic transport largely unaffected by grain boundaries.

To directly establish this comparison, we have now included in the revised manuscript information on crystalline grain size derived from scanning electron microscopy (SEM) on our Bi_2S_3 thin films. The SEM micrograph (Figure R2.2, shown below) reveals grain sizes on the order of ~100 nm, which is about two orders of magnitude larger than the THz-drive charge-carrier excursion. Therefore, this experiment probes the intrinsic intra-grain mobility of charge carriers.

As a second important piece of evidence, we note that THz photoconductivity spectra for our Bi_2S_3 thin films exhibit a Drude-like response, i.e. a near-zero imaginary part and near constant real part (see new Figure R2.3 below). Such behaviour reflects the classic presence of a free charge carrier density with low momentum scattering time. There is no evidence in these spectra of localization or significant backscattering at grain boundaries on the length scale probed (see *J. Infrared Millim. Terahertz Waves 33, 871 (2012), Phys. Rev. B 96, 205439 (2017)*).

Both additional measurements therefore clearly support the assignment of our extracted mobility to the intragrain transport regime that reflects the intrinsic mobility of Bi_2S_3. Moreover, as already stated in the manuscript, this is further confirmed by the large THz-mobility values we obtained here ~15 $cm^2V^{-1}s^{-1}$, which significantly exceed the long-range DC mobility extracted on analogous films in a transistor configuration ~0.01-0.001 $cm^2V^{-1}s^{-1}$ (Appl. Phys. Lett. 122, 192102 (2023)).

We therefore reassure the Reviewer that both our technique and our analysis directly probe the intrinsic photophysical processes in Bi_2S_3. We thank the reviewer for highlighting that this required clarification and have now added the additional SEM and THz spectral data to the revised Supplementary Note 3, together with further explanations and discussions on this point.

The additional section of this note reads:

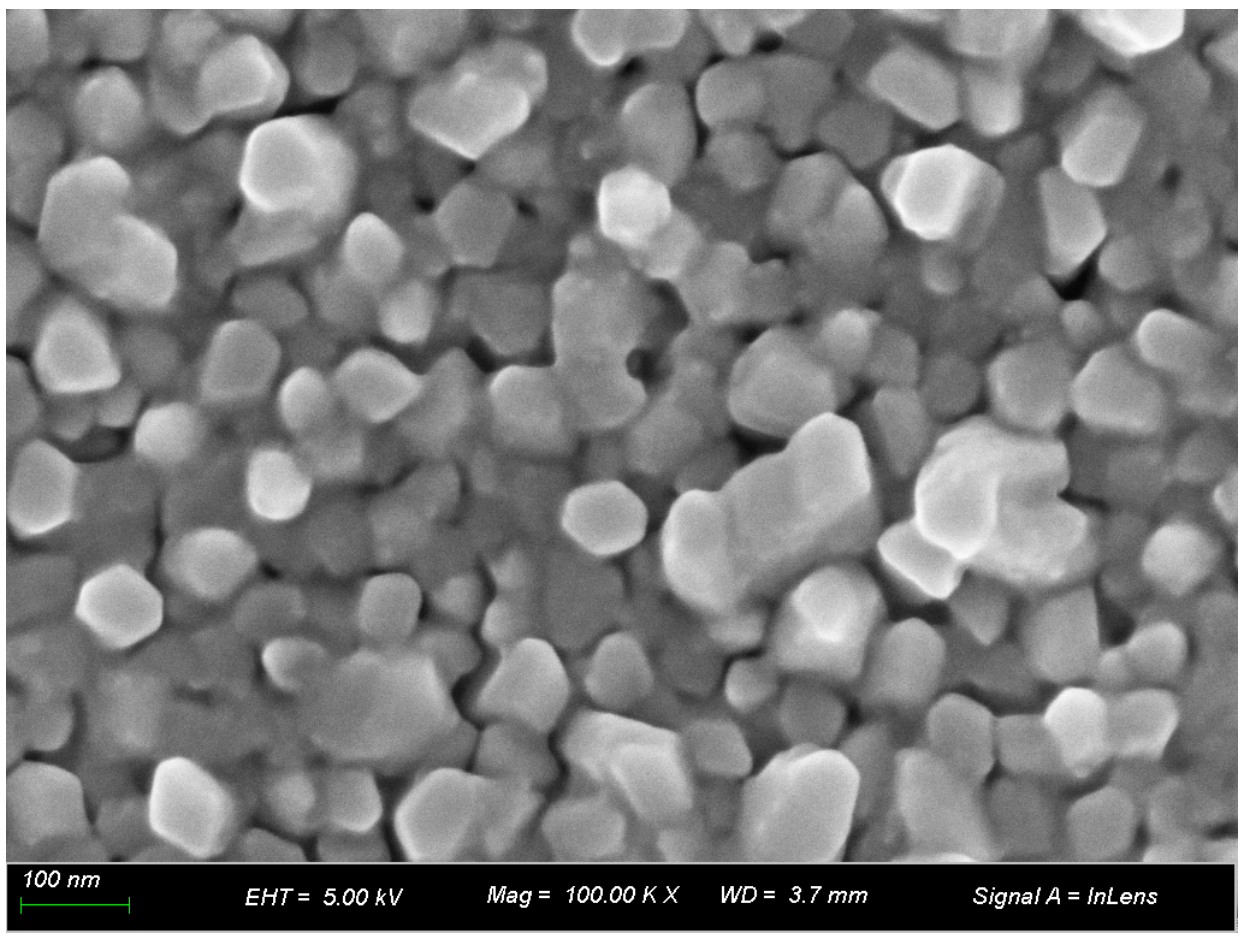

Figure R2.2 | **Surface morphology of Bi_2S_3 thin films**. The surface morphology of the films was characterised using a Zeiss GeminiSEM 500 field-emission scanning electron microscope (SEM). All measurements were performed under high-vacuum conditions at an acceleration voltage of 5 kV and a working distance of 3.7 mm in secondary electron (SE) mode. The samples possess sufficient electrical conductivity to prevent charging artifacts during imaging, and therefore, no metallic coating was applied. SEM micrographs show grain sizes on the order of ~70-100 nm.

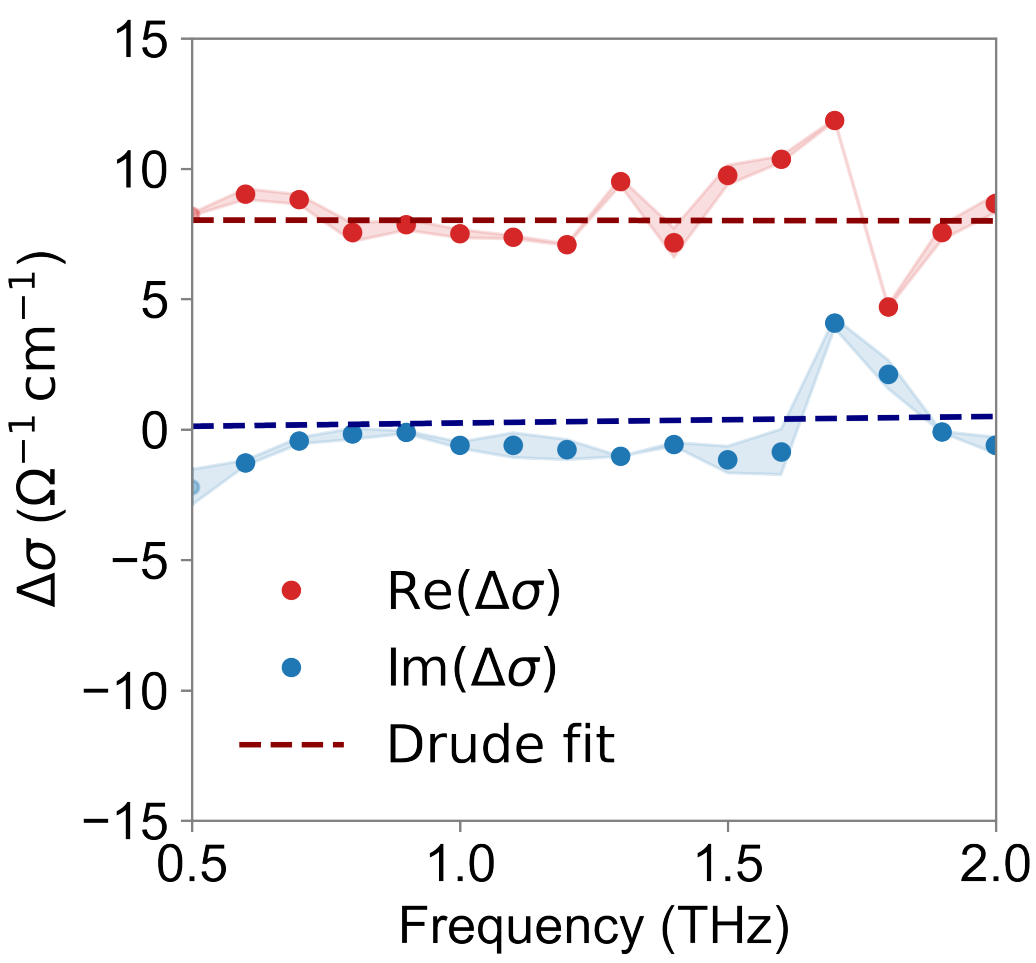

Figure R2.3 | Photoconductivity spectrum for Bi_2S_3 thin film. Real (red) and imaginary (blue) part of the photoinduced THz conductivity spectra for Bi_2S_3 thin films, measured at 4 K around the peak photoconductivity signal (t = 2 ps) following 1.55 eV excitation at a fluence of 15 μJ cm^{-2}. Dots represent experimental data (with shaded areas indicating statistical error), whereas dashed lines are fits according to the Drude model. We note that the near-zero imaginary part and near-constant real part reflect the classic Drude picture for a free density of charge carriers

with low momentum scattering time, ruling out significant backscattering contributions from grain boundaries.

"***Absence of grain-boundary effects on extracted THz charge-carrier mobilities***

The photoconductivity signal obtained by the OPTP technique, which underlies the extracted charge-carrier mobility values, reflects intrinsic intragrain transport and recombination dynamics. As a result, the THz-derived charge-carrier mobilities for Bi_2S_3 thin films ($\sim 15\ cm^2V^{-1}s^{-1}$) *are significantly higher than the long-range DC mobilities extracted from Bi_2S_3-based devices (* $\sim 0.01 - 0.001\ cm^2V^{-1}s^{-1}$ *for Bi_2S_3-based transistors) (48).*

Such selectivity of THz spectroscopy in polycrystalline thin films towards intragrain transport originates from the length scales probed in linear THz spectroscopy. In OPTP measurements, the photogenerated charge carriers are displaced by the oscillating electric field associated with the THz radiation. As discussed in detail by Lloyd-Hughes et al. (49), for the low THz-fields analogous to those used in our measurements ($E_{THz} < 10^5\ V\ m^{-1}$)*, the field-induced charge-carrier excursion is below ~2 nm even for high-mobility semiconductors such as GaAs. Crucially, this excursion length scale is significantly smaller than the size of the Bi_2S_3 grains in our thin films, as shown in Figure S16, thereby implying that THz conductivity measurements probe intrinsic transport largely unaffected by grain boundaries.*

Our hypothesis is further supported by the THz photoconductivity spectra measured for our Bi_2S_3 thin films. Importantly, several works have reported how nanoscale morphologies (for instance, in nanocrystals and quantum dots (47, 50)) can yield backscattering processes that determine a specific phase relation between the oscillating electrons and the THz field, summarised in the Drude-Smith model (51). The effect of surfaces is therefore observed mainly in THz photoconductivity spectra as the emergence of a non-zero imaginary part and a non-monotonous real part of the photoconductivity. Conversely, the THz photoconductivity spectra recorded at 4 K on the photoconductivity peak (Figure S17) exhibit a Drude-like response, i.e. a near-zero imaginary part and a near-constant real part. Such behaviour reflects the classic presence of a free charge-carrier density with low momentum scattering time. Here, the Drude-like behaviour of charge carriers in our Bi_2S_3 thin films confirms the absence of grain-boundary-induced effects."

We have now also clearly pointed to this Supplementary Note 4 in the Main Text on page 13:

"We note that the THz AC conductivity probes deployed here mostly reflect intra-grain transport (see Supplementary Note 4)."

6. Closely related to the above is the question of defect chemistry. Sulfur vacancies are thermodynamically favourable under typical chalcogenide growth conditions and are expected to introduce states within the bandgap. The stretched-exponential trapping behaviour and the large monomolecular recombination component k1 observed in the present films are both consistent with a significant density of such defect states. Under these circumstances, the suppression of k2 may reflect, at least in part, carrier trapping and localisation rather than solely the intrinsic pseudo-direct band structure. The manuscript would benefit from a more explicit characterisation or discussion of defect densities and their electronic impact — for instance, through sub-bandgap spectroscopy or temperature-dependent admittance measurements — to delineate the relative contributions of intrinsic and extrinsic processes.

We agree that defects contribute to the monomolecular recombination channel (k_1) in Bi_2S_3 thin films, as already discussed in the manuscript (page 8). However, our conclusions regarding pseudo-direct recombination are based specifically on the temperature-dependence of the bimolecular rate constant (k_2).

As described in Supplementary Note 1, fluence-dependent measurements allow the clear separation of the k_1 from k_2 because trap-mediated recombination involves just one species (a trapped electron or hole) while bimolecular electron-hole band-to-band recombination involves two particles and thus becomes faster at higher charge-carrier density. We further note that while trap saturation effects, in which traps are completely filled by photogenerated charge carriers, may sometimes introduce a fluence-dependence of trap-mediated recombination, this would yield longer lifetimes at higher fluence, and the opposite trend is observed in our measurements. Our global fits to sets of multiple photoconductivity transients recorded for a range of fluences therefore provide a reliable way of separating trap-mediated (k_1) processes from intrinsic electron-hole recombination (k_2) governed by the bandstructure. We note that such separation of processes is an established and well-reported practice in the analysis of such transients (see e.g., Nat Commun 6, 7903 (2015), J. Phys. Chem. C 2017, 121, 37, 20451–20457, Nat Mater 16, 115–120 (2017)).

Furthermore, our assignment of the observed fluence-dependent behaviour to radiative bimolecular electron-hole recombination is substantiated by PL measurements, which show the emergence of stronger PL signal at fluences for which bimolecular recombination becomes more dominant.

We have now expanded the discussion in Supplementary Note 1 to clarify why defect-mediated trapping cannot account for the observed behaviour. The Supplementary Note 1 now reads:

"As a result, our fluence-dependent OPTP measurements enable effective decoupling of monomolecular and bimolecular recombination processes in Bi_2S_3. Such decoupling arises from the distinct dependencies on the charge-carrier density n (see Equations S1), which is varied via the excitation fluence parameter (see Equation S3). Here, owing to the different n-scaling of the mono- and bimolecular terms, increasing excitation fluence enhances recombination through the bimolecular channel relative to the monomolecular channel. Such behaviour is observed as an accelerating photoconductivity decay at early times, which leaves a photoconductivity tail dominated by monomolecular processes. Such a process underlies the characteristic "fanning-out" of normalised transients with increasing fluence that is clearly observed in our Bi_2S_3 datasets (Figure 2D) and represents a hallmark of bimolecular electron–hole recombination.

Crucially, such fluence-dependent behaviour cannot be reproduced by disorder-related effects nor trap-mediated processes, even in the case of a finite trap density. On the one hand, the effect of disorder is fully captured in our model by the KWW formulation of the k_1 parameter, as disorder mainly broadens the distribution of monomolecular rates but does not change the order of the rate law. On the other hand, in the presence of finite trap densities, the photogenerated charge carriers can fill the available trap states in a process known as trap saturation(25-27). The result of this process is an effectively suppressed monomolecular recombination rate. It is worth noting that trap-saturation effects yield slower decays at higher fluence, which is the opposite behaviour to what is observed in our Bi_2S_3 OPTP transients. Therefore, the observed acceleration of photoconductivity decay at increasing excitation fluences unambiguously identifies the intrinsic second-order term as intrinsic bimolecular electron-hole recombination."

We have further clarified this in the Main Text on page 9, as follows:

"We modelled the charge-carrier dynamics through a k_1k_2 model, which is capable of clearly separating contributions from monomolecular (e.g., defect-mediated, extrinsic) and bimolecular intrinsic (electron-hole band-to-band) recombination processes (see detailed description in Supplementary Note 1)."

7. While the manuscript highlights potential implications for solar energy conversion, no device-level validation is provided. Demonstrating the feasibility of this concept in an actual solar cell device, or at least providing a quantitative estimate of the expected gain in quasi-Fermi level splitting or open-circuit voltage, would greatly strengthen the relevance of the work.

The Reviewer requests clarification on the impact of this new concept of a pseudo-direct semiconductor on solar cell devices. We thank the Reviewer for raising this constructive point, which we address below. The key idea conveyed in our work is that the pseudo-bandgap character of the electronic band structure can suppress the intrinsic bimolecular recombination channel. Suppressing this recombination channel has multiple substantial impacts on a solar-energy conversion device:

(i) Suppressing this channel reduces fundamental losses in a solar cell. These losses (which include recombination losses, and only partially the Boltzmann losses) amount to several per cent, depending on the bandgap of the absorber: for example, precise calculations have been made based on the original Shockley-Queisser concept by Hirst et al. (DOI: 10.1002/pip.1024). Suppressing bimolecular electron-hole recombination will allow us to circumvent these fundamental limits for pseudo-direct semiconductor-based devices operating at the radiative limit.

(ii) Reducing k_2 implies an improved mobility-lifetime $\mu\tau$ product, as for optimised solar absorbers operating at or near the radiative limit, the value of τ equals the inverse nk_2 product, where n is the charge carrier density at solar irradiation densities. As a result, the charge-carrier diffusion length is expected to increase, thereby reducing transport losses and improving short-circuit current (Jsc) parameters in devices operating below the radiative limit.

(iii) Beyond solar cell applications, the suppression of the nk_2 term implies longer charge-carrier lifetimes. Such a condition is essential for applications in

photocatalysis, where longer lifetimes are necessary to enable time for surface chemical reactions to occur. Importantly, such a scheme trading off photovoltage for longer charge-carrier lifetimes is already adopted in nature: biological photosystems sacrifice part of their photovoltage to achieve gains in excited state lifetimes, as explained in Nat Rev Mater 7, 503–521 (2022).

Furthermore, as noted by the Reviewer in their comment #6, trapping still plays a significant role in the charge-carrier dynamics of these Bi_2S_3 thin films, thereby suggesting that solar cell device performance would be determined by the monomolecular term. Several photoelectric devices based on these Bi_2S_3 thin films have already been reported [see Appl. Phys. Lett 122 (19), 2023; Phys. Rev. Applied 19 (6), 064080, 2023; ACS Mater. Lett. 7 (9), 3159-3166, 2025; ACS Photonics 12(1), 457-463, 2024], and demonstrate the potential for these applications. However, importantly, device performance parameters on their own are unlikely to comprise a proof of pseudo-direct semiconductor operation, given the intrinsic nature of the effect. Such proof might be inferred indirectly if performance in excess of the Shockley-Queisser limit is recorded. However, this would require complete suppression of trap-mediated processes at solar illumination levels. Crucially, for silicon-based solar absorbers such optimization has taken 7 decades to achieve since the first demonstration of a silicon solar cell in 1954, with operation close to the radiative limit being approached only now (see NREL/NLR Solar Cell Efficiency Chart https://www.nlr.gov/pv/cell-efficiency).

We stress that unless the concept of pseudo-direct behaviour is highlighted now and promising pseudo-direct semiconductor candidates are found, such work to optimise these semiconductors will most probably never happen in the first place. Here lies the value and impact of our work on solar-energy conversion, as a trigger to discover such candidates and initiate optimization and implementation.

We have now added a Supplementary Note 5 that summarises these points, as follows:

"Supplementary Note 5: Impact of pseudo-direct bandgap character on solar energy conversion devices

The central idea of our work is that the pseudo-direct bandgap character, demonstrated here in Bi_2S_3 thin films, can effectively suppress intrinsic bimolecular recombination (up to 75% in Bi_2S_3) while still retaining a remarkably high absorption coefficient. Both these parameters

are key factors in determining the efficiency of solar cells and other solar energy conversion devices (e.g., photoelectrochemical cells). Therefore, we discuss the implications of a pseudo-direct character on these devices, based on three main points:

(i) *The charge-carrier lifetime in a semiconductor is generally limited by both trap-mediated monomolecular and intrinsic bimolecular recombination processes (25). As described in Supplementary Note 1, monomolecular recombination processes generally dominate in the low charge-carrier density regime. However, in optimised solar absorbers (e.g., silicon, GaAs, lead halide perovskites), the charge-carrier lifetime is mainly limited by bimolecular recombination processes* $\tau_{bim} = (nk_2)^{-1}$*, as a result of the reduced trap densities (and hence reduced* k_1 *rates)(41). As discussed in Supplementary Note 3, a pseudo-direct semiconductor such as* Bi_2S_3 *can achieve a ~75% suppression of the bimolecular recombination rate, with respect to a direct semiconductor with the same absorption coefficient* α*. As shown in Figure S19, the increase in lifetime translates into a longer charge-carrier diffusion length* $L_D=\sqrt{D\tau}$ *for optimised solar absorbers, which are expected to reduce transport and collection losses (i.e., potentially increasing short-circuit current and fill factor parameters) in devices. We note that, based on the extracted* k_1 *values for our* Bi_2S_3 *thin films, the charge-carrier lifetime at solar (AM 1.5) injection levels is dominated by the monomolecular term (see Figure S19). Therefore, this intrinsic advantage is currently latent and would emerge upon defect passivation.*

(ii) *The suppression of the intrinsic recombination channel also has the potential to reduce fundamental losses in a solar cell operating at the radiative limit. These emission-related losses (which include recombination losses and only partially Boltzmann losses) amount to several per cent, depending on the absorber's bandgap: for example, precise calculations based on the original Shockley-Queisser concept have been made by Hirst et al. (53). Beyond improving the efficiency of solar cells in an optimised regime (point (i)), the suppression of* k_2 *also has the potential to circumvent these fundamental limits for pseudo-direct semiconductor-based devices operating at the radiative limit.*

(iii) *The extended charge-carrier lifetime (discussed above) carries significant implications for applications beyond photovoltaics. Importantly, long charge-carrier lifetimes are crucial for applications in photocatalysis, where longer*

lifetimes are necessary to enable time for surface chemical reactions to occur. Importantly, such a scheme trading off photovoltage for longer charge-carrier lifetimes is already adopted in nature: biological photosystems sacrifice part of their photovoltage to achieve gains in excited state lifetimes, as explained in Ref.(54) ."

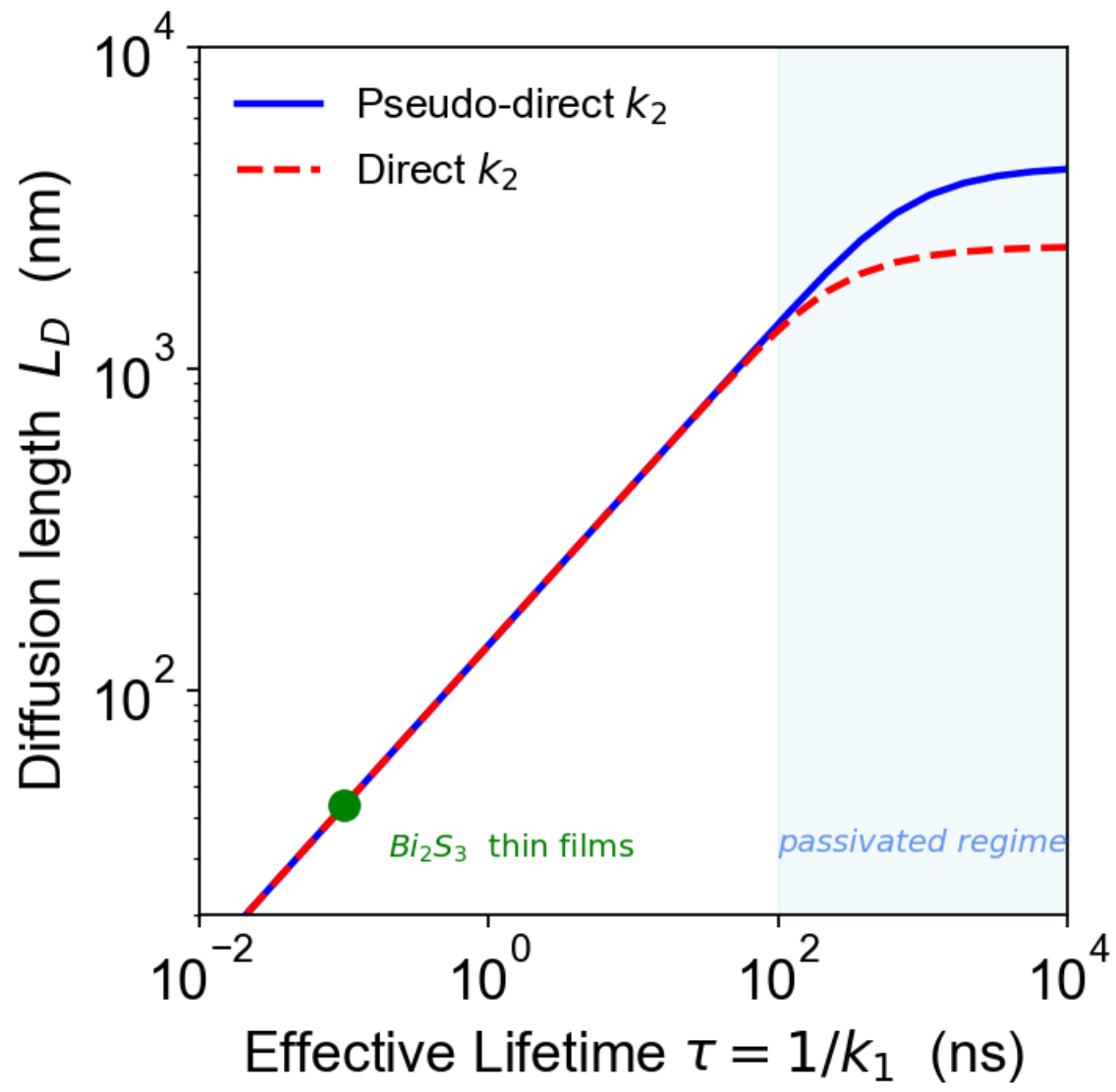

Figure R2.4 | Diffusion length calculations for Bi_2S_3 thin films. Calculated charge-carrier diffusion length as a function of the effective charge-carrier lifetime for a pseudo-direct bandgap character (blue solid line) and for a standard direct bandgap character (red dashed line). The green dot indicates the obtained values for k_1 and k_2 experimentally obtained for Bi_2S_3 films examined in this study. The k_2 value for the pseudo-direct line is value of $k_{2,Bi2S3} = 5 \times 10^{-11}$ cm^3 s^{-1} experimentally determined at 300 K, while for the direct line, we use the extracted 75% suppression, i.e. $k_{2,direct} = 4 \times k_{2,pseudodirect}$. The comparison highlights that the impact of a pseudo-direct bandgap character will only become apparent in the radiative regime (blue shading) for a well-passivated material.

Minor Comments

1. Terminology: For academic precision, "conduction band bottom" and "valence band top" should be replaced with Conduction Band Minimum (CBM) and Valence Band Maximum (VBM).

We thank the reviewer for raising this point. We have now changed this terminology to the more conventional CBM and VBM both in the Main text and in the Supplementary Information.

2. Clarification of Extremas: On page 14, the discussion of "valence band minima and maxima" should be clarified to specify whether the authors are referring to higher-lying or lower-lying sub-bands.

We thank the reviewer for pointing this out; we have corrected a typographical error in the discussion, which now reads more clearly.

Reviewer #3

This manuscript investigates how the optical reciprocity constraint, rooted in Planck's law and the detailed balance limit, may be mitigated in semiconductors. Using Bi2S3 as a model system, the authors demonstrate a pseudo-direct bandgap with closely spaced indirect and direct transitions, enabling strong absorption while suppressing bimolecular recombination. This decoupling of absorption and recombination suggests a potential pathway toward improved solar energy conversion beyond conventional limits. Overall, the work presents sufficient novelty and depth to meet the standards of Nature. I therefore recommend acceptance of the manuscript subject to minor revisions.

We are grateful for these positive and constructive comments by Reviewer #3.

1. The authors calculated the joint density of states and electronic band structure of Bi2S3, identifying a lowest-energy direct transition of ~40 meV. This conclusion is indirectly supported by the charge-carrier recombination dynamics in Fig. 3. More direct experimental evidence would be beneficial to quantitatively determine the magnitude of this energy difference.

The Reviewer suggests an experimental investigation of the direct-indirect gap energy split. While such a direct determination would be desirable, its experimental measurement is intrinsically challenging for pseudo-direct semiconductors. As a result of the design principle for pseudo-direct semiconductors, this energy split must necessarily be on the order of only a few $k_B T$ at room temperature. However, the considerable difference in the magnitude of the absorption coefficients for indirect (~10^0-10^2 cm^{-1}) and direct (~10^5 cm^{-1}) transitions unavoidably complicated matters: any broadening of the direct transition (arising e.g. from phonons, disorder, optical interference effects) will therefore conceal the onset of the indirect transitions. For instance, both phonon broadening and the Urbach tail are expected to be in the range of at least a few tens of meV (*J. Phys. Chem. Lett. 2022, 13, 33, 7702–7711*), thus making it intrinsically unfeasible to disentangle the direct and indirect absorption onsets. However, we emphasise that both our computed electronic bandstructure and our experimentally extracted data for k_2 and subsequent van Roosbroeck-Shockley modelling independently indicate a ΔE value of ~40meV, thereby providing indirect but mutually consistent validation of the pseudo-direct scenario.

2. In Fig. 3c, the authors discuss the effect of the valence band maximum energy difference (ΔE) on the bimolecular intrinsic recombination process (k2). However, it is unclear whether the momentum difference (Δk) between the valence band maxima also influences the carrier dynamics. I recommend adding a discussion on this aspect.

The Reviewer raises an interesting point regarding the role of momentum separation between the band extrema in the bimolecular recombination process. To further clarify this point, we note that there is generally a large discrepancy in k_2 rates between direct and indirect semiconductors, which defines two distinct processes arising from the crystal quasi-momentum conservation law $\Delta k = 0$. Here, the momentum carried by an optical photon is negligible (~10^4 cm^{-1} at the Bi_2S_3 band-edge transition) compared with the dimensions of the Brillouin zone (~10^7 cm^{-1} for Bi_2S_3 Brillouin zone), radiative interband transitions can be considered as effectively vertical in k-space. Therefore, when, as in our case, we have a finite $\Delta k \sim 10^7\ cm^{-1}$, the indirect recombination channel always requires phonon assistance, thus making it substantially slower than the direct radiative transition.

Owing to this large discrepancy between direct and indirect channels, in our model, the bimolecular recombination is mainly driven by the direct recombination channel, in the presence of sufficient thermal population. For this reason, ΔE is found to be the key parameter governing the temperature dependence of k_2. Specifically, ΔE controls the thermal population of the higher-lying direct states: at elevated temperature, a non-negligible charge-carrier fraction accesses the direct transition, enabling a faster radiative pathway, whereas at low temperature, charge carriers are increasingly confined to the lower-lying indirect states, suppressing k_2. Therefore, our experimental results indicate that momentum conservation is not the rate-limiting step. Given the discrepancy between the direct and indirect transition rates, we assume that the energy offset ΔE is the rate-limiting step. This rate-limiting role of ΔE assumes that intervalley scattering between the indirect and direct band maxima is fast compared with the radiative lifetime, which is a standard assumption for semiconductors with strong electron-phonon coupling (see *Appl. Phys. Lett. 109, 162102 (2016)*), consistent with the sub-picosecond thermalisation observed in our OPTP measurements. We have now added a section in the Supplementary Note 3 to further clarify this in the, we thank again the reviewer for the insightful point.

The revised Supplementary Note 3 now reads:

"***Role of the momentum offset (Δk) between the band edges.*** *In principle, the pseudo-direct character of* Bi_2S_3 *is not only defined by the ΔE parameter, but also by a momentum offset Δk parameter, which accounts for the different position of the involved band-edges in the first Brillouin zone. Crucially, radiative band-to-band recombination is bound to conserve crystal quasi-momentum k. Owing to the negligible momentum carried by a band-edge photon (* $\sim 10^4$-10^5 cm^{-1} *for band edge transitions in* Bi_2S_3*), with respect to the dimensions of the Brillouin zone (* $\sim 10^7$-10^8 cm^{-1}*), radiative transitions are effectively vertical in k-space (Δk = 0). Consequently, the recombination channel associated with the indirect transition requires the assistance of a momentum-conserving phonon and is therefore orders of magnitude slower than the direct one.*

Crucially, while the Δk value thus determines the direct or indirect character of the recombination, we posit that momentum conservation is not the rate-limiting factor for the temperature dependence of k_2*. Owing to the large discrepancy between the direct and indirect recombination rates, the direct channel dominates recombination whenever it is thermally accessible, and therefore the temperature dependence of* k_2 *is instead governed by the energy offset ΔE, which controls the Boltzmann population of the higher-lying direct states. We note that our hypothesis builds on the assumption that charge carriers maintain a quasi-thermal-equilibrium population across band edges that are closely spaced in energy. The underlying requirement is that intervalley scattering must be fast compared with the radiative lifetime. Importantly, this condition is generally satisfied, as intervalley scattering in semiconductors occurs on the tens of femtosecond timescale(43), which is significantly faster than bimolecular radiative recombination.* "

3. The authors mention that Bi2S3 has significant potential for photoelectric applications and compare its k2 values and absorption coefficient with those of common photovoltaic materials. However, as shown in Fig. 3b, the carrier mobility of Bi2S3 at room temperature is quite low, which would limit its carrier diffusion length. It is recommended that the authors fabricate a prototype device based on Bi2S3 to experimentally validate its potential applications in the optoelectronic field.

The Reviewer raises an interesting point on the potential of these Bi_2S_3 thin films for photoelectric applications. While we agree with the Reviewer that the charge-carrier mobility in our Bi_2S_3 thin films is lower than for some of the highest-performing group III-V and IV semiconductors, such as GaAs and Si ($\sim 10^3$ $cm^2V^{-1}s^{-1}$), we stress it is comparable to that for lead halide perovskites ($few\ 10s\ of\ cm^2V^{-1}s^{-1}$ which now reach PV efficiencies in excess of 27% (https://www.nlr.gov/pv/cell-efficiency), and therefore this moblity value clearly does not preclude the possibility of high-efficiency solar cells.

We agree with the reviewer that the concept of the pseudodirect semiconductor has clear potential to boost the charge-carrier diffusion length through the suppression of radiative recombination. However, in these Bi_2S_3 thin films, the diffusion length is currently still determined by monomolecular recombination at solar injection levels (~10^{15} cm^{-3}). Therefore, while previous work has demonstrated different photoelectric applications of Bi_2S_3 thin films [see Appl. Phys. Lett 122 (19), 2023; Phys. Rev. Applied 19 (6), 064080, 2023; ACS Mater. Lett. 7 (9), 3159-3166, 2025; ACS Photonics 12(1), 457-463, 2024], these applications do not yet reveal the potential of the pseudodirect bandgap character. We have now added a new figure (see Figure R3.1 below) to illustrate how the pseudodirect semiconductor can generate enhanced diffusion lengths once the radiative regime of a well-passivated semiconductor is reached.

However, this would require complete suppression of trap-mediated processes at solar illumination levels. Crucially, for silicon-based solar absorbers such optimisation has taken 7 decades to achieve since the first demonstration of a silicon solar cell in 1954, with operation close to the radiative limit being approached only now (see NREL/NLR Solar Cell Efficiency Chart https://www.nlr.gov/pv/cell-efficiency). For halide perovskites learned experience of the research community has accelerated such time scales to around two decades. Given such timelines, we stress that unless the concept of pseudo-direct behaviour is highlighted now and promising pseudo-direct semiconductor candidates are searched for, such work to optimise these semiconductors will most probably never happen in the first place. Here lies the value and impact of our work on solar-energy conversion, as a trigger to discover candidates and initiate optimisation and implementation.

We have now added a Supplementary Note 5 that summarises these points, and now reads:

"Supplementary Note 5: Impact of pseudo-direct bandgap character on solar energy conversion devices

The central idea of our work is that the pseudo-direct bandgap character, demonstrated here for Bi_2S_3 thin films, can effectively suppress intrinsic bimolecular recombination (up to 75% in Bi_2S_3) while still retaining a remarkably high absorption coefficient. Both these parameters are key factors in determining the efficiency of solar cells and other solar energy conversion devices (e.g., photoelectrochemical cells). Therefore, we discuss the implications of a pseudo-direct character on these devices, based on three main points:

(i) *The charge-carrier lifetime in a semiconductor is generally limited by both trap-mediated monomolecular and intrinsic bimolecular recombination processes (25). As described in Supplementary Note 1, monomolecular recombination processes generally dominate in the low charge-carrier density regime. However, in optimised solar absorbers (e.g., silicon, GaAs, lead halide perovskites), the charge-carrier lifetime is mainly limited by bimolecular recombination processes* $\tau_{bim}=(nk_2)^{-1}$*, as a result of the reduced trap densities (and hence reduced k_1 rates)(41). As discussed in Supplementary Note 3, a pseudo-direct semiconductor such as Bi_2S_3 can achieve a ~75% suppression of the bimolecular recombination rate, with respect to a direct semiconductor with the same absorption coefficient α. As shown in Figure S19, the increase in lifetime translates into a longer charge-carrier diffusion length* $L_D=\sqrt{D\tau}$ *for optimised solar absorbers, which are expected to reduce transport and collection losses (i.e., potentially increasing short-circuit current and fill factor parameters) in devices. We note that, based on the extracted k_1 values for our Bi_2S_3 thin films, the charge-carrier lifetime at solar (AM 1.5) injection levels is dominated by the monomolecular term (see Figure S19). Therefore, this intrinsic advantage is currently latent and would emerge upon defect passivation.*

(ii) *The suppression of the intrinsic recombination channel also has the potential to reduce fundamental losses in a solar cell operating at the radiative limit. These emission-related losses (which include recombination losses and only partially Boltzmann losses) amount to several per cent, depending on the absorber's bandgap: for example, precise calculations based on the original Shockley-Queisser concept have been made by Hirst et al. (53). Beyond improving the efficiency of solar cells in an optimised regime (point (i)), the suppression of k_2 also*

has the potential to circumvent these fundamental limits for pseudo-direct semiconductor-based devices operating at the radiative limit.

(iii) The extended charge-carrier lifetime (discussed above) carries significant implications for applications beyond photovoltaics. Importantly, long charge-carrier lifetimes are crucial for applications in photocatalysis, where longer lifetimes are necessary to enable time for surface chemical reactions to occur. Importantly, such a scheme trading off photovoltage for longer charge-carrier lifetimes is already adopted in nature: biological photosystems sacrifice part of their photovoltage to achieve gains in excited state lifetimes, as explained in Ref.(54) ."

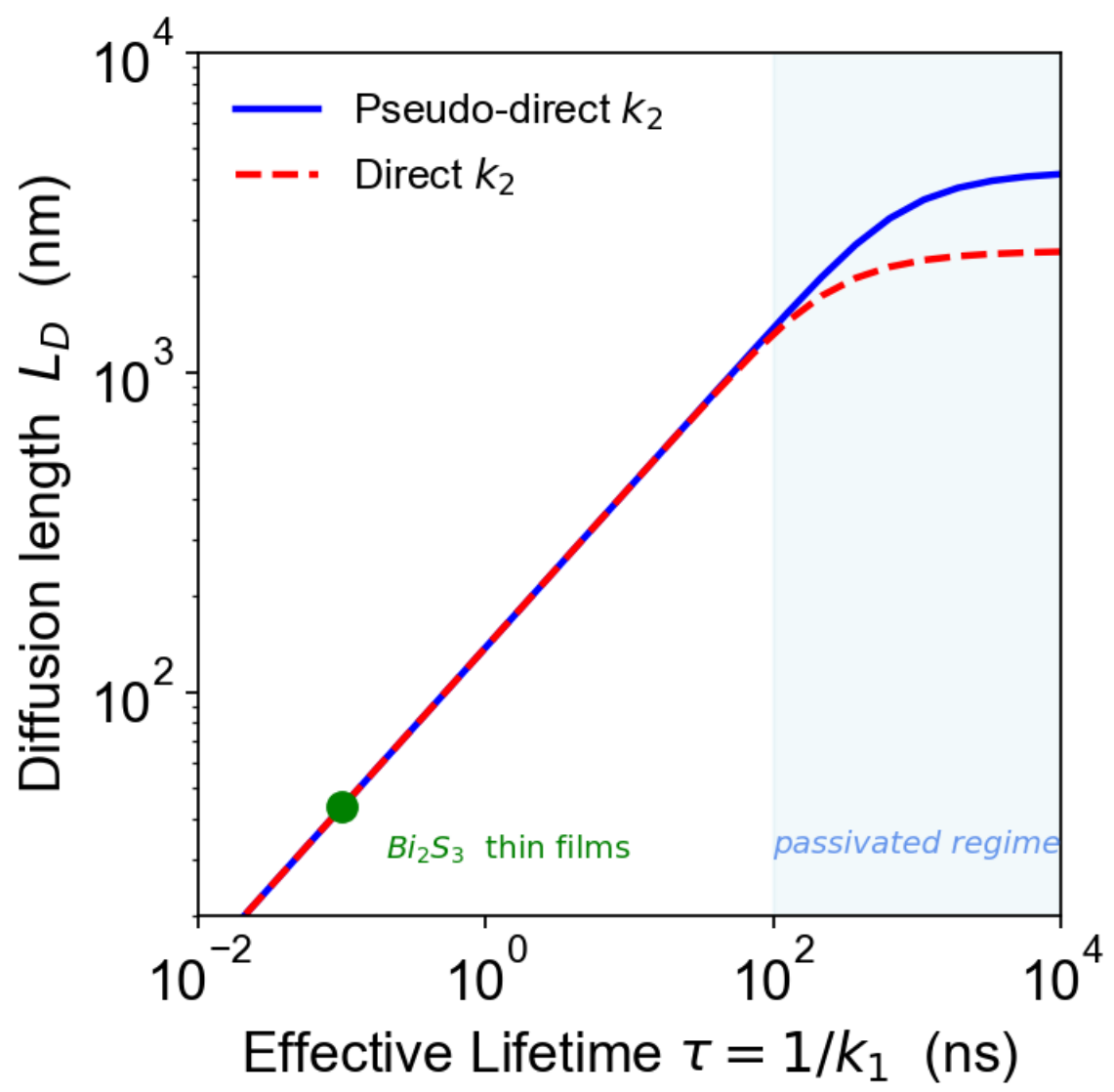

Figure R3.1 | Diffusion length calculations for Bi_2S_3 thin films. Calculated charge-carrier diffusion length as a function of the effective charge-carrier lifetime for a pseudo-direct bandgap character (blue solid line) and for a standard direct bandgap character (red dashed line). The green dot indicates the obtained values for k_1 and k_2 experimentally obtained for Bi_2S_3 films examined in this study. The k_2 value for the pseudo-direct line is value of $k_{2,Bi2S3} = 5 \times 10^{-11}$ cm^3 s^{-1} experimentally determined at 300 K, while for the direct line, we use the extracted 75% suppression, i.e. $k_{2,direct} = 4 \times k_{2,pseudodirect}$. The comparison highlights that the impact of a pseudo-direct bandgap character will only become apparent in the radiative regime (blue shading) for a well-passivated material.

4. The samples are thermally evaporated polycrystalline films. While the authors acknowledge dispersive trapping and selective carrier trapping, the possibility remains that grain-boundary or surface effects contribute to the observed k_2 suppression or to the mobility temperature

dependence. A brief comment (or SI section) comparing to literature single-crystal or colloidal Bi_2S_3 data (or noting why the intrinsic band-structure picture still dominates) would strengthen the claim.

We thank the Reviewer for raising this point about the poly-crystalline nature of Bi_2S_3 thin-film samples. We highlight that we specifically chose optical-pump-THz-probe spectroscopy because this high-frequency AC technique monitors intragrain mobility, which is far less dependent on the microstructure and therefore generally reflects intrinsic mobilities probed over nanometer length scales.

The length scales probed by such linear THz spectroscopy are well documented in literature examining charge-carrier displacement driven by electric fields associated with the THz radiation. Such work has shown that the THz field-driven displacement of charge carriers is intrinsically small within the period of the THz radiation (~1 ps). As discussed in detail by Lloyd-Hughes et al. (*J. Infrared Millim. Terahertz Waves 33, 871 (2012)*), for the low-field regime adopted in our measurements ($E_{THz} < 10^5\ V\ m^{-1}$), the field-induced charge-carrier excursion is below ~2 nm even for high-mobility semiconductors such as GaAs. Therefore, if the excursion length scale is smaller than the grain size of the Bi_2S_3 thin films, such THz conductivity measurements probe intrinsic transport largely unaffected by grain boundaries.

To directly establish this comparison, we have now included in the revised manuscript information on crystalline grain size derived from scanning electron microscopy (SEM) on our Bi_2S_3 thin films. The SEM micrograph (new Figure R3.2, shown below) reveals grain sizes on the order of ~100 nm, which is about two orders of magnitude larger than the THz-drive charge-carrier excursion. Therefore, this experiment probes the intrinsic intra-grain mobility of charge carriers.

As a second important piece of evidence, we note that THz photoconductivity spectra for our Bi_2S_3 thin films exhibit a Drude-like response, i.e. a near-zero imaginary part and near constant real part (see new Figure R3.3 below). Such behaviour reflects the classic presence of a free charge-carrier density with low momentum scattering time. There is no evidence in these spectra of localization or significant backscattering at grain boundaries on the length scale probed (see *J. Infrared Millim. Terahertz Waves 33, 871 (2012), Phys. Rev. B 96, 205439 (2017)*).

Both additional measurements therefore clearly support the assignment of our extracted mobility to the intragrain transport regime that reflects the intrinsic mobility of Bi_2S_3. Moreover, as already stated in the manuscript, this is further confirmed by the large THz-mobility values we obtained here ~15 $cm^2V^{-1}s^{-1}$, which significantly exceed the long-range DC mobility extracted on analogous films in a transistor configuration ~0.01-0.001 $cm^2V^{-1}s^{-1}$ (*Appl. Phys. Lett. 122, 192102 (2023)*).

We therefore reassure the Reviewer that both our technique and our analysis directly probe the intrinsic photophysical processes in our Bi_2S_3 thin films. We thank the reviewer for highlighting that this required clarification and have now added the additional SEM and THz spectral data to the SI, together with further explanations and discussions on this point.

We have added the following new text to the Supplementary Information:

"***Absence of grain-boundary effects on extracted THz charge-carrier mobilities***

The photoconductivity signal obtained by the OPTP technique, which underlies the extracted charge-carrier mobility values, reflects intrinsic intragrain transport and recombination dynamics. As a result, the THz-derived charge-carrier mobilities for Bi_2S_3 thin films (~15 $cm^2V^{-1}s^{-1}$) *are significantly higher than the long-range DC mobilities extracted from Bi_2S_3-based devices (* ~0.01-0.001 $cm^2V^{-1}s^{-1}$ *for Bi_2S_3-based transistors) (48). Such a selectivity of THz spectroscopy in polycrystalline thin films towards intragrain transport originates from the length scales probed in linear THz spectroscopy. In OPTP measurements, the photogenerated charge carriers are displaced by the oscillating electric field associated with the THz radiation. As discussed in detail by Lloyd-Hughes et al. (49), for the low THz-fields analogous to those used in our measurements ($E_{THz} < 10^5\ V\ m^{-1}$), the field-induced charge-carrier excursion is below ~2 nm even for high-mobility semiconductors such as GaAs. Crucially, this excursion length scale is significantly smaller than the size of the Bi_2S_3 grains in our thin films, as shown in Figure S16, thereby implying that THz conductivity measurements probe intrinsic transport largely unaffected by grain boundaries.*

Our hypothesis is further supported by the THz photoconductivity spectra measured for our Bi_2S_3 thin films. Importantly, several works have reported how nanoscale morphologies (for instance, in nanocrystals and quantum dots(47,50)) can yield backscattering processes that

determine a specific phase relation between the oscillating electrons and the THz field, summarised in the Drude-Smith model (51). The effect of surfaces is therefore observed mainly in THz photoconductivity spectra as the emergence of a non-zero imaginary part and a non-monotonous real part of the photoconductivity. Conversely, the THz photoconductivity spectra recorded at 4 K on the photoconductivity peak (Figure S17) exhibit a Drude-like response, i.e. a near-zero imaginary part and a near-constant real part. Such behaviour reflects the classic presence of a free charge-carrier density with low momentum scattering time. Here, the Drude-like behaviour of charge carriers in our Bi_2S_3 thin films confirms the absence of grain-boundary-induced effects. ”

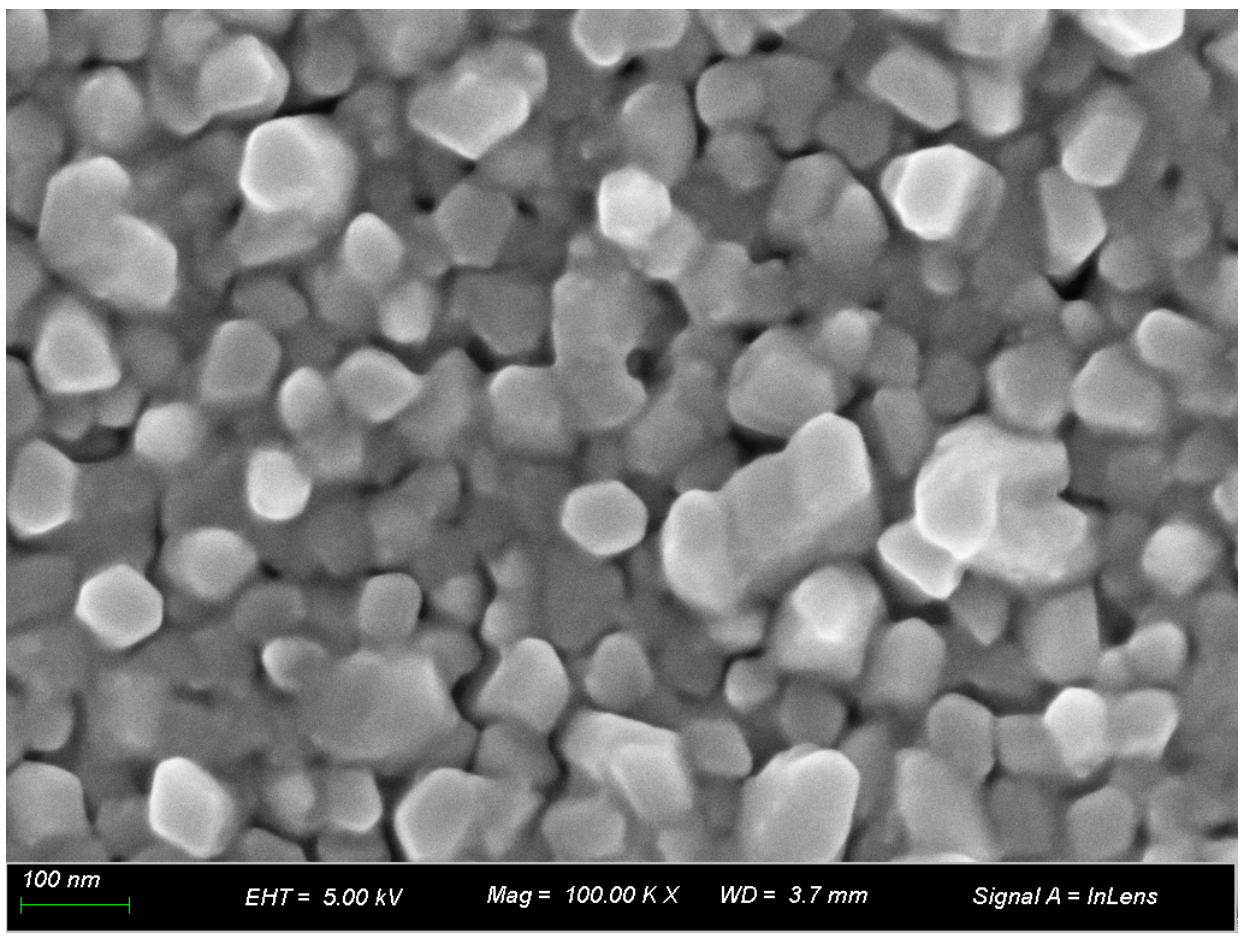

Figure R3.2 | Surface morphology of Bi_2S_3 thin films. The surface morphology of the films was characterised using a Zeiss GeminiSEM 500 field-emission scanning electron microscope (SEM). All measurements were performed under high-vacuum conditions at an acceleration voltage of 5 kV and a working distance of 3.7 mm in secondary electron (SE) mode. The samples possess sufficient electrical conductivity to prevent charging artifacts during imaging, and therefore, no metallic coating was applied. SEM micrographs show grain sizes on the order of ~ 100 nm.

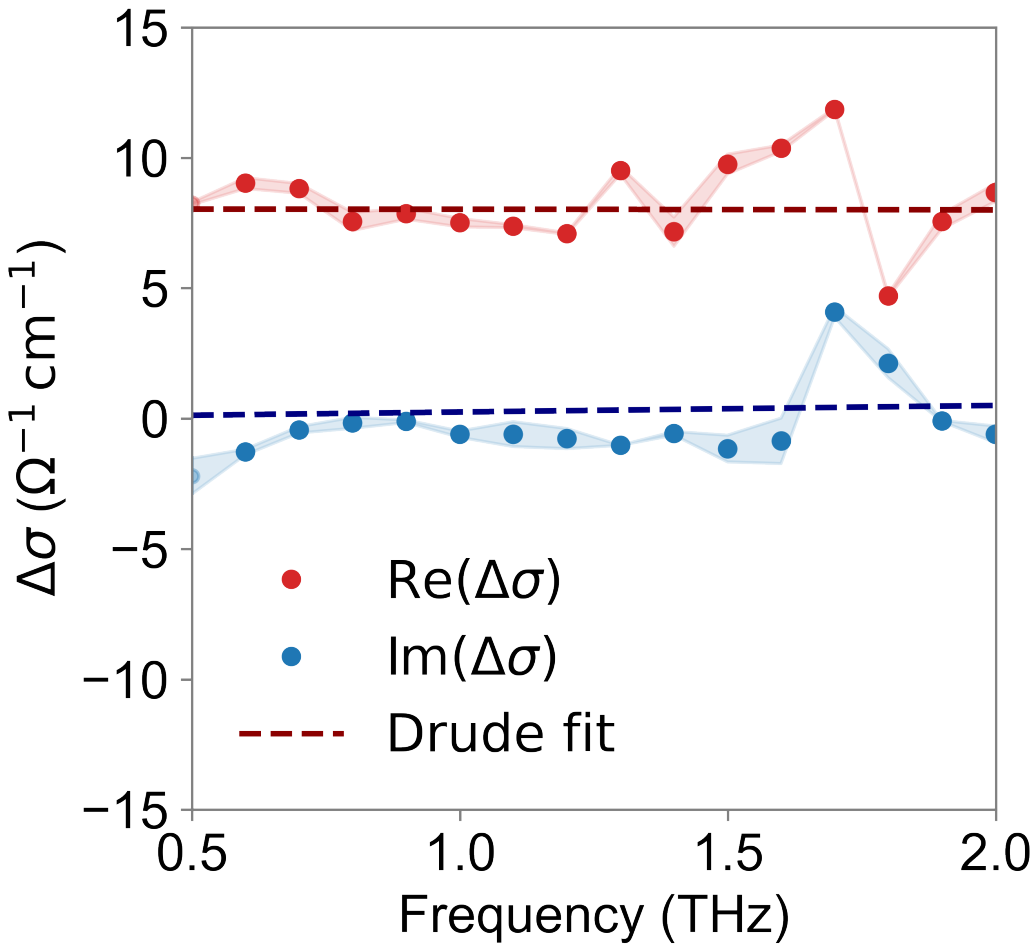

Figure R3.3 | Photoconductivity spectrum for Bi_2S_3 thin films. Real (red) and imaginary (blue) part of the photoinduced THz conductivity spectra for Bi_2S_3 thin films, measured at 4 K around the peak photoconductivity signal (t = 2 ps) following 1.55 eV excitation at a fluence of 15 μJ cm^{-2}. Dots represent experimental data (with shaded areas indicating statistical error), whereas dashed lines are fits according to the Drude model. We note that the near-zero imaginary part and near constant real part reflects the classic Drude picture for a free density of charge carriers with low momentum scattering time, ruling out significant backscattering contributions from grain boundaries.

We have clearly reminded to this Supplementary Note 4 in the Main Text on page 13:

"We note that the THz AC conductivity probes deployed here mostly reflect intra-grain transport (see Supplementary Note 4)."

5. The abstract promises "far-reaching implications for solar energy conversion." The discussion quantifies the ~75 % k2 suppression but does not estimate the resulting gain in detailed-balance efficiency or carrier lifetime under 1-sun illumination. A short paragraph or SI calculation (e.g., using the measured absorption spectrum + reduced k2 in a drift-diffusion or SQ-like model) would make the applied impact concrete.

The Reviewer requests that we add additional clarification on the actual impact of this new concept of a pseudo-direct semiconductor on solar cell devices. We thank the Reviewer for raising this helpful suggestion, and have now added a discussion of the effects of this concept,

in addition to the calculations for the diffusion length already discussed above. The key idea conveyed in our work is that the pseudo-bandgap character of the electronic band structure can suppress the intrinsic bimolecular recombination channel, which has multiple substantial impacts on a solar-energy conversion device that we explain in detail in the text below, which has now been added to the Supporting Information and summarised in the main text.

(i) Suppressing this channel reduces fundamental losses in a solar cell. These losses (which include recombination losses, and only partially the Boltzmann losses) amount to several per cent, depending on the bandgap of the absorber: for example, precise calculations have been made based on the original Shockley-Queisser concept by Hirst et al. (DOI: 10.1002/pip.1024). Suppressing bimolecular electron-hole recombination will allow us to circumvent these fundamental limits for pseudo-direct semiconductor-based devices operating at the radiative limit.

(ii) Reducing k_2 implies an improved mobility-lifetime $\mu\tau$ product, as for optimised solar absorbers operating at or near the radiative limit, the value of τ equals the inverse nk_2 product, where n is the charge carrier density at solar irradiation densities. As a result, the charge-carrier diffusion length is expected to increase, thereby reducing transport losses and improving short-circuit current (J_{sc}) parameters in devices operating below the radiative limit.

(iii) Beyond solar cell applications, the suppression of the nk_2 term implies longer charge-carrier lifetimes. This condition is essential for applications in photocatalysis, where longer lifetimes are necessary to enable time for surface chemical reactions to occur. Importantly, such a scheme trading off photovoltage for longer charge-carrier lifetimes is already adopted in nature: biological photosystems sacrifice part of their photovoltage to achieve gains in excited state lifetimes, as explained in Ref. Nat Rev Mater 7, 503–521 (2022).

We have now added a Supplementary Note 5 that summarises these points, and now reads:

"Supplementary Note 5: Impact of pseudo-direct bandgap character on solar energy conversion devices

The central idea of our work is that the pseudo-direct bandgap character, demonstrated here in Bi_2S_3 thin films, can effectively suppress intrinsic bimolecular recombination (up to 75% in

Bi_2S_3) while still retaining a remarkably high absorption coefficient. Both these parameters are key factors in determining the efficiency of solar cells and other solar energy conversion devices (e.g., photoelectrochemical cells). Therefore, we discuss the implications of a pseudo-direct character on these devices, based on three main points:

(i) *The charge-carrier lifetime in a semiconductor is generally limited by both trap-mediated monomolecular and intrinsic bimolecular recombination processes (25). As described in Supplementary Note 1, monomolecular recombination processes generally dominate in the low charge-carrier density regime. However, in optimised solar absorbers (e.g., silicon, GaAs, lead halide perovskites), the charge-carrier lifetime is mainly limited by bimolecular recombination processes $\tau_{bim}=(nk_2)^{-1}$, as a result of the reduced trap densities (and hence reduced k_1 rates)(41). As discussed in Supplementary Note 3, a pseudo-direct semiconductor such as Bi_2S_3 can achieve a ~75% suppression of the bimolecular recombination rate, with respect to a direct semiconductor with the same absorption coefficient α. As shown in Figure S19, the increase in lifetime translates into a longer charge-carrier diffusion length $L_D=\sqrt{D\tau}$ for optimised solar absorbers, which are expected to reduce transport and collection losses (i.e., potentially increasing short-circuit current and fill factor parameters) in devices. We note that, based on the extracted k_1 values for our Bi_2S_3 thin films, the charge-carrier lifetime at solar (AM 1.5) injection levels is dominated by the monomolecular term (see Figure S19). Therefore, this intrinsic advantage is currently latent and would emerge upon defect passivation.*

(ii) *The suppression of the intrinsic recombination channel also has the potential to reduce fundamental losses in a solar cell operating at the radiative limit. These emission-related losses (which include recombination losses and only partially Boltzmann losses) amount to several per cent, depending on the absorber's bandgap: for example, precise calculations based on the original Shockley-Queisser concept have been made by Hirst et al. (53). Beyond improving the efficiency of solar cells in an optimised regime (point (i)), the suppression of k_2 also has the potential to circumvent these fundamental limits for pseudo-direct semiconductor-based devices operating at the radiative limit.*

(iii) *The extended charge-carrier lifetime (discussed above) carries significant implications for applications beyond photovoltaics. Importantly, long charge-*

carrier lifetimes are crucial for applications in photocatalysis, where longer lifetimes are necessary to enable time for surface chemical reactions to occur. Importantly, such a scheme trading off photovoltage for longer charge-carrier lifetimes is already adopted in nature: biological photosystems sacrifice part of their photovoltage to achieve gains in excited state lifetimes, as explained in Ref.(54).”

Reviewer #4

In their manuscript entitled "Delayed Electron-Hole Recombination in a Pseudo-Direct Bandgap Semiconductor", Righetto et al. discuss the balance between light emission and absorption in the semiconductor Bi2S3 that features an indirect band gap just 40 meV below its direct gap. Since direct-gap absorption and indirect-gap emission after carrier thermalization do not involve the same electronic states, the two are not constrained by Kirchhoff's law. The authors argue that the combination of efficient direct-gap absorption and delayed indirect-gap emission is a beneficial property for future photovoltaic materials to circumvent the Shockley-Queisser efficiency limit. They combine first-principles density-functional theory simulations with luminescence and conductivity experiments as well as carrier-dynamics simulations to substantiate their claims.

As a theorist, I will mainly comment on the theory sections of the present study. The manuscript is physically sound and very well written. The argumentation is conclusive, sheds light on the controversial band-edge physics of Bi2S3, and suggests a convincing mechanism to explain the available literature data as well as the experiments conducted in this work. Due to its potential wider implications for more efficient future photovoltaics, it is of interest to a wide community, and I can recommend publication in Nature provided that the following issues are addressed:

We are very grateful for the positive consideration of our work and for the constructive suggestions provided by Reviewer #4, which we have all implemented.

** In the discussion, the authors claim the charge-carrier recombination mechanism to be "unique" to Bi2S3 thin films. In principle, it should be a quite abundant property present in a number of semiconductors. DFT databases like the Materials Project contain millions of data sets including band-gap data on semiconductors. Shouldn't it be easy to extract other candidate materials with similar band-gap properties as Bi2S3 and identify those with comparable optical properties?*

We agree entirely with the Reviewer. Our intention is not to suggest that Bi_2S_3 is unique (though it does have favourable properties as a solar absorber) but rather to establish experimentally the pseudo-direct concept and identify the governing physical principles, here for the example of Bi_2S_3.

We are in agreement with the Reviewer that the broader applicability of this concept is an exciting and important direction for future work. In the revised manuscript, we now expand the discussion regarding future identification and engineering of pseudo-direct semiconductors. In particular, we propose that high-throughput screening approaches based on large materials databases, such as the Materials Project, could be used to identify candidate pseudo-direct semiconductors by using the direct–indirect energy splitting (ΔE) as a descriptor.

Such an exploration should not only find other materials with a pseudo-direct character but also target materials with properties desirable for specific applications (e.g., bandgap for sunlight absorption, absorption coefficients, effective masses, transport properties, layered structure, electronic dimensionality, defect properties, etc.). We envision a two-phase approach, where an initial high-throughput screening of materials would be based on a value of ΔE~2-3 $k_B T$ as a descriptor to identify possible pseudo-direct candidates in the Materials' Project database. As already mentioned in the Manuscript, based on the available literature, we expect several members of the broad perovskite-inspired materials family, which include several layered materials – e.g., bismuth and antimony halides and chalcohalides – to present this specific electronic structure feature. A second phase of such a study would also implement finer screening methods on the retrieved material space, with the two-fold aim of: (i) refining the ΔE value and select visible range absorber using more accurate hybrid-functional-based calculations on this reduced set and (ii) filter candidates that couple the pseudo-direct bandgap character with other desirable traits, such as high absorption coefficients, low effective masses, electronic dimensionality, and favourable defect properties (i.e. shallow defect character). Therefore, our study establishes the concept of pseudo-direct character and adds a further dimension to the materials search, which can be implemented in broad screening studies.

In the revised manuscript, we now discuss how high-throughput computational screening approaches could identify additional pseudo-direct candidates across large semiconductor databases.

The revised last paragraph in the Discussion section (p. 18-19) now reads:

"*Our modelling (Figure 3C and Figure S15) suggests that either identifying new pseudo-direct semiconductors with targeted ΔE values or implementing approaches to directly control ΔE will be crucial areas of development. We envision a strategy to discover possible pseudo-direct candidates that builds on a two-phase methodology: (i) an initial coarser screening of large*

material databases – e.g., the Materials' Project (72) – to identify candidate semiconductors and insulators showing $\Delta E \sim 2 - 3\ k_B T$, and (ii) a refinement of the candidate pool based on their band gap magnitude calculated using hybrid functionals, and further sift through pseudo-direct candidates for other desirable traits, such as high absorption coefficients, low and isotropic effective masses, and favourable defect chemistries. A second strategy is to control and tune ΔE values directly through external stimuli. For example, we propose that for materials with van der Waals nature of atomic bonding, such as in Bi_2S_3, and similarly transition metal dichalcogenides (73, 74), indium selenide (75, 76), and phosphorene (77), in which direct-indirect character crossovers have been reported, inter-ribbon(or inter-layer) interactions may exploited to tune ΔE. For example, our calculations (Figure S18) reveal that when increasing the distance between $(Bi_4S_6)_n$ ribbons along the a- and c-axis of the Bi_2S_3 crystal structure (i.e., when expanding the lattice), ΔE evolves in the range between ~10 to ~100 meV. We note that in such van der Waals-bonded, layered materials, interlayer interactions may be tuned through exfoliation (78), strain (40), and intercalation (79) strategies, with the potential to directly control intrinsic bimolecular recombination processes in pseudo-direct semiconductors."

** What do the authors consider a good "threshold" to distinguish between an indirect and a pseudo-direct semiconductor at room temperature? In the manuscript, indirect-direct-gap energy differences ranging up to 7 k_B*T at room temperature are discussed. Would, for instance, germanium with a 0.14 eV gap difference still count as pseudo-direct here (e.g. considering the data in Figs. 3C and S14), even though it is usually considered indirect?*

We would not define pseudo-directness as a sharply bounded feature, given that thermal activation does not usually constitute a sharp cut-off but a probabilistic exchange based on temperature. Operationally, the pseudo-direct regime corresponds to a ΔE splitting sufficient to suppress the thermal population of the direct recombination channel, but small enough that strong direct absorption remains for energies close to the absorption onset.

Our modelling suggests that the most effective regime corresponds approximately to ΔE values of a few $k_B T$ (ΔE ~ 2-3 $k_B T$ at room temperature) as suggested by the trends in k_2 suppression as a function of ΔE reported in our Figure S15B. Therefore, an ideal pseudo-direct semiconductor should exhibit a splitting in this range, which would exclude the germanium

semiconductor mentioned by the Reviewer. We note that for higher ΔE values the suppression plateaus, thus implying that the loss in absorption coefficient does not correspond to significant suppression of the recombination. We had included such values in the calculation to explore the full range of such effects, rather than to suggest that $\Delta E = 7\ k_BT$ would make a good pseudo-direct semiconductor.

We thank the reviewer for pointing out that this was confusing and have now made several edits around the discussion point of the model in the SI and the main text to clarify this better to readers.

The revised caption of Figure 3C now reads:

(C) Calculated temperature dependence of the bimolecular recombination coefficient k_2 evaluated by applying the vRS relation to the model presented in Figure 3A. Open circles with different colours represent calculated k_2 values for different $\boldsymbol{\Delta E}$ *values between 30 and 200 meV. The upper end of this range is included to illustrate the convergence of pseudo-direct behaviour towards the conventional indirect limit, rather than to indicate favourable values for a pseudo-direct semiconductor (see **Figure S15B and** Supplementary Note 3). Brown open circles represent the case of a direct semiconductor.*

The revised caption of Figure S15B now reads:

" (B) Calculated k_2 suppression ratio for a pseudo-direct semiconductor plotted as a function of direct-indirect gap splitting ΔE, calculated at temperature T = 300 K. The k_2 suppression ratio represents the fraction of suppressed bimolecular recombination, as a result of the pseudo-direct bandgap splitting. Grey dotted lines represent 50, 90, and 99% suppression ratio values, found at $\Delta E = 24, 65, 127\ meV$, respectively. We note that for larger ΔE values, the calculated suppression saturates, while the range of the indirect absorption character widens, thus resulting in a material progressively reverting to a conventional indirect behaviour (see Figure 3C)."

The revised SI Note 3 now reads:

“Furthermore, we performed different simulations for different direct-to-indirect energy splittings ΔE in the 30-200 meV range and an arbitrarily chosen, fixed $r = 300$. Here, we have deliberately explored an extended ΔE range, which is well beyond the splitting relevant to Bi_2S_3, with the aim of mapping full crossover from direct to pseudo-direct, and finally to conventional indirect behaviour.”

“As shown in Figure S15, within the assumptions of our model, $\Delta E \sim 65\ meV$ can potentially retard the electron-hole recombination by one order of magnitude, ~~and suppression can further reach two orders of magnitude at $\Delta E \sim 127\ meV$~~. Importantly, we observe a saturation effect at high ΔE values ($\Delta E > 100\ meV$) as a result of the direct recombination channel becoming thermally inaccessible, and consequently k_2 approaching the values set by the indirect channel. Therefore, further increases in ΔE yield only marginal suppression, while increasing the range of the absorption dominated by indirect transitions (see Figure S14A) and, with it, the associated photovoltage penalty. Therefore, we note that the benefits of the pseudodirect character are mostly achieved within splittings of a few k_BT.”

Therefore, we propose such a condition ($\Delta E \sim k_B T$) to be defining of a different type of semiconductor, referred to as a pseudo-direct semiconductor, whose identifying features are strong direct absorption coefficients coupled with retarded electron-hole recombination. We stress that the pseudo-direct character definition here is probabilistic rather than a sharp cut-off: ΔE must be large enough for reducing the thermal population of the band-edges associated with the direct recombination, yet ΔE must also be kept as small as possible to ensure efficient absorption via the intense direct transition. As shown in Figure 3D, our fitting further supports the hypothesis that Bi_2S_3 is an exponent of this type of semiconductors. The observed temperature dependent k_2 trend is compatible with the presence of an indirect transition with a two-orders-of magnitude lower absorption coefficient $r \sim 250$ that lies $\sim 38\ meV$ below the direct one.

The manuscript never mentions the suitability of the Bi2S3 band gap for photovoltaics.

We thank the reviewer for raising this point. Bi_2S_3 is a solar absorber attracting increasing attention in the burgeoning field of perovskite-inspired materials (PIMs), owing to its similarity

to other PIMs, such as $AgBiS_2$, BiSI, and others. These are technologically relevant, non-toxic solar absorbers, and specifically, Bi_2S_3 exhibits an absorption onset at 1.4 eV, which is close to the optimum range for single-junction photovoltaics and photocatalysis (see DOI 10.1016/j.solmat.2015.11.019) according to SQ theory. However, we also stress that the main aim of this work is to present and experimentally demonstrate the concept and potential of pseudo-direct semiconductors more generally. As discussed above, the aim of our study is to usher in a new family of materials that can be employed in a wide range of high-efficiency solar-energy conversion applications (e.g., tandem cells, indoor PV, photocatalysis) for which a range of different absorption offsets could be targeted.

Following the Reviewer's comment, we have now specified in the Main Text the excellent suitability of the Bi_2S_3 bandgap for single-junction photovoltaic applications, as below:

"We focus on bismuth sulfide (Bi_2S_3), an emerging non-toxic and stable semiconductor with promising photocatalytic, photodetector, energy-harvesting performance, *and an absorption onset at ~1.4 eV, ideal for single-junction photovoltaics* (35-37)."

** Figure 1D shows the joint density of states as a measure for the absorption properties of the material. However, this can be highly misleading as optical-matrix-element effects are disregarded. Dipole-forbidden excitations would show in the joint density of states, but not contribute to absorption. The authors should present the absorption spectrum instead of the joint density of states, which is standard and doable with negligible additional computational cost.*

The reviewer is correct in pointing out that the calculated joint density of states is not directly comparable to measured optical absorption spectra. In practice, not only dipole matrix elements, but also electron-hole correlations need to be included in the calculation of the linear optical absorption spectrum to facilitate direct comparison with experimental measurements.

Prior calculations performed on a closely related semiconductor, Sb_2S_3 (*Phys. Rev. B (2013) 87, 205125*) showed that the optical absorption spectrum with and without inclusion of electron-hole interactions exhibits very similar lineshapes, and are both in good agreement with experimental measurements. The lineshape of the measured optical absorption spectrum of

Sb_2S_3 is very similar to that of Bi_2S_3 reported here. Furthermore, the calculated joint density of states presented here, exhibits the same gradually increasing lineshape as the experimental optical spectrum of Bi_2S_3 and prior studies of Sb_2S_3, leading us to infer that the spectral lineshape is more likely determined by the joint density of electronic states, rather than the strength of the transition matrix elements and/or electron-hole interactions.

However, we recognise that the point raised by the referee can be straightforwardly addressed by computing the optical absorption spectrum of Bi_2S_3 and comparing it directly with experimental measurements. Therefore, in the revised version of our manuscript, we have now included new calculations for the linear optical absorption spectrum of Bi_2S_3 within the GW+BSE framework, including optical matrix elements, electron-hole interactions, and without including phonon-assisted (indirect) transitions. In order to ensure completeness and reproducibility of our study, we have further expanded the method sections in our Supplementary Information to describe in detail the computational setup of our calculations.

Indeed, as we had expected from prior studies, we find that the lineshape of our calculated optical absorption spectrum does not diverge significantly from the previously calculated joint density of states. Furthermore, our calculated optical absorption spectrum (now shown in the revised Figure 1D) is in remarkably good agreement with experimental measurements, further strengthening the validity of our results.

We have replaced Figure 1 to now include the calculated absorption spectrum instead of the DOS, as shown below:

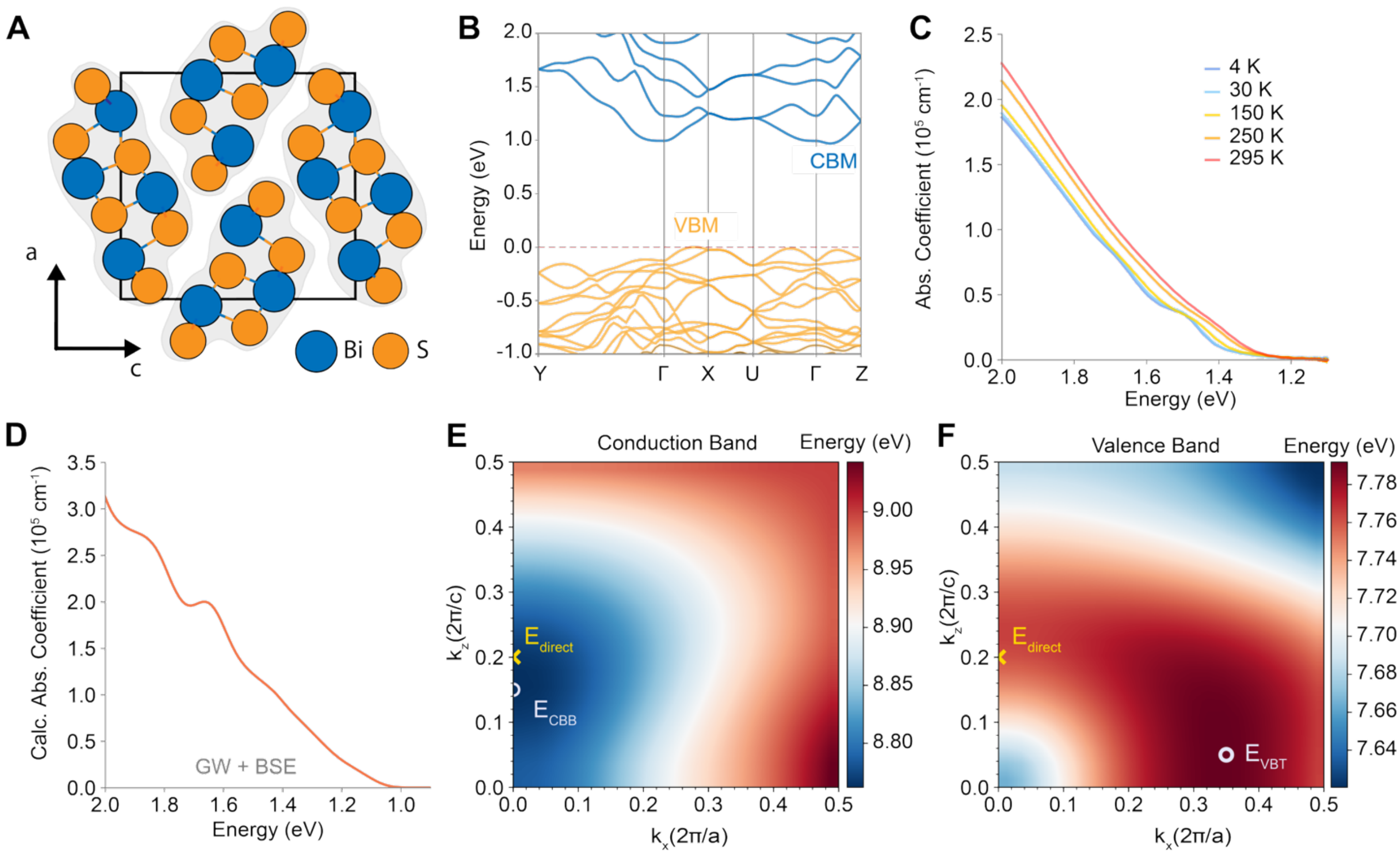

*Figure 1 | Electronic structure and absorption coefficient of Bi_2S_3 thin films. (A) Schematic diagram of the Bi_2S_3 crystal structure (**Pnma** space group) along the [010] direction. The shaded grey regions indicate the ribbon structures. The black square indicates the unit cell, containing two distinct ribbons. Blue and orange spheres represent bismuth and sulfur atoms, respectively. (B) Electronic band structure for Bi_2S_3, as calculated from DFT/PBE+SOC. The zero of the energy axis is set to the top of the valence band and indicated by a red dashed line. Low-energy direct electronic transitions are depicted on the electronic band structure in Figure S3. (C) Absorption coefficient for Bi_2S_3 thin films on quartz, measured in the NIR-Vis range for selected temperatures between 4 K and 295 K. The complete temperature-dependent absorption coefficient dataset is reported in Figure S4A. (D) Calculated linear optical absorption spectrum including only direct transitions, computed within the GW+BSE framework, including SOC, as described in detail in the SI. The onset of the calculated spectrum is red-shifted with respect to the measured onset by approximately 0.3 eV, consistent with typical underestimated quasiparticle band gaps computed within the G_0W_0 with a DFT/PBE starting point, extensively documented in the literature(58). (E), (F) Calculated DFT/PBE (including SOC) electronic bandstructure k_x-k_z on the BZ plane: Y(0, 1/2, 0) - Γ(0, 0, 0) - X(1/2, 0, 0) - U(1/2, 0, 1/2) - Z(0, 0, 1/2), in units of the reciprocal lattice, highlighting the reciprocal space location of the valence band maximum and the conduction band minimum,*

where a, b, and c are the Bi_2S_3 lattice vectors. White circles are used to indicate the position of the valence band maximum (VBM) and the conduction band minimum (CBM). A yellow mark is used to indicate the position

We have added further information on the methodology to the Supplementary Information as follows:

"GW/BSE calculations

We calculate the quasiparticle band structure of Bi_2S_3 within the single-shot G_0W_0 approximation (11), as implemented in the BerkeleyGW code (12, 13), with a DFT/PBE starting point, including spin-orbit coupling. We approximate the frequency-dependent dielectric function using the Godby-Needs plasmon pole model(14). We use a dielectric function cutoff of 6 Ry, and sum over 600 bands (408 unoccupied) with a Γ-centered coarse grid of 4x8x4 to calculate the quasiparticle self-energy corrections. These parameters are consistent with prior work(15).

We have checked numerical convergence of the band gap with respect to the number of bands and dielectric function cutoff by testing parameters between 300 and 800 bands, and sweeping the cutoff between 6 Ry and 16 Ry, as shown in Figure S1. We found that within this range, the quasiparticle band gap at Γ converges slowly, and changes by less than 50 meV, as shown in Figure S1. In order to contain the computational effort for subsequent BSE calculations, and given that band gaps appear to vary slowly with the increase of these two parameters, we have chosen to restrict the number of bands to 600 and the polarizability cutoff to 6 Ry. Furthermore, we have also checked convergence with respect to the density of the k-point grid, reported in Table S1.

K-point grid	*Number of bands*	*Polarizability cutoff (Ry)*	*Band gap at Γ (eV)*
2x2x4	*800*	*12*	*1.24*
2x4x2	*800*	*12*	*1.27*
2x4x2	*600*	*6*	*1.24*
4x8x4	***600***	***6***	***1.24***

Table S1 | Convergence of the quasiparticle band gap at Γ with respect to k-point grid. *All grids tested are centered at Γ. Values highlighted in bold are the parameters used in the final calculation.*

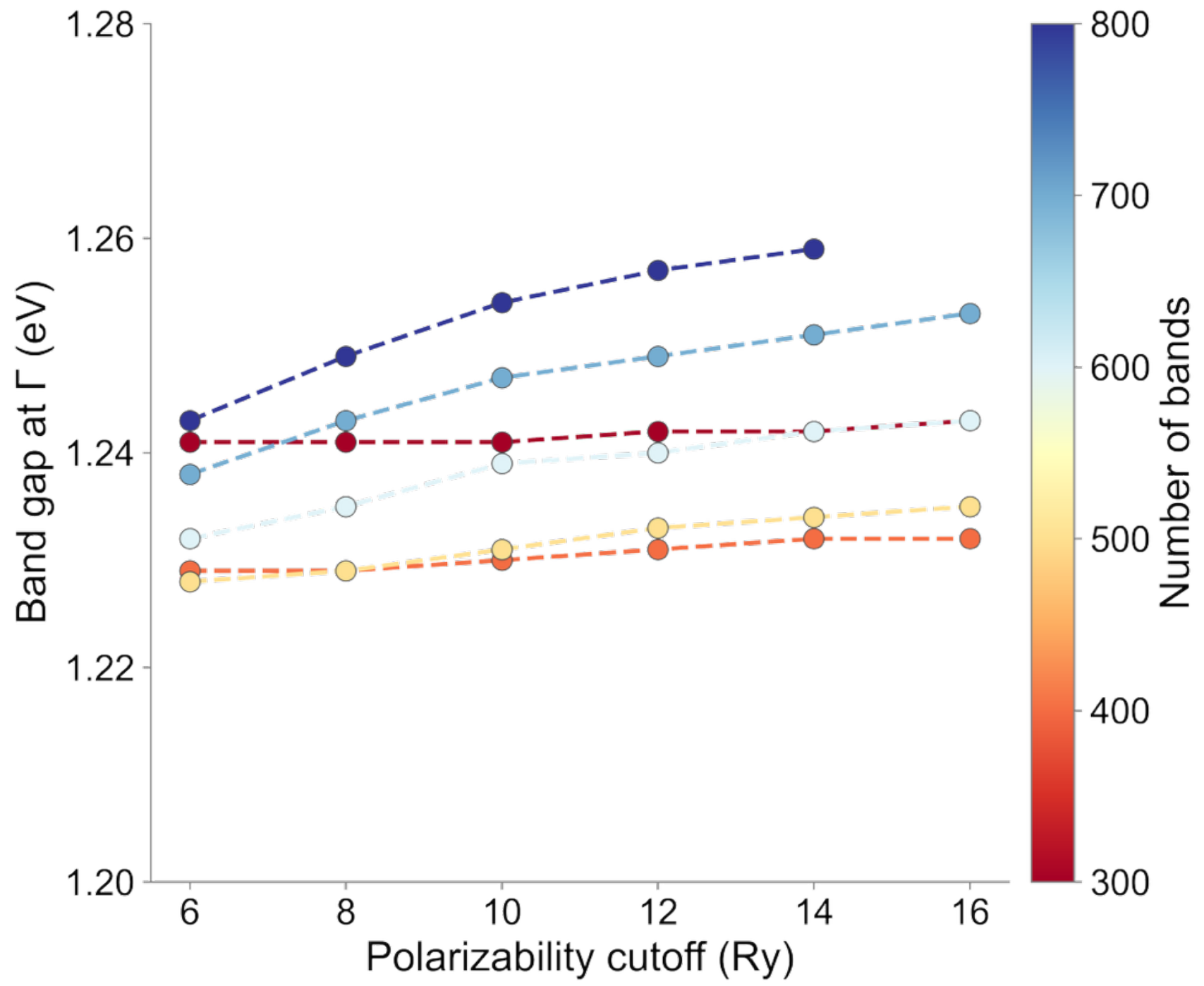

Figure S1 | Convergence of the quasiparticle band gap at Γ with respect to the polarizability cutoff and the number of bands. *Calculations are performed using a Γ-centered kpoint grid of 2x2x4.*

We calculate the linear optical absorption spectrum of Bi_2S_3 by solving the Bethe-Salpeter Equation (BSE)(16), as implemented in the BerkeleyGW code. Calculation of the optical absorption spectrum includes only direct transitions, i.e., does not account for phonon-assisted processes. We calculate the BSE kernel on a Γ-centered grid of 4x8x4, including 12 valence and 16 conduction bands. We interpolate this kernel on a fine, Γ-centered grid of 8x16x8, before we diagonalize the BSE hamiltonian, and compute the imaginary part of the dielectric function, including 8 valence and 12 conduction bands. This computational setup yields

energies of the first excited state which are converged within less than 50 meV, as shown in Table S2."

Coarse k-point grid	*Valence bands (coarse grid)*	*Conduction bands (coarse grid)*	*Fine kpoint grid*	*Valence bands (fine grid)*	*Conduction bands (fine grid)*	*1st excited state energy (eV)*
2x4x2	*12*	*16*	*4x8x4*	*8*	*12*	*1.131*
2x4x2	*12*	*16*	*4x8x4*	*8*	*16*	*1.130*
2x4x2	*12*	*16*	*8x16x8*	*8*	*12*	*1.133*
2x4x2	*16*	*16*	*4x8x4*	*12*	*12*	*1.133*
2x4x2	*28*	*20*	*4x8x4*	*24*	*16*	*1.138*
4x8x4	***12***	***16***	***8x16x8***	***8***	***12***	***1.124***

Table S2 | Convergence of the energy of the first excited state with respect to coarse and fine k-point grids, and the number of valence and conduction bands included in each calculation. *All grids tested are centered at Γ. Values highlighted in bold are the parameters used in the final calculation.*

We calculate the optical absorption spectrum using the expression (17) $\alpha(\omega) = \frac{\omega}{c}\sqrt{2\left(\sqrt{\varepsilon_1(\omega)^2 + \varepsilon_2(\omega)^2} - \varepsilon_1(\omega)\right)}$*, where ω is the photon frequency,* ε_1 *and* ε_2 *are the real and imaginary parts of the dielectric function calculated as described above, and averaged over polarization directions. The optical absorption spectrum reported in Figure 1, is calculated using an arbitrary broadening of 50 meV.*

** su: According to the main text (p. 7), it is located at 0.5*pi/c. According to Figs. 1E,F and S4, it is located at 0.2*2*pi/c = 0.4*pi/c. And according to Table S2, it is located at 0.33*pi/c, provided that the "PBE (dense grid)" setting is the relevant one which is stated nowhere but seems to be the case, as the data for this grid have apparently been used for the conduction-band minimum and the direct-indirect-gap energy difference. How many grid points (not irreducible points) were used for the dense grid? Further, the statement "the valence band top (Figure 1F) is found near the Gamma-U direction ..." (p. 7) is in contradiction to the given coordinates and Fig. 1F where the valence-band maximum is rather close to Gamma-X.*

We thank the referee for pointing out this inconsistency. For clarity, we confirm that the CBM is located at (0,0,0.2) in units of the reciprocal lattice, consistent with Figure 1E,F and Figure S4 (now S5 in the revised SI). We have corrected this throughout the main text and SI. The data in Table S2 (now S5 in the revised SI), and Figure S5 was obtained using a grid of 10x1x10, which locates the direct gap at (0,0,0.2). We have now corrected this detail in the new Table S3. Furthermore, we have further expanded Table S3 to include newly computed GW band gaps, which confirm our prior observations.

Manuscript preparation issues:

** Undefined abbreviations: LHP (p. 3), RT (p. 13), FD (legend of Fig. S13C, D).*

We thank the Reviewer for the careful reading of our manuscript. These abbreviations have now been defined.

** It may be a matter of personal taste, but I consider Fig. S2 much more insightful than Fig. 1B.*

Following the Reviewer's comment, we have added a pointer to draw the attention on Figure S2 (now Figure S3) in the caption of Figure 1b.

** Caption of Fig. 1E, F: There is no BZ path, but a plane in the BZ shown in the figure. There is also no yellow cross in the figure, rather something like an angle.*

We thank the Reviewer for raising this point, the caption has been corrected to address this.

** The y-axis label in Fig. 2B, C, D is not explained.*

We thank the Reviewer for raising this point, the label has now been explained in the caption.

** "... the higher-lying valence band minima ..." (p. 14) should probably read "valence band maxima".*

We thank the Reviewer, this has now been corrected.

** In the caption of Fig. 3A, it reads "a two valence bands".*

We thank the Reviewer, the caption has been corrected.

** Missing units for k coordinates in Table S3.*

The units have been added.

** In Eq. (S6), k_b should read k_B.*

This typo has now been rectified.

** Below Eq. (S2), it reads "Here i is the recombination order" even though there is no i.*

We thank the reviewer for spotting this, this typo has been left behind from a previous formulation of the same equation and has now been deleted.

Response to Second Review of Nature Manuscript 2026-03-06751 "Delayed Electron-Hole Recombination in a Pseudo-Direct Bandgap Semiconductor" by Righetto et al.

We are very grateful for the highly positive feedback on the second version of our manuscript, and for the thoughtful and constructive comments provided by the Editor and all the five Reviewers. While Reviewers 3-5 sign off on publication, Reviewers 1-2 still raise a few helpful points on which they seek clarification. We have now carefully implemented all their points, and we thank them for helping us to further improve the clarity, scope, and presentation of the work. We have also addressed the editorial points raised by Dr Li: we have shortened the manuscript and title to the required length and provided references in the abstract. We hope that you may now find the revised version of our manuscript ready for publication.

Below, reviewers' comments are given in *green italics*, our responses are given in black, and our highlighted revisions in the manuscript are given in *blue italics*. Deleted parts are given in ~~*brown italics*~~.

Editorial Comments

(1) Titles cannot exceed 75 characters (including spaces); they must not contain punctuation.

We have revised the title to "Delayed Electron-Hole Recombination in a Pseudo-Direct Semiconductor", which is compliant with Nature guidelines.

(2) Your abstract should be no longer than 230 words. The current length is ok, but please include at least 1 reference.

We thank the editor for their guidance. The abstract paragraph now includes four references citing the established results on which our work builds (listed below). We have also slightly reworded the surrounding sentences to integrate these references better.

1. Kirchhoff's 1860 demonstration that emissivity and absorptivity are equal for a body in thermal equilibrium (*Philos. Mag. 20, 1-21 (1860)*)
2. the original van Roosbroeck and Shockley paper, cited where this principle is applied to semiconductors in the form of the vRS equation (*Phys. Rev. 94, 1558 (1954)*)

3. Würfel's work introducing the generalised Planck's law (*J. Phys. C Solid State Phys. 15, 3967-3985 (1982)*)
4. Davies *et al.,* which demonstrates the inverse absorption process nature of bimolecular recombination in a conventional semiconductor (*Nat Commun 9, 293 (2018)*).

(3) Your paper contains ~4,000 words, which is over our word limit. Please reduce the length of the main text to a maximum ~3,500 words. Technical details that are not central to the main message of the paper, must be moved the Methods section or Supplementary Information.

We have shortened the manuscript and moved parts that are less central to the core message of the paper to the Supplementary Information. The current version of the manuscript has been shortened from ~4,000 to ~3,500 words. We have shown all changes in the tracked versions of the main manuscript and SI, provided as a joint pdf as part of the resubmission.

Reviewer #1

Compared with the initial version, the revised manuscript is more logically coherent, and the explanations of the physical mechanism are clearer and more convincing. The concept of a pseudo-direct bandgap semiconductor, in which strong direct-like optical absorption is decoupled from suppressed bimolecular recombination, remains intriguing and potentially significant for both semiconductor physics and photovoltaics. Regarding the presentation of error bars and statistical information, I have examined the relevant figures (Figure 3B, Figure 3D in the main text, and Figure S13 in the Supplementary Information). In all cases, the error bars are appropriately defined in the corresponding figure legends and represent uncertainties arising from experimental reproducibility and data processing. Nevertheless, several issues remain that should be addressed.

We thank the Reviewer for acknowledging the improvements in the revised version of our manuscript and for once again highlighting the value of our discovery of delayed recombination in pseudo-direct semiconductors.

1.One issue throughout the manuscript is the use of several different quantitative metrics to describe how much bimolecular recombination is "suppressed", yet these metrics are not

clearly differentiated. The vRS model predicts ~75% suppression (i.e., k2 is 4 times lower) at room temperature for ΔE = 40 meV, relative to a hypothetical direct semiconductor with the same direct-gap properties. This is stated in the Discussion. However, the authors also compare the experimental k2 of Bi2S3 (~5 × 10-11 cm3 s-1) with those of lead halide perovskites (6–12 × 10-10 cm3 s-1) and GaAs (~7 × 10-10 cm3 s-1), implying 12–24-fold and 14-fold differences, respectively. These are cross material comparisons and do not reflect a suppression factor within Bi2S3 itself. In addition, the Discussion states that lifetimes can be extended by "up to two orders of magnitude" for pseudo-direct semiconductors in general (with larger ΔE < 100 meV), not specifically for Bi2S3. Given the coexistence of these three distinct frames of reference, the authors should explicitly clarify what each value represents and avoid conflating them. A concise summary table or a dedicated explanatory paragraph would help readers distinguish these metrics.

We thank the Reviewer for raising this point regarding the discussion of the k_2 suppression observed in Bi_2S_3. We fully agree with the reviewer that the provided values for metal halide perovskites and GaAs are not directly comparable with those of Bi_2S_3 but this had not been the purpose of their being mentioned. What we had wanted to convey is that typical direct semiconductors (such as GaAs) display **both** a lower absorption coefficient **and** a higher k_2 than Bi_2S_3. According to the van Roosbroeck-Shockley (vRS) relation, this would be impossible if Bi_2S_3 also were to be a simple direct semiconductor, because the absorption and emission rates for electron-hole transitions are linked by the principle of detailed balance. Therefore, this comparison already shows that Bi_2S_3 deviates from the simple vRS picture for a direct semiconductor.

Regarding the link of k_2 with particular values of ΔE, this is something directly derived from our model discussed in the text. While the suppression value for Bi_2S_3 is specifically calculated for this material only, through our vRS modelling, we directly discuss that other materials may be searched for, whose ΔE values may be directly predicted and engineered to result in the desired suppression of k_2.

That said, we thank the reviewer for highlighting that the discussion was confusing, and, given that the editor has also requested that the manuscript be shortened, we have now significantly

cut the detailed discussion of k_2 values for perovskites and GaAs and simply captured the core idea in one sentence. The revised first paragraph at p. 9 now reads:

"By globally fitting this model to the fluence-dependent THz conductivity, we extract a bimolecular recombination coefficient $k_2 \sim 5 \times 10^{-11}$ cm^{3}s^{-1} at room temperature. ~~*We note that this value is lower than those generally reported for other direct semiconductors, such as lead iodide perovskites ($k_{2,LHP} \sim 6-12 \times 10^{-10}$ cm^{3}s^{-1}) (32, 60, 61) and GaAs ($k_{2,GaAs} \sim 7 \times 10^{-10}$ cm^{3}s^{-1}) (62). In these direct semiconductors, bimolecular recombination has been proven to be an inverse absorption process, and therefore, efficient bimolecular recombination is linked with their high absorption coefficient (up to $\alpha \sim 10^4$ cm^{-1} around the band-edge and across a large part of the visible spectrum (60))*~~. *Bi_2S_3 shows a lower k_2 rate constant than those of direct semiconductors such as GaAs and lead halide perovskites,(30, 53, 54) despite exhibiting a significantly higher absorption coefficient ($\alpha \sim 10^5$ cm^{-1}), thus indicating a suppression of k_2 according to the vRS theory.*"

2. The authors have substantially expanded the Discussion to address generalizability and have outlined a two-step high-throughput screening strategy using ΔE as a descriptor. This is helpful and well-reasoned. However, the discussion would be considerably strengthened if the authors could provide even a preliminary quantitative comparison with known layered materials that have been proposed to exhibit similar pseudo-direct character.

We thank the reviewer for acknowledging the value of our proposed strategy to widen the search for pseudo-direct semiconductors. We have now provided a preliminary quantitative estimate of the k_2 suppression, according to our model, for two layered materials (WSe_2 and InSe) that show a similar pseudo-direct bandstructure.

The revised conclusion paragraph (p. 19) now reads:

"A second strategy is to control and tune ΔE values directly through external stimuli, for instance in materials with van-der-Waals nature of atomic bonding, such as in Bi_2S_3, and similarly transition metal dichalcogenides (63,64), indium selenide(65,66), and phosphorene

(67), whose direct-indirect splitting, e.g., $\Delta E \sim 60\ meV$ *for* WSe_2 *and* $\Delta E \sim 50\ meV$ *for InSe, could potentially yield* k_2 *suppression by 88 and 81 %, respectively, according to our model. In these materials, inter-ribbon (or inter-layer) interactions may further be exploited to tune* ΔE.*"*

Reviewer #2

The authors have made a substantial effort to address my previous concerns, including the addition of SEM characterization, frequency-resolved THz conductivity data, expanded kinetic analysis, and further discussion of the implications for solar-energy conversion. These additions improve the manuscript. However, I do not consider some comments were sufficiently resolved.

We thank the reviewer for the constructive scientific feedback on our investigation and the revised manuscript. We have addressed their remaining points as follows below:

Regarding comment 5: The added SEM image (Figure S16) and THz photoconductivity spectrum (Figure S17) support the conclusion that the measured THz mobility predominantly reflects intragrain transport and that strong grain-boundary backscattering is not evident within the measured THz response. This substantially addresses the transport-related concern. However, supplementary note 4 extends this conclusion to intrinsic intragrain transport and recombination dynamics and states that the Drude-like response confirms the absence of grain-boundary-induced effects. Figures S16 and S17 directly support the intragrain nature of the THz transport, but they do not by themselves establish that the recombination dynamics or broadened absorption onset are unaffected by structural disorder, defects, surfaces, or interfaces. Please restrict the conclusion to what is directly supported by Figures S16 and S17 and explicitly acknowledge that possible microstructural effects on recombination and optical broadening cannot be fully excluded.

We thank the Reviewer for raising this point and giving us the opportunity to further clarify our methodology. The reviewer is correct that the SEM image and THz photoconductivity spectra solely demonstrate that the mobility is governed by intragrain transport. However, the points raised on non-radiative recombination and gradual onset of the absorption spectrum had already been covered by other experimental evidence presented elsewhere in the manuscript.

First, regarding the Reviewer's concern on whether other effects might give rise to the observed gradual onset of the absorption onset: we note that as part of the previous revision round, we had provided a calculated linear optical absorption spectrum, computed within the GW+BSE framework (see Figure 1D in the main text), that well reproduces the observed absorption onset,

based on our bandstructure calculations revealing the pseudo-direct nature of Bi_2S_3. This finding shows that the gradual onset is directly caused by the pseudo-direct nature of Bi_2S_3. We note that the first version of the manuscript had only reported the density of states, which may be the reason this has been missed by the reviewer in this round. Figure 1C and 1D in the first revision of the manuscript now allow for direct comparison between data and computational absorption spectra, highlighting how the pseudo-direct nature of the bandgap causes the shallow onset.

Second, regarding the role of structural disorder, defects, surfaces and interfaces on recombination dynamics, the Reviewer is correct in noting that the SEM and THz photoconductivity spectrum confirm the intragrain nature of transport probed by our OPTP measurements. Importantly, however, we had never claimed that structural disorder, defects, surfaces and interfaces do not play a role in the overall recombination dynamics. Rather, our method based on the k_1k_2 modelling of fluence-dependent OPTP transients (described in Supplementary Note 1) is able to differentiate between defect-induced dynamics (captured by k_1) and bimolecular electron-hole recombination (captured by k_2).

To address the Reviewer's concern, we have inserted a comment in the SI where the SEM and THz photoconductivity spectra are being discussed (SI Note 4) to ensure that readers understand that this solely refers to transport properties. In this note we now point the reader to the SI Note 1, which already discussed how we have accounted for and separate the contribution of trap-mediated recombination from defects from those originating from intrinsic bimolecular recombination. The last paragraph of this discussion in Supplementary Note 4 (pp. 36-37 of SI) now reads:

"*Crucially, this excursion length scale is significantly smaller than the size of the* Bi_2S_3 *grains in our thin films, as shown in Figure S16, thereby implying that THz conductivity measurements probe intrinsic transport largely unaffected by grain boundaries. While THz mobilities are extracted from the peak of the OPTP signal at early sub-ps timescales, we note that the subsequent OPTP dynamics are still generally influenced by defects, surfaces, and interfaces. For this reason, as described in Supplementary Note 1, we model the fluence-dependent OPTP dynamics as a sum of contributions from a defect-related monomolecular term (*k_1*) and a rate constant arising from intrinsic bimolecular recombination (*k_2*), and are therefore able to differentiate between these contributions.*"

Regarding comment 6: The expanded analysis in Supplementary Note 1 appropriately explains that the faster decay at higher fluence in Figures 2B and 2D is inconsistent with a simple trap-saturation scenario. This supports the presence of a substantial second-order recombination contribution. Nevertheless, the statements that disorder- and trap-mediated processes cannot reproduce the observations and that the data unambiguously identify intrinsic bimolecular recombination are too categorical. The manuscript itself shows significant selective trapping and dispersive monomolecular recombination in Figure 2A and Figure S7. Please revise these statements to specify that the data exclude a simple trap-saturation explanation, rather than all possible defect-coupled kinetics. The authors should also clarify whether the fitted k2 is intended as a microscopic band-to-band recombination coefficient or an effective second-order coefficient extracted from the mobile-carrier THz signal.

We thank the Reviewer for raising this point on the role of traps in the reported OPTP recombination dynamics.

We apologise if the phrasing used in our Supplementary Note 1 regarding our k_1k_2 modelling was unclear and thank the reviewer for pointing this out to us. We have revised the final section of this note accordingly, which now reads:

"Crucially, such fluence-dependent behaviour is incompatible with two main mechanisms often invoked to explain charge-carrier-density-dependent dynamics related to defect-mediated recombination, namely disorder-broadened monomolecular recombination and trap saturation. On the one hand, the effect of disorder is fully captured in our model by the KWW formulation of the k_1 parameter, as disorder mainly broadens the distribution of monomolecular rates but does not change the order of the rate law. On the other hand, in the presence of finite trap densities, photogenerated charge carriers can fill the available trap states in a process known as trap saturation (32-34). The result of this process is an effectively suppressed monomolecular recombination rate. It is worth noting that trap-saturation effects yield slower decays at higher fluence, which is the opposite behaviour to what is observed in our Bi_2S_3 OPTP transients. Therefore, the observed acceleration of photoconductivity decay at

increasing excitation fluences excludes trap saturation as the origin of the fluence dependence and requires a second-order term in the rate law."

The Reviewer also asks for further clarification on the nature of the extracted k_2 parameter from our model. Non-radiative second-order recombination is rarely observed in inorganic semiconductors, but cannot be ruled out in principle. However, for exactly this reason, we had already examined the changes in photoluminescence (PL) intensity as a function of time and temperature, which had been reported in Figure S8 and S9. Our findings strongly indicate that the observed bimolecular dynamics is related to radiative bimolecular recombination, as we observe the presence of PL only at charge-carrier densities higher than 10^{17} cm^{-3}, see Figure S8B. At such, increasing contribution from second-order processes begin to dominate and lead to PL emission – the hallmark or a radiative process. A second direct proof of this effect is through temperature-dependent prompt PL, which we had already measured and presented in Figure S9. Again, qualitatively the same T-dependence of PL intensity are observed as for k_2, showing that this is a radiative effect (though somewhat weaker in magnitude because within the (~ns) time-resolution of the experiment, some nonradiative k_1 contributions already occur). We are sorry that these already present evaluations were not directly apparent to the reviewers and hope that our explanations are able to address their concerns.

Regarding comment 7: Supplementary Note 5 and Figure S19 provide a useful discussion of carrier lifetime and diffusion length. The authors also appropriately acknowledge that, in the present Bi2S3 films, recombination under solar injection conditions remains dominated by the monomolecular term. However, my original comment 7 specifically requested either device-level validation or a quantitative estimate of the expected quasi-Fermi-level splitting or open-circuit voltage. Figure S19 presents a diffusion-length analysis but does not provide either quantity. Therefore, this part of the comment remains unresolved. Please provide a quantitative estimate of the quasi-Fermi-level splitting or radiative Voc for the pseudo-direct and corresponding direct-gap cases, including the effect of delta E. Alternatively, the manuscript should moderate claims concerning device-efficiency gains and clearly state that the voltage and efficiency benefits remain prospective rather than demonstrated in the present Bi2S3 films.

We thank the reviewer for this comment. As we had outlined in the previous response, a device-level validation would require an improvement in material defect density of some orders of magnitude. Crucially, for silicon-based solar absorbers, such optimisation has taken 7 decades to achieve since the first demonstration of a silicon solar cell in 1954, with silicon solar cells operating close to the radiative limit, which is only now being approached. Therefore, given the scale of the improvement required, such device-level validation is not currently feasible.

Regarding theoretical predictions of V_{oc} achievable in a PV device based on this approach, we consider that applying the established framework based on optical reciprocity to extract quasi-Fermi level splitting would not lead to meaningful conclusions, given that the pseudo-direct semiconductor concept circumvents the underlying principles. We further note that our calculation of the diffusion length is highly important because this concept is relevant not only to PV applications, but also to photocatalytic materials for which diffusion length matters greatly. Therefore, we feel that a quasi-Fermi level approach would be less useful here.

We fully acknowledge, as we had in the previous response, that no devices have been demonstrated or claimed in our study. We are, however, more than happy to follow the Reviewer's suggestion to make this even more evident, i.e., by clearly stating again that the voltage and efficiency benefits remain prospective, as requested. We have now modified the main text accordingly:

In the Abstract and Introduction:

"*The resulting combination of a high absorption coefficient and delayed recombination offers the unprecedented possibility of overcoming limitations associated with Planck's law, with far-reaching ~~implication~~ potential for solar energy conversion.*"

In the Conclusion (pp. 18)

"*Beyond prospective conversion efficiency gains in sunlight harvesting, modulating the charge-carrier recombination lifetime in highly absorbing semiconductors ~~will~~ also has the potential to unlock new photocatalytic applications where longer timescales (e.g., multiple electron transfer reactions, reactants turnover time) are involved.*"

In the Conclusion (pp. 19):

"*In conclusion, our findings reveal the unique nature of charge-carrier recombination in Bi_2S_3, which embodies the features of pseudo-direct semiconductors, a concept that will not only set a new benchmark in materials discovery but also offer ~~unprecedented~~ prospective opportunities for substantial advances in solar energy conversion applications.*"

Reviewer #3

The revised manuscript has been thoroughly improved in accordance with the reviewers' feedback. The authors have adequately clarified the previous points of concern, and the added discussions are satisfactory. Consequently, I consider the manuscript well-suited for publication and recommend its acceptance.

We are grateful for these positive and helpful comments by Reviewer #3.

Reviewer #4

The authors have addressed all criticisms thoroughly and amended and extended the manuscript with further discussions where appropriate. I consider the manuscript now suitable for publication in Nature.

We are very grateful for the positive consideration of our work and for the constructive suggestions provided by Reviewer #4, which we have all implemented.

I would just like to raise a minor point regarding the newly added GW/BSE calculations for the optical absorption. First of all, the calculated absorption spectrum matches the measured absorption data very well, much better than the joint density of states from the original version of the manuscript. However, the convergence study for the GW corrections (see Fig. S1 and first line in Tab. S1) was performed with a technically not suitable k-point grid. As evidenced from the band structure in Fig. 1B, the Brillouin zone extension in y direction is about twice as long as in x and z direction. Therefore, the y direction should be sampled with twice as many k points as the x and z direction to ensure a homogeneous sampling of the reciprocal space. For unexplained reasons, the authors have used a set of k-point grids that fulfills this condition (see all but the first line of Tab. S1) and then chose to add the inappropriate 2x2x4 grid and conduct their GW convergence for this very grid. I acknowledge that this will not change the conclusions of the manuscript, since a PBE+G0W0 calculation with a plasmon-pole model is anyway not expected to reach experimental gap values (as the authors mention correctly) and the exact gap value is not a major point of discussion. However, I also do not want to leave a technically not sound convergence study uncommented.

We thank the referee for their careful assessment and positive comments about our new calculations and for pointing out this detail. We agree with the referee that the k-point grid of 2x2x4, which was used to perform the quasiparticle band gap convergence with respect to empty states and polarizability cutoff, does not follow the aspect ratio of the unit cell. However, as discussed extensively in prior literature (for example, in the Supporting Information of this reference by Malone and Cohen – DOI: 10.1088/0953-8984/25/10/105503), convergence with respect to the k-point grid density is independent of other parameters (i.e. the number of bands and polarizability cutoff). This means that the choice of grid for these convergence tests does not affect the convergence trends. In Table S1, we separately show that the quasiparticle band gap does not change significantly for other choices of k-point grids (comparing explicitly

2x2x4 with 2x4x2 and 4x8x4). Furthermore, we would note that the final GW calculation on which the spectrum reported in Figure 1D is based, is performed using a denser 4x8x4 k-point grid, which also better reflects the aspect ratio of the unit cell. Indeed, as the referee correctly acknowledges, the slight inconsistency they have highlighted will not change the final results and conclusions of our calculations.

Manuscript preparation issues:

** Figs. 1E, F still contain the old abbreviations CBB and VBT instead of CBM and VBM. Likewise, in the caption, there is still reference to the Y point of the Brillouin zone that is actually not depicted in the plane shown in the figures.*

** Caption of Fig. S3: The blue dashed line indicates the highest - not the lowest - occupied energy level.*

We thank the Reviewer for spotting these errors and their particularly careful proof reading of our manuscript, which we really appreciate!

We have now corrected these in the revised version.

The updated Figure 1 and its caption have been changed to:

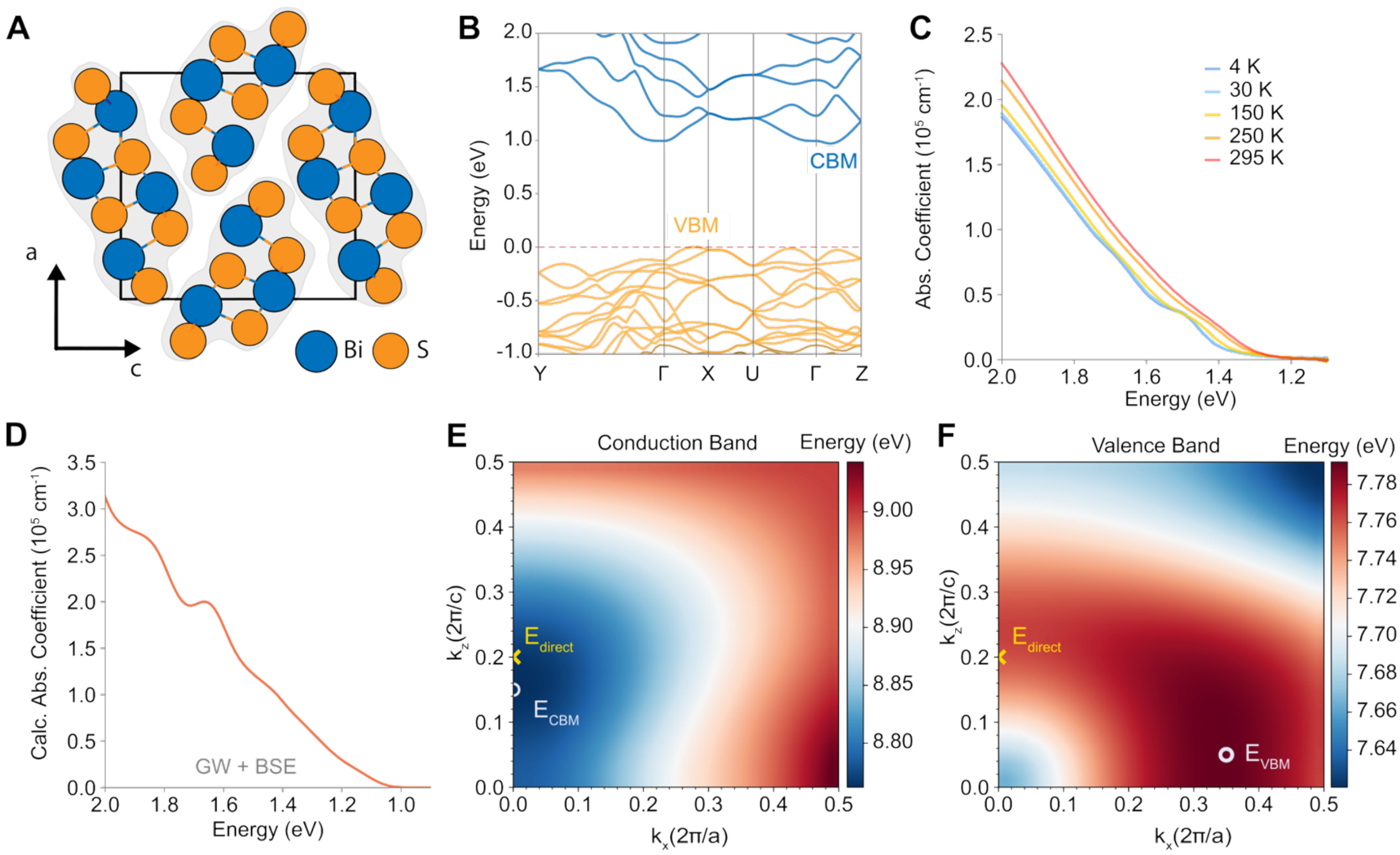

*Figure 1 | Electronic structure and absorption coefficient of Bi_2S_3 thin films. (A) Schematic diagram of the Bi_2S_3 crystal structure (**Pnma** space group) along the [010] direction. The shaded grey regions indicate the ribbon structures. The black square indicates the unit cell, containing two distinct ribbons. Blue and orange spheres represent bismuth and sulfur atoms, respectively. (B) Electronic band structure for Bi_2S_3, as calculated from DFT/PBE+SOC on the following high-symmetry path: Y(0, 1/2, 0) - Γ(0, 0, 0) - X(1/2, 0, 0) - U(1/2, 0, 1/2) - Γ(0, 0, 0) - Z(0, 0, 1/2), in units of the reciprocal lattice. The zero of the energy axis is set to the top of the valence band and indicated by a red dashed line. Low-energy direct electronic transitions are depicted on the electronic band structure in Figure S3. (C) Absorption coefficient for Bi_2S_3 thin films on quartz, measured in the NIR-Vis range for selected temperatures between 4 K and 295 K. The complete temperature-dependent absorption coefficient dataset is reported in Figure S4A. (D) Calculated linear optical absorption spectrum including only direct transitions, computed within the GW+BSE framework, including SOC, as described in detail in the SI. The onset of the calculated spectrum is red-shifted with respect to the measured onset by approximately 0.3 eV, consistent with typical underestimated quasiparticle band gaps computed within the G_0W_0 with a DFT/PBE starting point, extensively documented in the literature(58). (E), (F) Calculated DFT/PBE (including SOC) electronic bandstructure on the k_x-k_z BZ plane~~: Y(0, 1/2, 0) - Γ(0, 0, 0) - X(1/2, 0, 0) - U(1/2, 0, 1/2) - Z(0, 0, 1/2), in units of the reciprocal lattice~~. White circles are used to indicate the position of the valence band maximum (VBM) and the conduction band minimum (CBM). A yellow mark is used to indicate the position of the lowest-energy direct transition. The corresponding direct transition energies are shown in Figure S5.*

Reviewer #5

I co-reviewed this manuscript with one of the reviewers who provided the listed reports.

We are very grateful for the Reviewer's effort in reviewing our work.

Response to Third Review of Nature Manuscript 2026-03-06751 "Delayed Electron-Hole Recombination in a Pseudo-Direct Semiconductor" by Righetto et al.

We are very grateful for the highly positive feedback on the third version of our manuscript, and for the sign-off on publication from all five Reviewers. We have therefore in this round of revisions focused primarily on meeting the formatting requirements before publication. We have also added one final comment to the SI to satisfy the request by Reviewer 2, as detailed below. We hope that you may now find the revised version of our manuscript ready for publication.

Below, comments by editors and reviewers are given in *green italics*, our responses are given in black, and our highlighted revisions in the manuscript are given in *blue italics*. Deleted parts are given in ~~*brown italics*~~.

Response to Editorial Comments

(1) Your paper contains ~4,200 words, alongside with 3 figures, which is too long and over our word limit. Please reduce the length to a maximum of ~3,500 words. For technical details that are not central to the main message of the paper, please move them into the Methods section or Supplementary Information.

We have confirmed with you that the paper meets the required length following our cuts implemented in the last reviewer round.

The reference list should not contain more than 60 references in the main text. Please split your current list of references; additional references can be cited in (and listed after) the Methods section. Please note that Methods References should be below the Methods section, with numbering continuing on from the main text.

We have reduced the number of citations in the main text to comply with guidelines, and cited further references in the newly added Methods sections.

(2) We strongly support public availability of data and custom code. Please supply a Data Availability Statement, and/or a Code Availability Statement, and place the data and code used

in your paper into a public data repository, or alternatively, present the data as Supplementary Information. If data and code can only be shared on request, please explain why in your Data Availability Statement, and also in the correspondence with your editor. Please note that for some data types, deposition in a public repository is mandatory. Please see below for further information.

We thank the editor for their guidance, we have added a Data and Code Availability Statement. We will upload the data in the Oxford University Research Archive (ORA), where it will become publicly accessible through a DOI (to be supplied).

(3) Please familiarise yourself with our rules regarding the reproduction of images subject to third party rights. I would advise checking for potential rights issues in all figures. Figures that cannot be published would be better replaced at this stage rather than later.

We thank the Editor for the reminder, and we confirm that the current version of the manuscript contains no images subject to third-party rights.

Additional Editorial Changes:

1. We have reorganised the Supplementary Information to comply with Nature formatting, splitting the document into Extended Data (max 10 objects) and the Supplementary Information document containing the additional notes and other objects.
2. We have removed the main figures from the text document and supplied them as separate files.

Response to final reviewers' comments:

Reviewer #1

The authors have made efforts in addressing all remaining concerns raised in the previous round of review. I have carefully examined their point-by-point reply and the revised manuscript, and I am satisfied with the changes implemented. All editorial requirements have been fulfilled. The scientific content of the manuscript has been further improved in terms of

clarity, precision, and scope. I consider the manuscript in its present form to be suitable for publication in Nature and recommend its acceptance.

We thank the Reviewer again for all the valuable help they gave to further strengthen our manuscript.

Reviewer #2

The authors have satisfactorily addressed comments 5 and 6.

On comment 7, I think the revised, more cautious wording on device-efficiency gains is sufficient. I am still not convinced by the statement that a reciprocity-based radiative-limit Voc estimate would be "not meaningful". In principle, such an estimate should still be possible within the same generalized Planck/detailed-balance framework used in Supplementary Note 3. I would suggest that the authors either clarify this point or add a brief estimate in the SI.

This is a minor point and would not prevent publication. I am otherwise satisfied with the revision and support publication.

We thank the reviewer for the constructive scientific feedback on our response to comment 7 and have now added the requested clarification of this point to the discussion in Supplementary Note 3. We agree that meaningful calculations could be done on such systems in principle, provided all operating parameters are known for a system operating close to the radiative limit, which Bi_2S_3 currently does not, as discussed in previous rounds of the reviews. That said, for a solar cell operating close to (but not fully at) the radiative limit, we would not expect the introduction of an indirect transition just below the direct bandgap to lead to any increase in open-circuit voltage. Instead, we anticipate that the increase in mobility-lifetime product and charge-carrier diffusion lengths would lead to an increase in collected short-circuit current and/or fill factor given the increase in diffusion length that we have calculated. In addition, one major target of this concept is photo(electro)catalytic applications, where nanocomposite materials generally used exhibit relatively low charge-carrier mobilities. Here, pseudo-direct semiconductors should be particularly effective, since any enhancement in the mobility-lifetime product will greatly benefit charge-carrier diffusion lengths. A meaningful estimate of this increase in photovoltaic cells requires careful, detailed simulations that depend on a myriad

of factors, including long-range charge-carrier mobilities, doping density, built-in voltage, dielectric permittivity, direct and indirect absorption coefficients, contact properties, and a range of extrinsic loss parameters. For photocatalytic applications, detailed knowledge of nanomorphology, surface processes and reactions are required. Therefore, while we agree that full simulations are an interesting subject to pursue for Bi_2S_3, we feel that this is currently beyond the scope of this work.

We added a comment to SI Note 3 to clarify where we anticipate the impact to fall regarding improved solar cell performance as follows:

*"**Relation to photovoltaic performance.** The generalised Planck framework presented above also, in principle, permits estimates of the radiative-limit open-circuit voltage. Overall, in the presence of a direct-indirect bandgap combination, a boost in charge-carrier extraction and fill factor properties, close to the radiative limit, can be expected. Such gains may be particularly substantial for materials exhibiting lower long-range charge-carrier mobilities that are often common for inorganic semiconductors with layered structures."*

Reviewer #4

Fine for me.

We would like to thank the reviewer once again for their authoritative scientific feedback on our investigation and the revised manuscript.

Reviewer #5

I co-reviewed this manuscript with one of the reviewers who provided the listed reports.

We are very grateful for the Reviewer's effort in co-reviewing our work and for the scientific feedback provided.
